# Predicted Functional and Structural Diversity of Receiver Domains in Fungal Two-Component Regulatory Systems

**DOI:** 10.1128/mSphere.00722-21

**Published:** 2021-10-06

**Authors:** Robert B. Bourret, Clay A. Foster, William E. Goldman

**Affiliations:** a Department of Microbiology & Immunology, University of North Carolina, Chapel Hill, North Carolina, USA; b Department of Pediatrics, Section of Pediatric Hematology-Oncology, University of Oklahoma Health Sciences Center, Oklahoma City, Oklahoma, USA; University of Georgia

**Keywords:** fungi, hybrid histidine kinase, receiver domain, response regulator, two-component regulatory systems

## Abstract

Fungal two-component regulatory systems incorporate receiver domains into hybrid histidine kinases (HHKs) and response regulators. We constructed a nonredundant database of 670 fungal receiver domain sequences from 51 species sampled from nine fungal phyla. A much greater proportion (21%) of predicted fungal response regulators did not belong to known groups than previously appreciated. Receiver domains in Rim15 response regulators from Ascomycota and other phyla are very different from one another, as are the duplicate receiver domains in group XII HHKs. Fungal receiver domains from five known types of response regulators and 20 known types of HHKs exhibit distinct patterns of amino acids at conserved and variable positions known to be structurally and functionally important in bacterial receiver domains. We inferred structure/activity relationships from the patterns and propose multiple experimentally testable hypotheses about the mechanisms of signal transduction mediated by fungal receiver domains.

## OPINION/HYPOTHESIS

Two-component regulatory systems (TCSs) mediate signal transduction via transient protein phosphorylation. A generic TCS consists of a sensor kinase that monitors environmental conditions and a response regulator that implements appropriate responses to change. Conserved domains in both proteins catalyze phosphotransfer reactions. Transfer from ATP to a His residue on the sensor kinase transduces information into phosphoryl groups. Subsequent transfer to an Asp residue in the receiver domain of a response regulator modulates system output. Finally, transfer to water terminates the signal. In more complex versions, receiver and histidine-containing phosphotransfer (Hpt) domains inserted into the pathway between the sensor kinase and response regulator form a multistep phosphorelay. TCSs are organized differently in bacteria, archaea, fungi, and plants (1). Because vertebrate hosts do not encode TCSs, the systems are attractive targets for new antifungal drugs (2). Our knowledge of molecular signaling mechanisms employed by fungal TCSs is modest in comparison to that of bacterial TCSs, highlighting an opportunity where focused effort may potentially facilitate the development of entirely new types of antifungal agents. We describe a database of fungal receiver domains and initial findings that can drive future investigations of fungal TCSs.

**FIG 1:**
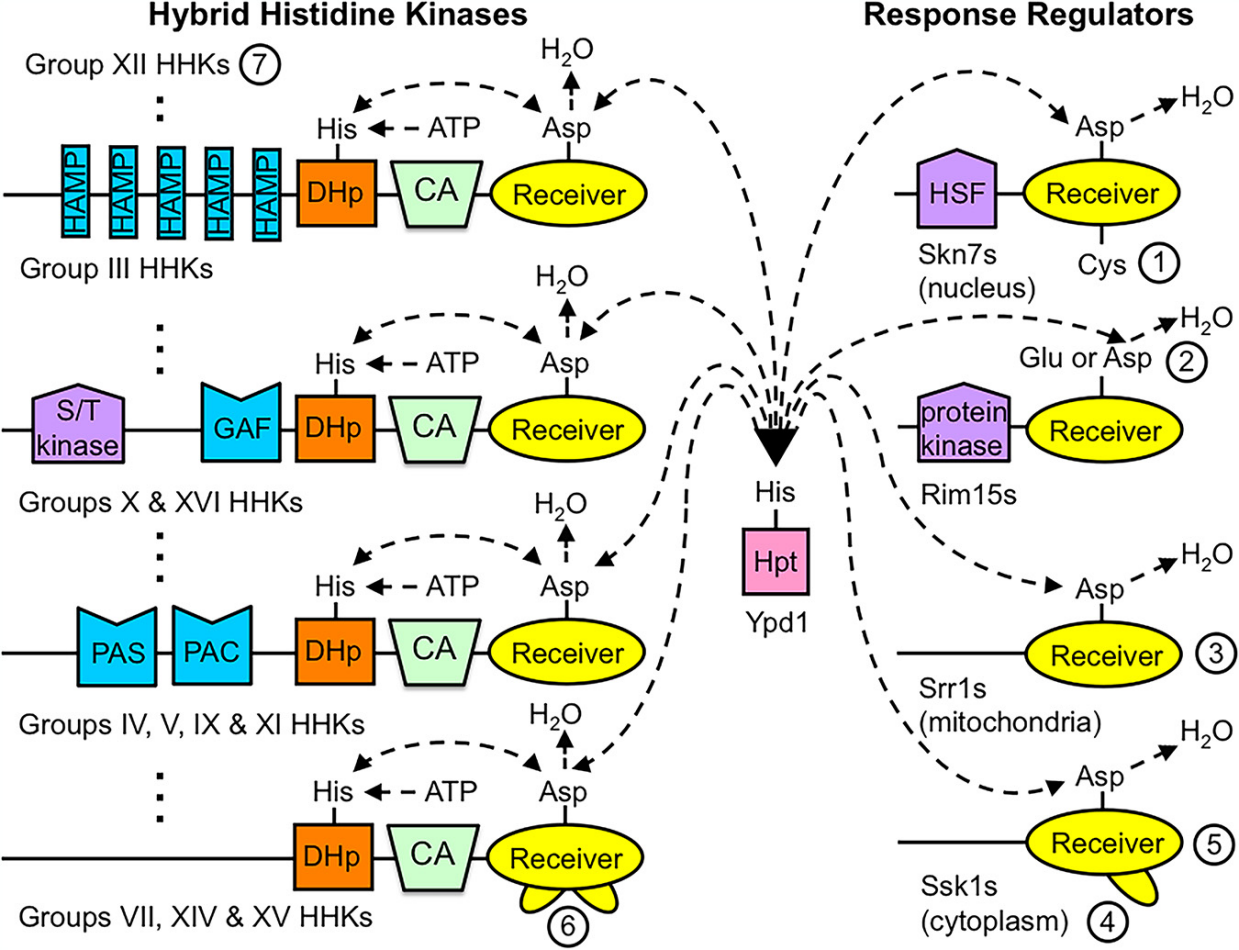
Architectures of fungal TCS proteins and hypotheses concerning receiver domain structure and function. Proteins are depicted from their N to C termini (left to right) but are not drawn to scale. In particular, the regions without identifiable domains (black lines) are often larger than the regions with identifiable domains. Conserved TCS domains include CA (catalytic and ATP binding), DHp (dimerization and His phosphorylation), Hpt (His-containing phosphotransfer), and Receiver. Dashed lines indicate potential phosphotransfer reactions, based on our knowledge of bacterial TCSs. However, very few phosphotransfer reactions have been experimentally demonstrated in fungal TCSs outside S. cerevisiae, and there are good reasons to question whether most signaling pathways funnel through Ypd1 (9). Fungal HHKs are sorted into 19 groups (I to XIX) based on sequence differences, including various domains in the N-terminal regions. Schematic diagrams of some HHKs are shown; the existence of others is indicated by vertical dots. There are four major types of fungal response regulators, as well as some unclassified regulators that are not shown. We demonstrate (Fig. 3) that the receiver domains in different types of fungal HHKs and response regulators exhibit distinct amino acid compositions at structurally and functionally important positions. Furthermore, the two receiver domains in group XII HHKs are different, as are the receiver domains in Rim15 proteins from Ascomycota and non-Ascomycota, leading to 25 different types of fungal receiver domains. Circled numbers refer to hypotheses described in the text. Hypothesis 1, a conserved Cys residue at DD+17 participates in Skn7 oxidative stress signaling (HSF, heat shock factor); hypothesis 2, signaling mechanisms for Rim15 receiver domains differ between fungal phyla; hypothesis 3, Srr1 utilizes modified allosteric communication between the active site and the α4β5α5 surface; hypothesis 4, Ssk1 binds to Ssk2 via an intrinsically disordered α3β4 loop; hypothesis 5, the surface of Ssk1 opposite the active site is functionally connected to phosphorylation; hypothesis 6, receiver domains of many HHKs bind to protein targets via the α2β3 and/or α3β4 loop; hypothesis 7, the two receiver domains of group XII HHKs have different functional roles.

The architecture of TCS proteins encoded by fungal genomes has been extensively cataloged (3–7) (Fig. 1). The vast majority of known sensor kinases in fungi contain a C-terminal receiver domain, referred to as a hybrid histidine kinase (HHK) and commonly associated with phosphorelays. Fungal HHKs can be sorted into at least 19 groups (labeled I to XIX) based on their attached variable domains and sequence motifs N terminal to the conserved catalytic phosphotransfer domains. There appears to be only one type of fungal Hpt, generally termed Ypd1 and not incorporated into unorthodox sensor kinases, as can happen in bacteria. The majority of fungal response regulators (i.e., proteins with receiver domains and lacking sensor kinase domains) fall within three groups, termed Rim15, Skn7, and Ssk1 after the best-characterized Saccharomyces cerevisiae homologs. A narrow group of fungi closely related to Candida albicans encode Ssr1-type response regulators (5), and a few unclassified response regulators have been reported (7, 8). Thus, fungal receiver domains exist in the context of at least 24 distinct protein architectures.

Many fundamental features of fungal TCSs remain to be discovered. We have noted that published data cannot distinguish between two fundamental hypotheses, that all fungal HHKs participate in phosphorelays and that only HHKs regulating the high-osmolarity glycerol (HOG) stress response participate in phosphorelays (9). Here, we focused on fungal receiver domains. Among the more than 19,000 unique protein domains catalogued in the Pfam database (10), receiver domains rank seventh (top 0.04%) in natural abundance. We created Data Set S1 in the supplemental material, containing 670 receiver domains from 51 fungal species of distinct genera encompassing nine fungal phyla. We took advantage of published studies that assigned HHKs to groups (3, 4, 6, 7, 11, 12) and expanded/refined classifications of all HHKs and response regulators encoded by these species as described in Text S1. Srr1 response regulators from an additional 18 species were also included (5). Examples of phylogenetic trees used by others to classify fungal HHKs and response regulators are in references 5, 6, 7, and 11. Table S1 is an inventory of receiver domains in the database by type. Text S2 contains all receiver domain sequences analyzed. We used the database to compare fungal receiver domains to those in their better-characterized bacterial counterparts and inspire experimentally testable hypotheses about functions and mechanisms.

10.1128/mSphere.00722-21.1 TEXT S1 Supplemental materials and methods. Download Text S1, PDF file, 0.1 MB. Copyright © 2021 Bourret et al. 2021 Bourret et al. https://creativecommons.org/licenses/by/4.0/ This content is distributed under the terms of the Creative Commons Attribution 4.0 International license.

10.1128/mSphere.00722-21.2 TEXT S2 Fungal receiver domain sequences used in this study. Download Text S2, PDF file, 0.2 MB. Copyright © 2021 Bourret et al. 2021 Bourret et al. https://creativecommons.org/licenses/by/4.0/ This content is distributed under the terms of the Creative Commons Attribution 4.0 International license.

10.1128/mSphere.00722-21.3 TABLE S1 Fungal receiver domain inventory. Download Table S1, PDF file, 0.04 MB. Copyright © 2021 Bourret et al. 2021 Bourret et al. https://creativecommons.org/licenses/by/4.0/ This content is distributed under the terms of the Creative Commons Attribution 4.0 International license.

10.1128/mSphere.00722-21.9 DATA SET S1 Spreadsheet of 25 selected characteristics for each of 670 individual fungal receiver domains. Download Data Set S1, XLSX file, 0.2 MB. Copyright © 2021 Bourret et al. 2021 Bourret et al. https://creativecommons.org/licenses/by/4.0/ This content is distributed under the terms of the Creative Commons Attribution 4.0 International license.

### Receiver domain amino acid sequences reflect structure and function.

**FIG 2:**
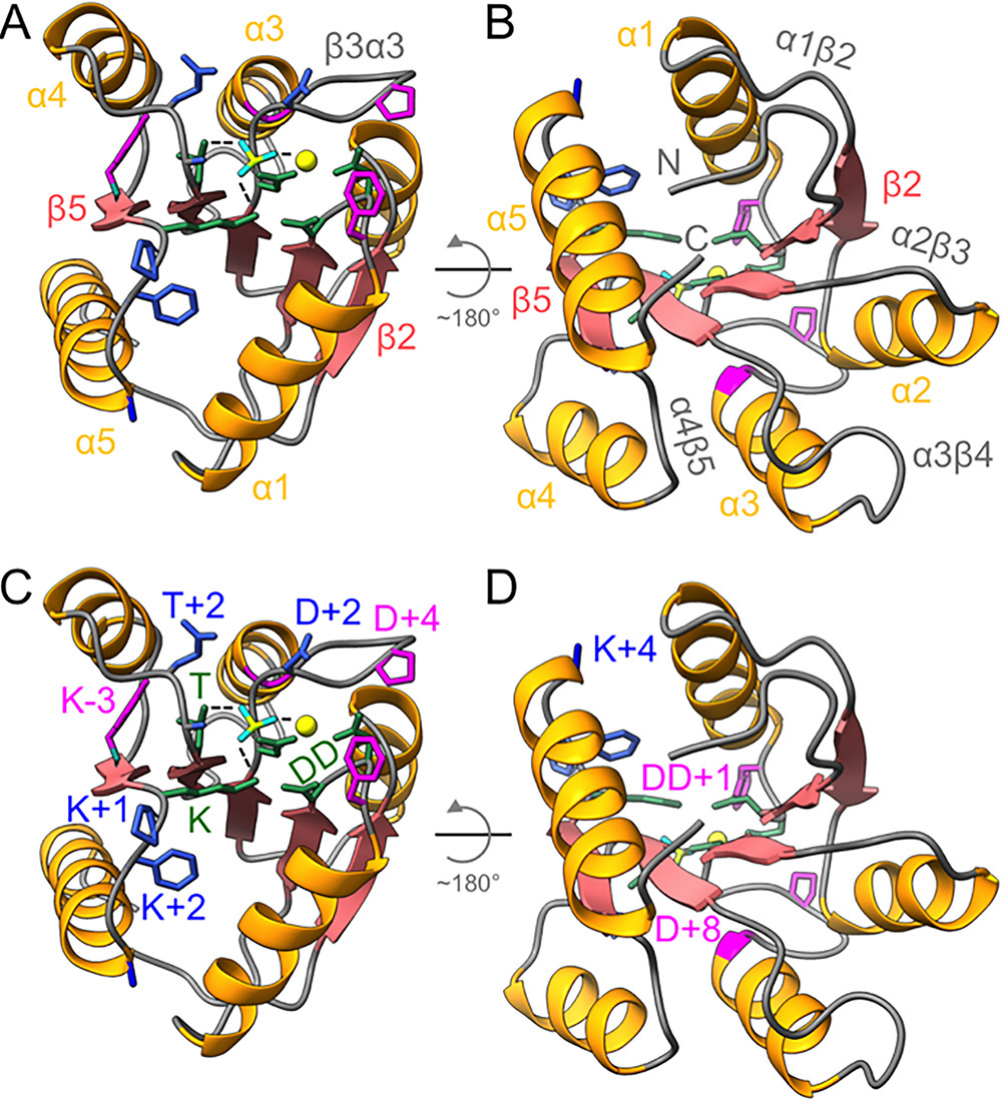
Key structural and functional features of receiver domains. A ribbon diagram of the model response regulator Escherichia coli CheY complexed with the stable phosphoryl group analog BeF_3_^−^ (PDB accession number 1FQW [51]) is shown. (A) View of the active site. The “phosphoryl group” is in cyan (oxygen atoms) and yellow (phosphorus atom). The metal ion is a yellow sphere. Known pathways of allosteric conformational change link the site of phosphorylation to the α4β5α5 surface via T and K–3 and to the α5α1 surface via K residues, including K+1, K+2, and K+4. (B) View of the surface opposite the active site. The N and C termini of the domain are indicated and represent points of connection to larger proteins containing the domain. Some fungal receiver domains (e.g., Ssk1, groups VII, VIII, XI, and XIII HHKs) have predicted intrinsically disordered sequences inserted into the α2β3 and α3β4 loops that we hypothesize are used to bind targets. (C, D) Side chains of the conserved active-site residues (DD, D, T, K) that catalyze phosphorylation and dephosphorylation reactions are in green (D is not labeled). Black dashed lines show coordination of the phosphoryl group oxygen atoms by K, T, and M^2+^. The variable residues known to affect reaction kinetics (D+2, T+1, T+2, K+1, K+2, K+4) are in blue (T+1 is not labeled and is located in front of T in panel C. Other residues important for structure (D+4, D+8) and function (DD+1, K–3) are in magenta.

To understand what fungal receiver domain sequences can teach us, we first review existing knowledge about bacterial receiver domains. The primary sequences of receiver domains encode alternating beta strands and alpha helices that fold into a (βα)_5_ structure featuring a central five-stranded parallel beta sheet flanked by five alpha helices (Fig. 2A and B) (13). The interior beta strands are easily identifiable in the primary sequence as runs of four consecutive hydrophobic residues. Sequence alignments of bacterial receiver domains reveal small differences in length that consistently occur in the loops connecting the secondary structural elements (13, 14). Closer inspection of existing structures suggests that the biggest architectural differences are in the lengths and orientations of α4 (15). Our database revealed that fungal receiver domains appear to exhibit more substantial variation in predicted loop lengths than in bacteria, suggesting significant functional consequences.

In bacterial receiver domains, five conserved residues located at the C-terminal ends of β1, β3, β4, and β5 (Fig. 2C and D) catalyze the phosphorylation and dephosphorylation of the Asp phosphorylation site (termed D) by stabilizing a planar transition state (reviewed in reference 16). Two acid residues (DD) bind a divalent metal cation (typically Mg^2+^) that stabilizes one of the phosphoryl group oxygens. Ser/Thr (T) and Lys (K) residues stabilize the other two oxygens of the phosphoryl group. About 10% of computationally identified receiver domains in bacteria lack one or more of the five conserved residues (17, 18). Such atypical (or pseudo-) receiver domains appeared to be less common in fungi (∼3%) (Table S1), with the exception of Rim15s.

Variable residues in bacterial receiver domains are named by the number of residues N (–) or C (+) terminal to the conserved catalytic landmarks. Six variable positions are known to affect the rates of phosphorylation and dephosphorylation reactions in bacterial receiver domains. Five of these appear to be functionally linked, as revealed by sequence covariation (19). T+1 directly controls physical access to the phosphorylation site (13, 20). D+2 and T+2 surround the path to the phosphorylation site and exert influence via hydrophobic/hydrophilic interactions with the reactants and products (18, 21, 22). K+1, K+2, and K+4 affect the equilibria between catalytically active and inactive conformations (19, 23). Different types of bacterial response regulators exhibit different combinations of amino acids at these variable positions, consistent with multivariate evolutionary tuning of reaction kinetics to suit their biological processes. Fungal receiver domains also exhibit sequence variability between types and often feature combinations at equivalent positions that are rare in bacteria, suggesting important functional differences between fungal and bacterial receiver domains.

The divalent metal bound by DD is essential for phosphotransfer reactions of bacterial receiver domains. Nevertheless, the affinity of different bacterial response regulators for cation varies by at least 2 orders of magnitude (15). Binding affinity is affected by the composition of DD (Asp Asp versus Glu Asp) and the chemical properties of DD+1 (24, 25). We are not aware of any investigations of metal binding by fungal receiver domains, although the receiver domain of S. cerevisiae Sln1 exhibits Mg^2+^ coordination that is indistinguishable from that of bacterial receiver domains (26). Limited diversity and characteristic patterns of amino acid composition at DD and DD+1 suggested that fungal receiver domains have evolved to support specific metal-binding characteristics.

Bacterial receiver domains exhibit bidirectional allostery between the active site and at least two distinct functional surfaces (16). Communication between the active site and the α5α1 surface likely involves changes in the positioning of the conserved K (23). In many response regulators, movement of the T position to bind a phosphoryl group oxygen is coupled with burying an adjacent aromatic residue at K–3, thus altering the α4β5α5 surface. Many fungal receiver domains exhibit amino acids at K–3 that are uncommon in bacteria. As described later, there are reasons in addition to K–3 to propose that fungal receiver domains may exhibit unique allosteric networks not known to occur in bacteria.

As a final structural note, the β3α3 loop includes a γ-turn initiated by a Pro at D+4 and a Gly at D+8 to initiate α3 (13). These amino acids occur in bacterial receiver domains at frequencies of 88% and 91%, respectively. The Pro and Gly residue frequencies are even higher in fungal receiver domains, predicting general conservation of the hairpin turn structure.

### Fungal response regulators.

**FIG 3:**
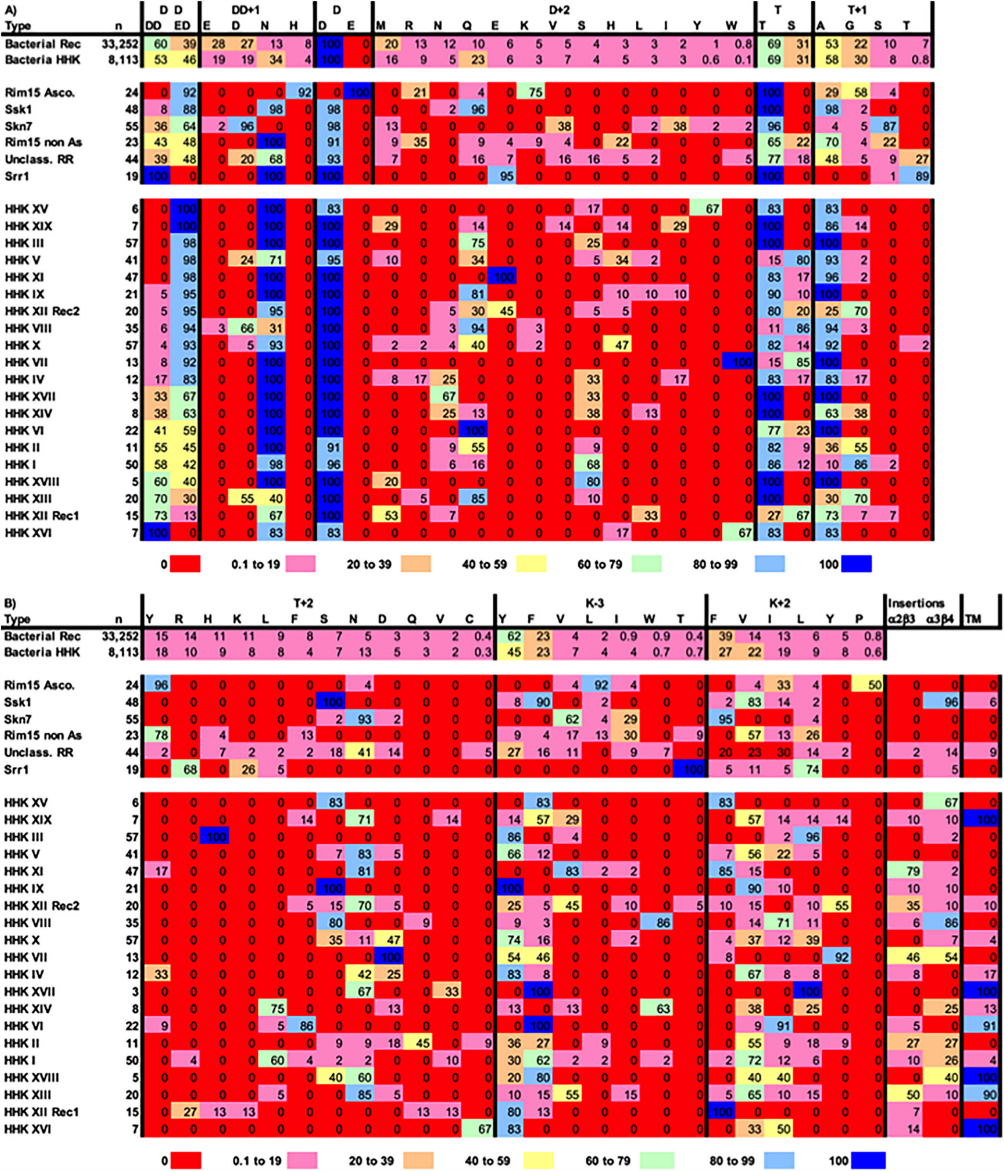
Heatmap of amino acid frequency at key conserved and variable receiver domain positions as a function of receiver domain type. In the first two lines, data from nonredundant databases representative of all bacterial receiver domains or bacterial HHKs are from reference 18. The rest of the figure is focused on fungal receiver domains and uses data from Tables S2 and S6. Positions are in N- to C-terminal order (from left to right), continuing from panel A to panel B. Positions at which there is little variation between fungal receiver domains (D, K, K+1, D+4, D+8) are not included. K+4 is included in the database but not in the tables or this figure because too little is currently known about the impact of amino acids other than Ala or Pro to meaningfully interpret the data. Within each position, specific amino acids are listed in order of decreasing abundance in bacterial receiver domains. Among included positions, amino acids that were not found in at least 20% of at least one type of receiver domain were excluded from the figure. Fungal receiver domain types are sorted within response regulator and HHK categories by decreasing abundance of Glu Asp at DD. Dark colors represent 0% or 100% abundance, whereas light colors represent quintiles in between. In panel B, insertions refer to the percentage of receiver domains within a type that contain insertions of 10 or more amino acids in the indicated loop, relative to the sequence of E. coli CheY. TM indicates the percentage of proteins that contain the indicated receiver domain type and one or more predicted transmembrane regions. Receiver domains themselves do not contain transmembrane regions.

Figure 3 and Table S2 in the supplemental material display the frequencies of amino acids found at the key positions described above, sorted by the five known types of fungal response regulators. Key variable residues appear to be much less diverse within response regulator types for fungi than within those for bacteria. However, at certain positions, fungi employ amino acids that are unconventional in bacteria. Aside from those in the “unclassified” category, fungal receiver domains exhibit type-specific patterns of functionally important residues. Additional comments about each type of response regulator follow.

10.1128/mSphere.00722-21.4 TABLE S2 Amino acid frequencies at key receiver domain positions in fungal response regulators. Download Table S2, PDF file, 0.05 MB. Copyright © 2021 Bourret et al. 2021 Bourret et al. https://creativecommons.org/licenses/by/4.0/ This content is distributed under the terms of the Creative Commons Attribution 4.0 International license.

10.1128/mSphere.00722-21.8 TABLE S6 Amino acid frequencies at key receiver domain positions in fungal hybrid histidine kinases. Download Table S6, PDF file, 0.08 MB. Copyright © 2021 Bourret et al. 2021 Bourret et al. https://creativecommons.org/licenses/by/4.0/ This content is distributed under the terms of the Creative Commons Attribution 4.0 International license.

### Hypothesis 1. A conserved Cys residue at DD+17 participates in Skn7 oxidative-stress signaling.

Skn7 is a nuclear transcription factor which responds to at least two types of inputs (reviewed in reference 27). First, Skn7 exchanges phosphoryl groups with HHKs located in the cytoplasm or membrane via the Hpt protein Ypd1 (28). Most Skn7s carry Asp at DD+1 and Ser at T+1 (Fig. 3), likely enhancing metal binding and dephosphorylation, respectively. Most Skn7s have Pro and Phe residues at K+1/K+2, the most frequent combination in bacteria. In contrast, the pairs of amino acids found at D+2/T+2 (Ile/Leu/Met with Asn) (Table S3) and the residues at K–3 (Ile/Leu/Val) (Fig. 3) are rare in bacteria. The respective functional consequences for reaction kinetics and phosphorylation-mediated conformational changes are unknown.

10.1128/mSphere.00722-21.5 TABLE S3 Most common D+2/T+2 pairs by fungal receiver domain type. Download Table S3, PDF file, 0.05 MB. Copyright © 2021 Bourret et al. 2021 Bourret et al. https://creativecommons.org/licenses/by/4.0/ This content is distributed under the terms of the Creative Commons Attribution 4.0 International license.

The Skn7-mediated response to oxidative stresses requires the receiver domain but is independent of Asp phosphorylation. A Cys-rich protein (Yap1 in S. cerevisiae) with an oxidation-sensitive conformation promotes phosphorylation of a Ser/Thr at D+10 in α3 of Skn7. However, 9% of the Skn7s in our database lack a Ser/Thr at D+10. Strikingly, 98% of Skn7s contain a Cys residue at DD+17 in the α1β2 loop distal to the receiver domain active site (Fig. 2A and B). We hypothesize that the DD+17 Cys is directly involved in redox signaling by Skn7. Asp phosphorylation is not known to affect the location of the Cys residue, consistent with multiple independent signaling functions in Skn7. Oxidation of the Cys may affect Skn7 dimerization or alter interactions between the receiver domain and a partner protein or the DNA binding domain of Skn7.

Another prominent feature of Skn7 is the consistent presence of an Arg/Lys residue at K+4 (Data Set S1), a position known to affect equilibria between active and inactive conformations (23). However, a lack of data makes it challenging to predict the functional consequences of basic residues at this position.

### Hypothesis 2. Signaling mechanisms for Rim15 receiver domains differ between fungal phyla.

Rim15 is a protein kinase that regulates cell division in response to nutrient levels (reviewed in reference 29). The role of the C-terminal receiver domain has apparently not yet been studied. However, a spontaneous mutant that truncates S. cerevisiae Rim15 in α3 ablates function, suggesting that the receiver domain is functionally important (30).

Our database reveals striking phylogenetic differences in Rim15 receiver domains. Among the 25 species from Ascomycota that contribute Rim15 entries, three well-studied model organisms differ from the rest. The S. cerevisiae and C. albicans Rim15 proteins, respectively, feature Ala and Gly in place of the phosphorylatable Asp. The Schizosaccharomyces pombe Rim15 proteins (Cek1 and Ppk31) lack receiver domains entirely. The remaining Rim15 entries from Ascomycota contain a Glu residue instead of the Asp phosphorylation site and feature a His residue at DD+1 and a Leu residue at K–3. Half have a Pro residue at K+2. These residues are unique to Rim15 from Ascomycota. Descriptions of Rim15 receiver domains in the literature note the Asp-to-Glu substitution. In bacterial receiver domains, this often results in partial constitutive activity (17). We have previously proposed a possible mechanism by which a fungal HHK may activate Rim15 in Ascomycota (9).

In contrast, Rim15 entries from Basidiomycota and Mucoromycota all contain Asp phosphorylation sites and an Asn residue at DD+1. Their receiver domains are similar to those of the bacterial paradigm, except for featuring nonaromatic residues at K–3 (commonly termed a “switch” residue in the bacterial TCS literature). We hypothesize that the signaling mechanisms used by Rim15 receiver domains differ between phyla and that there are likely to be both phosphorylation-dependent and -independent mechanisms. To the best of our knowledge, this is the first description of distinct types of Rim15 receiver domains. Finally, all Rim15 receiver domains in the database encode a modified α4 helix, most commonly shortened by 4 amino acids (one turn of a standard helix) compared to the length of Escherichia coli CheY.

### Hypothesis 3. Srr1 utilizes modified allosteric communication between the active site and the α4β5α5 surface.

Srr1 is encoded in the nucleus, is located in the mitochondria, and is more closely related to bacterial response regulators than the other TCS proteins in C. albicans (31). A phosphodonor for Srr1 has not been identified. Two sequence features of Srr1s are apparent. First, T+1 is exclusively a Thr/Ser residue which is likely to retard phosphorylation and enhance dephosphorylation reactions (20). Second, K–3 is exclusively a Thr residue, rarely seen (0.5%) at the equivalent position in bacteria. However, 74% of K–4 residues are Tyr/Phe/His, types typically found at K–3 in bacterial receiver domains. We therefore hypothesize that allosteric coupling between the phosphorylation site and the α4β5α5 surface will be distinct from that previously characterized for bacteria (16). Possibilities include the following: (i) the position of β5 may be shifted in Srr1 so that the aromatic residue at K–4 buries in a manner analogous to that of K–3 in bacterial receiver domains or (ii) the nature of the Srr1 pocket allows for embedding the hydrophilic Thr at K–3. A large residue at T+8, which occludes the buried aromatic residue at K–3 in bacterial receiver domains, is maintained in Srr1s. The buried K–3 residue is flanked by T+11 and K–1 in bacteria. T+11 is small (mostly Ala/Gly) in bacteria and large (Phe/Tyr) in Srr1s. The natures of K–1 differed between bacteria (50% Thr/Leu/Val) and Srr1 (53% Pro/Gly). In bacteria, position K–6 reciprocally covaries in size with K–3 (13). In Srr1s, the residue at K–6 is larger (Ile/Phe instead of Ala/Gly), while the residue at K–3 is smaller (Thr instead of Phe/Tyr). Thus, amino acid frequencies suggest that the size of the pocket is reduced in Srr1 (consistent with burying a smaller Thr instead of the typical aromatic seen at K–3 in bacteria) but retains hydrophobicity. This combination of properties may explain why no replacement with Ser was observed at K–3, because the additional methyl group may partially mitigate the polar nature of the Thr side chain. X-ray crystal structures of Srr1 in the presence and absence of the phosphoryl group analog BeF_3_^−^ would be illuminating.

### Unclassified fungal response regulators may be more common than is currently appreciated.

Excluding the narrowly distributed Srr1s, response regulators in the database are evenly distributed among Rim15, Skn7, Ssk1, and Unclassified types (Table S1). The proportion of Unclassified response regulators is much greater than generally recognized, perhaps due to uneven phylogenetic distribution. About 65% of the species in our database contain no Unclassified response regulators. Unclassified response regulators are present in only 24% of representatives of the Ascomycota and Basidiomycota but in 64% of representatives from seven other phyla. Furthermore, only 32% of the Unclassified response regulators come from Ascomycota and Basidiomycota, whereas another 36% come from just two species: Catenaria anguillulae and Mortierella elongata.

Unlike with other types of fungal response regulators, the key features of Unclassified receiver domains are diverse (Fig. 3), suggesting the possibility of additional subcategories. Approximately 27% contain deletions, degenerate sequences, or missing conserved residues compared to typical receiver domains (Table S1). It is not known if these putative proteins are functional and expressed or if they represent sequencing errors. In the only experimental investigation of which we are aware, deletion of CheteRec1 results in no observed phenotype (8). Most Unclassified response regulators exhibit an architecture of a receiver domain without an output domain, suggesting signal output via protein/protein interactions or participation in a phosphorelay. Interestingly, most archaeal response regulators also contain a single domain (32). However, a few Unclassified fungal response regulators incorporate putative enzymatic domains (Table S4) that may generate output.

10.1128/mSphere.00722-21.6 TABLE S4 Unclassified fungal response regulators with identified domains. Download Table S4, PDF file, 0.04 MB. Copyright © 2021 Bourret et al. 2021 Bourret et al. https://creativecommons.org/licenses/by/4.0/ This content is distributed under the terms of the Creative Commons Attribution 4.0 International license.

### Ssk1 binds to Ssk2 via an intrinsically disordered α3β4 loop (hypothesis 4), and the surface of Ssk1 opposite the active site is functionally connected to phosphorylation (hypothesis 5).

Ssk1 controls the HOG pathway, which is widespread in fungi (33). Details vary between species, but a phosphorelay involving HHKs, Ypd1, and Ssk1 controls the HOG response. The amino acids found at key positions in Ssk1s (Table S2) are reminiscent of bacterial receiver domains and are compatible with phosphorelays. The limited variation between Ssk1s (Fig. 3) is consistent with the constraint that misregulation of the HOG pathway is generally lethal.

Our database inspires two hypotheses for how Ssk1 carries out its regulatory function. The key unresolved issues are (i) how does Ssk1 separately bind to its upstream partner Ypd1, itself, or its downstream partner Ssk2 and (ii) how does phosphorylation of Ssk1 influence some these binding events but not others? S. cerevisiae Ssk1 binds to Ypd1 via the α1/α5 helices and the active site of the receiver domain (34), forming a relatively stable heterodimer. Substitutions in Ssk1 α1 that prevent binding to Ypd1 do not block binding to Ssk2, implying different binding interfaces (35). When osmolarity increases, Ssk1 dephosphorylates and forms an active homodimer. The receiver domain of the Ssk1 dimer then binds to and inactivates the autoinhibitory domain of Ssk2, which is at the start of the mitogen-activated protein (MAP) kinase cascade controlling Hog1 (35, 36).

Ssk1 was previously proposed to bind Ssk2 via its α4β5α5 surface (35). However, Ssk1 can function effectively only as a homodimer. The ability of Ssk1 to form a homodimer is independent of phosphorylation, but Ssk2 binding is strongly regulated by activation state (35–37). Interestingly, *in silico* studies suggest that the aromatic K–3 residue of Ssk1 remains exposed regardless of phosphorylation state (38), in contrast to the rotameric switching often seen in bacterial receiver domains. The common use of α4β5α5 as a protein binding or dimerization interface in bacterial receiver domains (16), the conservation of Phe/Tyr residues at K–3 in Ssk1s, and the correlation of phosphorylation independence for both Ssk1 dimerization and K–3 conformation imply that the α4β5α5 region likely contains the Ssk1 homodimerization interface.

So how then could Ssk1 bind to Ssk2? Conspicuously, Ssk1 receiver domains all contain extended (average, 38 ± 16) amino acid insertions in the α3β4 loop. The inserts are rich in Pro and Ser residues, consistent with intrinsically disordered protein regions often involved in protein/protein interactions (39), and are not visible in the S. cerevisiae Ssk1·Ypd1 cocrystal structure (34). The face of Ssk1 containing α3β4 also connects to a large N-terminal region of unknown function (Fig. 2B). Inclusion of the Ssk1 N-terminal region substantially enhances binding to Ssk2 compared to the binding of the Ssk1 receiver domain alone (35). Based on the evidence, we hypothesize that Ssk1 binds to Ssk2 via the extended α3β4 loop. However, the possibility that the actual roles of the α4β5α5 face and α3β4 loop are the reverse of this hypothesis should not be dismissed without further investigation, because an Asp-to-Gly substitution at K–4 in β5 of Ssk1 blocks Ssk2 binding but not homodimerization (35). In any case, the two known allosteric pathways in receiver domains connect the phosphorylation site to the surface used by Ssk1 for Ypd1 binding and the α4β5α5 face but not the α3β4 loop. Consequently, we further hypothesize that an as-yet-uncharacterized mechanism connects phosphorylation to a protein-binding event on the opposite side of Ssk1, likely related to the orientation of the loop. Consistently with this possibility, molecular dynamics simulations of S. cerevisiae Ssk1 suggest that phosphorylation shifts the positions of α2, α3, and α5 (38).

### Fungal hybrid histidine kinases.

Our database included 17 previously unclassified and 2 reclassified fungal HHKs (Table S5). Figure 3 and Table S6 display the frequencies in our database of amino acids found at key receiver domain positions in fungal HHKs.

10.1128/mSphere.00722-21.7 TABLE S5 HHK group assignments made in this work. Download Table S5, PDF file, 0.04 MB. Copyright © 2021 Bourret et al. 2021 Bourret et al. https://creativecommons.org/licenses/by/4.0/ This content is distributed under the terms of the Creative Commons Attribution 4.0 International license.

### Hypothesis 6. Receiver domains of many HHKs bind to protein targets via the α2β3 and/or α3β4 loop.

We previously proposed that receiver domains of some fungal HHKs bind to protein targets other than Ypd1 (9). A potential mechanism is provided by the observation that 31% of the HHK receiver domains in our database contain insertions of 10 or more amino acids (relative to the length of the model bacterial receiver domain E. coli CheY) in the α2β3 and/or α3β4 loop (Fig. 3B), reminiscent of Ssk1. The insertions are most abundant in groups VII, VIII, XI, and XV but are also common in groups I, II, XII Rec2, XIII, XIV, and XVIII. If the loops are not involved in target binding, then other functional explanations will be needed to account for the prevalence of insertions in fungal compared to bacterial receiver domains.

### Hypothesis 7. The two receiver domains of group XII HHKs have different functional roles.

Group XII HHKs generally contain a tandem duplication of two complete sets of hybrid kinase machinery (40). The Cryptococcus neoformans group XII HHK Tco2 contributes to the control of the HOG pathway (41), but there appears to be no published investigation of the signaling mechanisms employed by group XII HHKs themselves. The two receiver domains differ at many key positions (Fig. 3), suggesting distinct functional roles. Notably, the most N-terminal receiver domain (termed Rec1) is the only type of fungal receiver that utilizes the D+2/T+2 pairs most common in bacteria (Met Arg and Met Lys). These combinations are associated with slow autodephosphorylation and fast autophosphorylation (18, 22), suggesting a longer-lived signal. In contrast, group XII Rec2 domains frequently contain insertions in the α2β3 and/or α3β4 loop, suggesting additional target binding. About 26% of the group XII receiver domains in the database are in proteins with a single Rec1 or Rec2 domain. If group XII HHKs with single receiver domains represent real proteins rather than sequencing mistakes, then they may provide a natural opportunity to decipher distinct functional roles for Rec1 and Rec2.

### Amino acid abundances at key receiver domain positions differ between fungal HHKs and bacteria.

Receiver domains in fungal HHKs frequently utilize amino acids that are uncommon at the equivalent positions in their bacterial counterparts (Fig. 3). This provides numerous opportunities for future investigations to determine the functional and evolutionary significance of these changes. Examples follow.

- At position DD, Glu Asp is a more common residue pair than Asp Asp in fungal HHK receiver domains. The reverse is true of bacterial receiver domains. Furthermore, DD+1 is mostly Asn in fungi and does not utilize an Asp residue, which is the most common amino acid at DD+1 in bacteria. These differences may affect the affinity/specificity of metal ion binding by fungal receiver domains.
- In contrast to fungal response regulators, fungal HHKs do not use Ser/Thr residues at T+1, thereby increasing access to the phosphorylation site (13, 20).
- Residue types at D+2 of fungal HHK receiver domains are mostly hydrophilic (with Gln/Ser being the most abundant). The amino acids at T+2 are more diverse than at D+2 but are also mostly hydrophilic (with Asn/Ser being the most abundant). Hydrophilic residues at D+2 and T+2 are predicted to enhance dephosphorylation (18).
- Phe is the most common residue type at K+2 in bacteria. Five types of fungal HHK receiver domains contain Phe residues or functionally similar Tyr residues at K+2. However, 15 types of fungal HHK receiver domains contain primarily an Ile, Leu, or Val residue at K+2, which is likely to increase the fraction of the population in active conformations and enhance phosphorylation (19).
- K–3 (involved in allosteric communication between the active site and the α4β5α5 surface) is mostly Tyr or Phe residues in bacteria, as well as in most types of fungal HHK receiver domains. However, Val is most common in groups XI, XII Rec2, and XIII, and Trp is most common in groups VIII and XIV. Notably, a Trp residue at K–3 results in partial constitutive activity in E. coli CheY (42).

### Receiver domains from different fungal HHK groups exhibit distinctive patterns of amino acids at key residues.

Continuing the trend observed in fungal response regulators, all fungal HHK receiver domain types exhibit different patterns of amino acid abundance across key residues (Fig. 3). Groups that share characteristic types of N-terminal domains (e.g., PAS/PAC domains [groups IV, V, IX, and XI], Ser/Thr kinase domains [groups X and XVI], or no identifiable domains [groups VII, XIV, and XV]) do not appear to share similar key residues. Even receiver domains from HHKs that cluster near one another in sequence-based phylogenetic trees (e.g., groups I, II, and XIV) (6) exhibit distinct differences. This suggests that receiver domains from different fungal HHKs have been selected for different functional properties during evolution.

### Transmembrane regions.

About 73% of bacterial sensor kinases contain transmembrane regions (43), whereas most fungal HHKs are predicted to be cytoplasmic (4, 6). We can quantify this difference, as only 15% of fungal HHKs in our database are predicted to localize to the membrane. There are many theories about how cytoplasmic fungal HHKs might detect environmental stimuli (9) but few published experimental data. Fungal HHKs with transmembrane regions are concentrated in groups VI, XIII, XVI, XVII, XVIII, and XIX (Fig. 3B), as previously noted (6).

### Concluding remarks.

Fungi are major pathogens of plants, animals, and humans (44). Because TCSs are ubiquitous in fungi yet absent in humans and animals, TCSs are potential targets for antifungal drugs (2). However, as pointed out here and in reference 9, fundamental functional features that are critical to both effectively target fungal TCSs (45) and avoid off-target consequences (46) remain unknown. Experimental data on TCSs in pathogenic fungi are remarkably limited and generally phenomenological rather than mechanistic. Although fungal TCSs were discovered almost 30 years ago, we are aware of only four published studies exploring the biochemical properties of purified TCS proteins from fungal pathogens (47–50). We hope that this article will inspire new investigators to turn their attention to fungal TCSs.
