# Supplementary material for: Predicted Functional and Structural Diversity of Receiver Domains in Fungal Two-Component Regulatory Systems
Source: mSphere. 2021 Oct 6;6(5):e00722-21. doi: 10.1128/mSphere.00722-21 (PMC8510515; doi:10.1128/mSphere.00722-21)
Supplement: TEXT S1 [file msphere.00722-21-s0001.pdf]

TEXT S1

Source genomes. We included in our database TCS proteins encoded by genomes for which HHK group assignments had been published (1-6). This resulted in 50 fungal species, all from different genera. To avoid sample bias, we included *Aspergillus fumigatus,* but did not include available information on other *Aspergillus* species (7, 8). To include another fungal phylum, we added *Piromyces finnis* (9). For reference purposes, two non-fungal species (*Capsaspora owczarzaki* and *Monosiga brevicollis*) that are evolutionarily between fungi and metazoans and were classified by Kabbara et al. (4) are included in the database, but excluded from analysis in the main text.

Hybrid histidine kinase (HHK) sources. Most HHKs and group assignments initially came from accession numbers in the publications cited in the previous paragraph. Where possible, these were linked to UniProtKB IDs.

Because of developments in genome sequencing, some data from Lavin *et al.* (5) and Kabbara *et al.* (4) are now outdated and could not be easily located in the current Joint Genome Initiative (JGI) database. We obtained legacy draft versions of the following proteomes: catan1, conco1, ganpr1, gloin1, morel2, phchr1/phchr2, rambr1, rozal1, sporo1, synplu1, umbra1, usnflo1, and xylhe1. Proteomes were accessed from the JGI MycoCosm portal (https://mycocosm.jgi.doe.gov/). Where possible, complete proteomes were downloaded as .fasta files (not the filtered versions). Proteomes were parsed for the deprecated accession numbers extracted from Lavin *et al.* Table 2 and Kabbara *et al.* Table S1. Each entry found a one-to-one match in the corresponding proteome and the matching HHK sequences were extracted. Sequences were

randomly spot-checked to confirm that the proteins belonged to the appropriate fungal HHK groups. Almost all HHKs were successfully identified, with only the following minor ambiguities:

- The group III HHK RirreHHK1 is listed in Table S1 of (4) with an accession number of Gloin1|932836, but has an actual accession number of Gloin1|93283.
- The final UniProt sequences for PchryHHK1, PchryHHK2, PchryHHK3, and PchryHHK5 differed slightly from the original respective entries Phchr2|2917000, Phchr1|130647, Phchr1|2663, and Phchr1|7364 listed in Table 2 of (5).
- RirreHK2 and RirreHK3 are listed in Table S1 of (4) with accession numbers of Gloin1|93941 and Gloin1|90992 respectively, but the sequences we retrieved were far too short for Group XII-B dual HHKs. Thus, the RirreHK2 and RirreHK3 entries remain blank in our database.
- We retrieved a sequence Sporo1|26075 that encodes a group III HHK about 2700 amino acids in length with 10 C-terminal transmembrane regions. We did not include this putative HHK in the database because (i) in all other fungal HHKs with identified transmembrane regions, the transmembrane regions are N-terminal (or occasionally in the middle) rather than C-terminal and (ii) the sequence is not listed in the Sporo1 genome on JGI. Both points suggest Sporo1|26075 may have been a sequencing mistake.

HHK classification. Schmoll *et al.* (6) assigned 10 HHKs from *Hypocrea jecorina* to groups, but the accession numbers published in 2008 are no longer easily available. We unambiguously assigned the 10 *H. jecorina* HHKs found in UniProt to groups using domain composition and the scale drawings in Figure 6 of Schmoll *et al.*

The HHK classification scheme used by Lavin et al. (5) has been superseded. Assignments were converted as follows. According to Defosse *et al.* (2), SGD-HK is group I, IA is group III, Fph is group VIII, and IC is group IX. It is obvious that STK HK is group X and Dual HK is group XII. According to Kabbara et al. (4), Dual HK from basidiomycetes belong to group XII-B and IB is group XIII. Similarly, the IB-like, MS-HK I, and MS-HK 2 designations introduced by Defosse *et al.* and used in their Supplemental Sequences now correspond to groups XVII, XV, and XVI respectively according to Kabbara *et al.*

CheteHK10 was classified in group I by Catlett et al. (1) in 2003, but segregated by itself and not with group I HHKs in the phylogenetic tree made by Jacob et al. (3), presumably because the availability of more HHK sequences in 2014 provided higher resolution. We suggest CheteHK10 actually belongs to group XIV based on its position within phylogenetic trees made from known group I and XIV sequences (data not shown). Similarly, TasahHK7 was assigned to group VII in (2), but the amino acids at key positions were very different than in other group VII HHKs. A phylogenetic tree made from known group representatives suggests that TasahHK7 belongs to group XIII (data not shown), a categorization that is supported by both the presence of transmembrane regions and a close match to key residues in multiple group XIII receiver domains.

There appear to be two inconsistencies between the phylogenetic tree in Figure 2 and the HHK architectures in Table S1 of Kabbara *et al*. (4). Figure 2 shows RirreHK5 as group II and Rirre HK8 as group XIII. Similarly, Figure 2 shows SplumHK1 as group XII-B and SplumHK3 as group XIII. The group assignments within each pair appear to

be swapped and are treated as such in our database. Our assignments were verified by the presence of predicted transmembrane regions in the group XIII proteins RirreHK5 and SplumHK1, and the absence of transmembrane regions in RirreHK8 and SplumHK3.

The most recent HHK classification scheme of Kabbara et al. reveals that groups X, XII, and XVIII each contain two or three subgroups (designated -A, -B, -C). Our database contains subgroup listings where known, but does not attempt to assign older entries to current subgroups, unless flanking GAF or PAS domains were present to distinguish between XII-A and XII-B.

Most group XII HHKs are tandemly duplicated HHKs, with two HisKA, two HATPase_c domains, and two Rec domains. We term the N-terminal receiver domain Rec1 and the C-terminal receiver domain Rec2. Rec1 and Rec2 type receiver domains of group XII HHKs generally exhibited different amino acids at the key positions examined in this study. There were nine group XII HHKs with only one receiver domain. We assigned the receiver domains to be Rec1 or Rec2 as follows:

- RirreHK14 was almost a complete tandem duplication, only lacking the C-terminal Rec domain. Therefore, we designated the receiver domain of RirreHK14 as Rec1 type.
- The other eight group XII HHKs were single HHKs. RirreHK10 had a different sequence than RirreHK14, but the receiver domains are identical at the 15 key positions examined in this study. Therefore, we designated the receiver domain of RirreHK10 as Rec1 type.

- The receiver domain of RirreHK11 matched MelonHK3 Rec2 at 13 of 15 key positions. Therefore, we designated the receiver domain of RirreHK11 as Rec2 type.
- The receiver domain of SroseHK9 matched CneofTCO4 Rec2 at 14 of 15 key positions. Therefore, we designated the receiver domain of SroseHK9 as Rec2 type.
- The receiver domain of UramHK12 matched the Rec2 domains of MlariHHK3, PgramHK1, and SroseHK3 at 11 of 15 key positions. Most notably, the D+2/T+2 pair of Gln Asn was present in all four receiver domains, and absent from all group XII Rec1 domains. Therefore, we designated the receiver domain of UramaHK12 as Rec2 type.
- The receiver domain of SplumHK3 matched the receiver domain of UramaHK12 at 13 of 15 key positions. Therefore, we designated the receiver domain of SplumHK3 as Rec2 type.
- The receiver domain of UramaHK13 was atypical and therefore exhibits reduced identity to other receiver domains. The closest match was with UramaHK12, sharing 10 out of 15 key residues. Confidence in designating the receiver domain of UramaHK13 as Rec2 was enhanced by the fact that D+2/T+2 was Gln Asn.
- The receiver domain MelonHK13 matched MelonHK3 Rec2 at 12 of 15 key positions. Therefore, we designated the receiver domain of MelonHK13 as Rec2 type.
- The closest match of key positions for the receiver domain of RbrevHK2 was only 10 out 15. We designated the RbrevHK2 receiver domain as Rec2 type based on (i)

the polar His Asp D+2/T+2 pair (reminiscent of Gln Asn pairs in Rec2s) and (ii) the presence of a large insertion (only found in Rec2s).

We identified 16 HHKs that were not previously included in HHK classification schemes from the same species (Table S5). Some may have been overlooked or excluded because they lack HisKA or HATPase_c domains. We assigned them to groups by making phylogenetic trees that included representatives of all 19 groups and seeing where the unclassified HHKs segregated. Specifically, classified and unclassified sequences were combined and aligned using the L-INSI-I algorithm in MAFFT (v.7.397) (10). The alignment was then used to create a maximum likelihood phylogenetic tree built with PhyML (11) using the LG substitution model (12), empirical amino acid frequencies, four substitution categories, an estimated gamma shape parameter, and an estimated proportion of invariable sites (LG+G+I+F; these parameters were predicted to be optimal for the particular set of sequences analyzed) (13). We then confirmed assignments by comparing amino acids at key receiver domain positions to known members of various groups. We were unable to classify RirreHK12, which contains a truncated receiver domain

Response regulator sources. All Srr1 entries came from (14).

Some publications cited as HHK sources also contain accession numbers for response regulators. However, most response regulators were collected by database searches. UniProt (https://www.uniprot.org) was searched for the species name and the term "receiver". If response regulators were not found in UniProt, then the same search was conducted in the NCBI Protein database (https://www.ncbi.nlm.nih.gov).

Genomes not found in either UniProt or NCBI were searched in the JGI MycoCosm database. Species in MycoCosm were browsed to find all entries containing a receiver domain (PF00072).

Response regulator classification. Response regulators other than Srr1 were classified as follows. The presence of Ser/Thr kinase domains (particularly STKc_Rim15_like, NCBI cd05611) identified Rim15 proteins. The presence of an HSF_DNA-bind domain (PF00447) identified Skn7 proteins. The presence of an unusually long receiver domain (compared to *E. coli* CheY), combined with process of elimination (many fungal genomes encode one each of Rim15, Skn7, and Ssk1) identified Ssk1 proteins. The consistent and distinct sequence features displayed in Figure 3 and Table S2 for Rim15, Skn7, and Ssk1 receiver domains provide confidence in the robustness of our classification method. As described in the main text, we identified 44 response regulators that did not appear to belong to any of the four main types and were designated Unclassified.

Nomenclature. Much of the fungal TCS literature incorporates the first letters of the genus and species names into protein names (e.g. CaSln1 for *Candida albicans* Sln1). However, the proliferation of genome sequences has made this convention ambiguous. Therefore, we used the convention suggested in (4), in which the first letter of the genus is combined with the first four letters of the species to generate a unique genus/species prefix. The names of proteins identified in previous publications were retained when known. Otherwise, the prefix was followed by HHKn for HHKs, RRn for Unclassified response regulators, and Rim15, Skn7, or Ssk1 for response regulators of the corresponding types. The names of established response regulator classes end in

numerals, so multiple response regulators of the same type in a single species were distinguished by suffixes A, B, etc.

Obtaining receiver domain sequences. Sequences corresponding to the receiver domain were selected automatically in UniProt where possible. Otherwise, we manually selected the sequence beginning one residue N-terminal to β1 through residue K+16. All receiver domain sequences are available in List S1.

Predicted transmembrane regions. The number of transmembrane regions predicted for each protein were generally taken from the UniProt or JGI listings for the complete protein. Some were taken from the schematic diagrams in Table S1 of (4). Finally, some were obtained by analyzing the protein sequence using the SMART website (http://smart.embl-heidelberg.de).

Multiple sequence alignments. Multiple sequence alignments of receiver domains from the same type of TCS protein were constructed using the Clustal Omega (1.4.2) (15) tool on UniProt. All alignments were made with respect to *E. coli* CheY as a common reference point. For receiver domains with large insertions of varying length and sequence, the default choices for gap penalties resulted in alignments that did not make sense with what is known about receiver domain structure and function. In such cases, we made manually adjustment to align the β4 and β5 strands. The lengths of insertions or deletions in the α1β2, α2β3, and α3β4 loops compared to CheY are included in the database. Many entries list a one amino acid deletion in α1β2 and a one amino acid insertion in α2β3. These may be artifacts of the particular sequences included in a given alignment. Amino acids at each of 15 specific conserved and

variable positions were identified from the multiple sequence alignments, using the conserved residues of CheY as landmarks.

Fungal phylogeny. Species in Database S1 are assigned to the nine fungal phyla described in (16). However, fungal classification schemes are regularly updated due to the availability of additional DNA sequence data. James et al. (17) propose 12 fungal phyla, including two that currently contain no completely sequenced genomes and thus would be unsuitable for inclusion in an analysis of complete sets of TCS proteins at the species level. A recent analysis of >1600 fungal genomes further refined fungal classification (18). Users can easily modify Database S1 to include additional or updated taxonomic ranks.

Database key. Red shading indicates divergent residues at conserved positions, including the absence of the residue entirely (indicated by X) or inability to identify the residue (indicated by ?). Orange shading indicates uncommon residues at variable positions, or expected proteins that could not be located. Purple shading indicates information from non-fungal proteins. Cyan shading indicates proteins for which a UniProt entry could not be located. Insertions or deletions not located in the $\alpha1\beta2$, $\alpha2\beta3$, or $\alpha3\beta4$ loops are noted in the Comments column. For example, "1 a4b5" means an insertion of one amino acid in the $\alpha4\beta5$ loop and "minus 4 a4" means a deletion of four amino acids in the $\alpha4$ helix.

Corrections requested. Database S1 consists of ~17,000 cells, all of which were populated manually. Please send any corrections or suggestions for improvement to Bob Bourret (bourret@med.unc.edu), who is solely responsible for any errors.

REFERENCES

1. Catlett NL, Yoder OC, Turgeon BG. 2003. Whole-genome analysis of two-component signal transduction genes in fungal pathogens. Eukaryot Cell 2:1151-61.
2. Defosse TA, Sharma A, Mondal AK, Duge de Bernonville T, Latge JP, Calderone R, Giglioli-Guivarc'h N, Courdavault V, Clastre M, Papon N. 2015. Hybrid histidine kinases in pathogenic fungi. Mol Microbiol 95:914-24.
3. Jacob S, Foster AJ, Yemelin A, Thines E. 2014. Histidine kinases mediate differentiation, stress response, and pathogenicity in *Magnaporthe oryzae*. Microbiologyopen 3:668-87.
4. Kabbara S, Herivaux A, Duge de Bernonville T, Courdavault V, Clastre M, Gastebois A, Osman M, Hamze M, Cock JM, Schaap P, Papon N. 2019. Diversity and evolution of sensor histidine kinases in Eukaryotes. Genome Biol Evol 11:86-108.
5. Lavin JL, Ramirez L, Ussery DW, Pisabarro AG, Oguiza JA. 2010. Genomic analysis of two-component signal transduction proteins in basidiomycetes. J Mol Microbiol Biotechnol 18:63-73.
6. Schmoll M. 2008. The information highways of a biotechnological workhorse--signal transduction in *Hypocrea jecorina*. BMC Genomics 9:430.
7. Azuma N, Kanamaru K, Matsushika A, Yamashino T, Mizuno T, Kato M, Kobayashi T. 2007. *In vitro* analysis of His-Asp phosphorelays in *Aspergillus nidulans*: the first direct biochemical evidence for the existence of His-Asp phosphotransfer systems in filamentous fungi. Biosci Biotechnol Biochem 71:2493-502.
8. Hagiwara D, Sakamoto K, Abe K, Gomi K. 2016. Signaling pathways for stress responses and adaptation in *Aspergillus species*: stress biology in the post-genomic era. Biosci Biotechnol Biochem 80:1667-80.
9. Haitjema CH, Gilmore SP, Henske JK, Solomon KV, de Groot R, Kuo A, Mondo SJ, Salamov AA, LaButti K, Zhao Z, Chiniquy J, Barry K, Brewer HM, Purvine SO, Wright AT, Hainaut M, Boxma B, van Alen T, Hackstein JHP, Henrissat B, Baker SE, Grigoriev IV, O'Malley MA. 2017. A parts list for fungal cellulosomes revealed by comparative genomics. Nat Microbiol 2:17087.
10. Katoh K, Standley DM. 2016. A simple method to control over-alignment in the MAFFT multiple sequence alignment program. Bioinformatics 32:1933-42.
11. Guindon S, Dufayard JF, Lefort V, Anisimova M, Hordijk W, Gascuel O. 2010. New algorithms and methods to estimate maximum-likelihood phylogenies: assessing the performance of PhyML 3.0. Syst Biol 59:307-21.
12. Le SQ, Gascuel O. 2008. An improved general amino acid replacement matrix. Mol Biol Evol 25:1307-20.
13. Lefort V, Longueville JE, Gascuel O. 2017. SMS: Smart Model Selection in PhyML. Mol Biol Evol 34:2422-2424.
14. Herivaux A, Lavin JL, de Bernonville TD, Vandeputte P, Bouchara JP, Gastebois A, Oguiza JA, Papon N. 2018. Progressive loss of hybrid histidine kinase genes

during the evolution of budding yeasts (Saccharomycotina). Curr Genet 64:841-851.
15. Sievers F, Wilm A, Dineen D, Gibson TJ, Karplus K, Li W, Lopez R, McWilliam H, Remmert M, Soding J, Thompson JD, Higgins DG. 2011. Fast, scalable generation of high-quality protein multiple sequence alignments using Clustal Omega. Mol Syst Biol 7:539.
16. Naranjo-Ortiz MA, Gabaldon T. 2019. Fungal evolution: diversity, taxonomy and phylogeny of the Fungi. Biol Rev Camb Philos Soc 94:2101-2137.
17. James TY, Stajich JE, Hittinger CT, Rokas A. 2020. Toward a fully resolved fungal tree of life. Annu Rev Microbiol 74:291-313.
18. Li Y, Steenwyk JL, Chang Y, Wang Y, James TY, Stajich JE, Spatafora JW, Groenewald M, Dunn CW, Hittinger CT, Shen XX, Rokas A. 2021. A genome-scale phylogeny of the kingdom Fungi. Curr Biol 31:1653-1665 e5.
