# Supplementary material for: Predicted Functional and Structural Diversity of Receiver Domains in Fungal Two-Component Regulatory Systems
Source: mSphere. 2021 Oct 6;6(5):e00722-21. doi: 10.1128/mSphere.00722-21 (PMC8510515; doi:10.1128/mSphere.00722-21)
Supplement: TEXT S2 [file msphere.00722-21-s0002.pdf]

List S1. Fungal Receiver Domain Sequences Used In This Study
(.fasta files are available upon request to bourret@med.unc.edu)

Rim15 Asc (n = 24)

>tr|Q4X019|AfumiRim15|1540-1654
DVLICEDHPVSRMVMERLFEKLRCRTITAVNGAEAMRYALSEVQFDIIMTEFKLPQVNGA
DLARMVRETRSANRHTPIIAVTGYLKDLPETHHFDALIQKPPTLTKLTEALCKFC

>tr|G1XBK9|AoligRim15|1529-1643
DILVCEDHPVSRMVMERMFEKLQCRTMSVETGPEAVRCAMSEVKFDLILTEYKLPQINGD
DLARMIKTTKNANSSTPIVCITGYLRDLKDPHHFDDLIEKPATPQSLTEVMERLC

>tr|J4W0C5|BbassRim15|1533-1647
DVLICEDHPVSRMVMEKLLEKLRCRTISVSNGSEAVRYSMSEIKFDVIFLEFRLPQINGA
DVARMIRETKNTNAHTPIVAITSYLKELQAPQYFDSLIEKPISSSKLGEVLRRLC

>tr|M7UQE6|BcineRim15|1518-1632
DVLICEDHPVSRMVMEKLLEKLRCRTVTVSNGADALRYAVSDIKFDIIMMEFKLPQLNGA
DVARMIRETKNANSQTPIVAITGYLKELQAPHQFDALIEKPPTASKLTEVLCRLC

>tr|C5GVM3|BdermRim15|1611-1725
DVLICEDHPVSKLVMERLFEKLRCRTITAVNGSEAMRYALSEVQFDIIMMEFKLPQLNGA
DVARMIRDTKSVNTHTPIICCTGYLKDLPETHHFDALIEKPPTLSKLTEALCKFC

>tr|N1JD50|BgramPpk18|1513-1627
DVLICEDHPVSRMVMEKLLEKLRCRSITVSNGAEAMRYAMSEIKFDIILMEFKLPQINGA
DVARMIRDTKNANSHTPIVAVTGYLKDLQAPHQFDALIEKPPTTLKLTEIMSHLC

>tr|A0A1D8PQP3|CaRim15|1806-1923
DVLYCEPISVIRHSVVKLLEKAGCIVVSVTDGEELIKRATSQVKFDLIFTGLKISKVDAI
DAVKLIKFTSGKNRNTPIIGITENKNKIDDDITTSSTFDYIIEPNLEAISKVCRILRS

>tr|Q6SLC1|CheteRim15|1530-1644
DVLICEDHPVSRLVMEKLLEKLRCRTLTVTNGSEAIRYAMGEVKFDVIMMEFRLPQVNGA
DVARMIRDTKNANTHTPIVAVTGYLKELQAPHYFDALIEKPPTVAKLTDTLSRLC

>tr|J3KL84|CimmiRim15|1531-1645
DVLICEDHPVSRLVMERLFEKLRCRTITAVNGTEAMRFAFSEVQFDIIMTEFKLPQINGA
DVSRMVRDTRSANTHTPIIAVTGYLKDLPETHHFDALVEKPPTLEKLVDVLSKFC

>tr|M1VZL9|CpurpRim15|1499-1613
DVLICEDHPVSRMVMEKLLEKLRCRTISVSNGSEAVRYAMSEIQFDVIFLEYKLPQISGA
DVARMIRETKNANSHTPIVAITAYLKELQAPHYFDSLIEKPISSSKLTEVLRSLC

>tr|Q6SLA5|FvertRim15|1520-1634
DVLICEDHPVSRMVMEKLLEKLRCRTISVPNGSEAVRYSMSEIKFDVIFLEYQLPQINGA
DVARMIRETKNANSHTPIVAITAYLKELQAPHYFDSLIEKPISSSKLTEVLRGLC

>tr|F0U745|HcapsPpk18|1606-1720
DVLICEDHPVSKLVMERLFEKLRCRTITAVNGSEAMRYALSEVQFDIIMMEFKLPQLNGA
DVARMIRDTKSVNTHTPIICCTGYLKDLPETHHFDALIEKPPTLSKLTETLHKFC

>tr|G0RDM8|HjecoRim15|1525-1639
DVLICEDHPVSRMVMEKLLEKLRCRTISVANGSEAVRYSMSEIKFDIIFLEYRLPQINGA
DVARMIRETKNANSHTPIVAITAYLKELQAPHYFDSLIEKPISSSKLTEVLRSLC

```
>tr|A0A014PB19|ManisRim15|1512-1626
DVLICEDHPVSRMVMEKLLEKLRCRTISVANGSEAVRYSMSEIQFDVIFLEYKLPQISGA
DVARMIRETKNANSHTPIVAITAYLKELQAPHYFDSLIEKPISSSKLIEVLRSLC

>tr|F9X076|MgramRim15|1544-1658
DVLVCEDHPVSRLVMERLLEKLRCRTIAVTNGTEAVRYAMSEVKFDIIMMEYKLPQVNGA
DVARMIRDTKNANSHTPIVAVTGYLKELHAPHHFDALVEKPPTKEKLEEVMGRLC

>tr|G4NCR0|MoryzRim15|1535-1649
DVLICEDHPVSRMIMEKLLEKLRCRTISASNGSEAVRYSMSDIKFDIIFMEFRLPQINGA
DVARMIRETKNSNSHTPIVAITAYLKELQAPHYFDSLIDKPISSSKLTEVLRNLC

>tr|Q7S2I4|NcrasStk12|1508-1622
DVLICEDHPVSRMVMEKLLEKLRCRTISASTGQEACRYALSDIKFDIIFMEFKLPVINGS
DVARMVRETKNKNHHTPIVAITAYLKELHAPHYFDSLIEKPISSSKLSEVLSTFC

>tr|C1FZN9|PbrasRim15|1621-1735
DVLICEDHPVSKLVMERLFEKLRCRTITAVNGSEAMRYALSEVQFDIIMIEFRLPQLNGA
DVARMIRDTKSVNTHTPIICCTGYLKDLPETHHFDALIEKPPTLSKLTEALCKFC

>tr|B6QJY3|PmarnRim15|1531-1645
DVLICEDHPVSRLVMERLFEKLRCRTITAINGSEAIRYALSEVQFDIIMTEVKLRQINGV
DVARMIRETRSANRHTPIIAVTGYLKDLPEAHHFNALIEKPPTLSKLTEALCKFC

>sp|P43565|SccereRim15|1636-1750
DVLVCEPIPIHRYRVTKDLENLGCTVVSVGAGDELVSRATSGVSFDLIMTALKLPKLGAI
DIVQLLKQTNGANSTTPIVAITNYFQEAATSRVFDDVLEKPVKLDELKKLVAKYA

>tr|U7PNT1|SscheRim15|1554-1668
DVLICEDHPVSRMVMEKLLEKLRCRTISAASGPEAVRYAMSDIKFDIIFMEYKLPQISGA
DVARMIRDTTNTNSQTPIVAITAYLKELQARQHFDSLIEKPISSSKLTEVLASLC

>tr|D5GNM8|TmelaRim15|1310-1424
DVLVCEDHPVSKLVMERLLEKLRCRTICVDNGAEAMRYAMGEVKFDIILMEFKLPQINGE
DVARMIRSSKNPNSNTPIVAVTGYLKDLSDPHHFTELIEKPATPVKLTDVLERNC

>jgi|Usnflo1|844576|UflorRim15
DVLICEDHPVSRMVMERLLEKLRCRTITVTNGPEAARYAMSEVQFDIIMMEFKLPQINGA
DVARMIRDTKSANTQTPIIAVTGYLKELPQTHHFDSLMEKPPTLEKLTEALCNLCAW

>jgi|Xylhe1|267709|XheveRim15
DVLICEDHPVSRMVMERLLEKLRCRTIAVPNGSEAMRYAMGEVKFDVIMMESKLPHINGA
DVARMIRDTKNVNSQTPIVAVTGYLKDLQAPHSFDALIEKPPTISKLTEVMSRLCQ
```

Rim15 non-Asc (n = 23)

>tr|A0A068S548|AcoryRim15A|2305-2420
DCLVADDNPINCKILETILTALGCRCVIVRNGAQAIRCAMGDDVQFDLIFMDIRMPIIDG
ETASRMIKSTQNINQQTPIIAVTAYERSAQHAGSFDDILLKPLNQSMVLQQLRQFY

>tr|A0A068RRN5|AcoryRim15B|2473-2587
NCLVVDDNPISCKILETILHTLNSRCVIARNGAQAIRIAMGDVRFDIIFMDIRMPIIDGE
AAARMIKSTNNSNRDTPIIAVTAYERTVQLAGAFDDILSKPVTKTVIYERLQQFC

>tr|A0A1Y2HIP0|CanguRim15|768-882
RVLVADDNPIACKILETILRRLGAQCFVVYNGAEAVRLATGSVVFDLIFLDAGMPYLDGE
RAAQMIRATDNVNRNTVIVGVTGYDPMQLNQRFFNAIYQKMITKNQVLELFDKFG

>tr|D6RMI0|CcineRim15|1432-1548
TCLLAEDNPITAKIIETLLVRLGCRCIVVSDGSEAISVAMGDIKFDCILMDLQMPVLDGE
GAARYIKSTNGKNTNTPIIAVSAYSGMDPSETSNVFDASLSKPLQKADLLNVFRQLG

>tr|J9VHK4|CneofRim15|2741-2856
DVLVAEDNPISQKILETLLTRVGCRCICVDDGPSALAAIMGSIRFDVIICDIHMPVVNGE
QVARMIRSTNNHNQLTPIIAATSYEQYINEEGTLFSAVLAKPVTKADLIRCLAKLG

>tr|B0CY06|LbicoRim15|1694-1810
TCLLAEDNPITAKIIETLLIRLGCRCVVVADGSEAISVAMGDIKFDCILMDLHMPVLDGE
GAARYIKSTNSKNTNTPIIAVSAYSGTDPNETSNVFSASLSKPLQKADLLAVMRQLG

>tr|S2J3V7|McircRim15A|2229-2344
NCLIADDNPISCKILETILQILQCRCVIVRNGAQAIRCAMGDKVQFDFIFMDIRMPIIDG
EAAARMIKSTNNINKNTPIIAVTAYERTLQLASIFDDTLCKPVTKEIVSRCIRHLS

>tr|S2JQM9|McircRim15B|2717-2831
ACLVADDNPISCKIIETILQMLHCRCVIVRNGAQAIRSAMSDVQYDIIFMDIRMPIIDGE
TAARMIKSTNNNNRETPIIAVTAYERTVQLAGAFDDILSKPVTKHVILQRLKQYC

>tr|F4RYX2|MlariRim15|3378-3497
DCLIAEDNPISSKVLETILTRFGCRCVVVPNGAEAISCAMGDVAFDVIFMDLMMPIIEGQ
DAARMIKSTQNVNAHTPIVAVTSFESYIIAPSDSEGTLFAALLGKPVGKKDVLDVMKRLG

>tr|K5W5F9|PchryRim15|1696-1813
TCLLAEDNPITAKIIETLLIRLGCRCVVVADGSEAISVAMGDIKFDCILMDLHMPILDGE
GATRYIKTTNNKNASTPIIAVSAYGGSEPPTEQNNLFAAYLQKPVQKADLLAVMRQLG

>tr|A0A1Y1VIZ6|PfinnRim15|2892-3010
DILIVEDNPISSKVLESMLTKLNCRCVVVTNGADAIRCAMGDVKFDIIFMAIKMPLERYS
FDGPIAARMIKSVTNINQNTPIIGVTAYEQTYNLTQEFDDILNKPITKDDLIQELEAFI

>tr|H6QSI2|PgramRim15|3476-3593
DCLIAEDNPISSKVLETILIRLGCRCVVVPNGEDAISCAMGDIAFDVIFMDLMMPLIEGQ
DAARMIKSTQNPNALTPIVAVTSFFENYSCSEQGTLFAGLLIKPVNKKDVLGMLKKLG

>tr|A0A067NYX1|PostrRim15|1716-1832
TCLLAEDNPITAKIIETLLIRLGCRCVVVADGSEAISVAMGDIKFDVILMDLQMPVLDGE
GAARYIKSTNGRNANTPIIAVSAYSGIDMNEANNVFVASLAKPLQKADLLNVMRQIG

>tr|B8P313|PplacRim15|1522-1638
TCLLAEDNPITAKILETLLIRLGCRCVVVADGSEAISVAMGDIKFDCILMDLHMPILDGE
GAARYIKNTSNKNASTPIIAVSAYSGSDTLEAGNLFAGYISKPVQKADLISVMRQLG

>tr|A0A075B0I5|RalloRim15|857-969
ECLVVDSNPISVRILETLLGRLNCTCLNAIDGIDALRILLDKKFDIVFINIELPRLNGDQ
VARLIKSTRNINREVPILATTAYGVASDVEYFDSFLMKPLTKEKIEDELSRFL

>tr|I1BYJ6|RdeleRim15A|1606-1721
DCLIADDNPISCKILETILKSLHCRCVIVRNGAQAIRCAMGDKVLFDFVFMDIRMPIIDG
EAAARMIKSTNNINRTTPIIAFTAYERTFQLTKIFDDVISKPVTREAIVRCIKQFR

>tr|I1BXF8|RdeleRim15B|2555-2669
SCLIADDNPISCKIIETILQMLHCHCVIVRNGAQAIRSAMSDVRYDIIFLDIRMPIIDGE
SAARMIKSTNNSNRDTPIIAVTAYERTVQLAGAFDDILSKPVTKDIISQRLKQFC

>tr|I1BJU2|RdeleRim15C|2167-2281
ACLVADDNPISCKIIETILQTLHCRCVIVRNGAQAIRSAMSNVQYDIIFLDIRMPIIDGE
TAARMIKSTNNKNRDTPIIAVTAYERTVQLAGAFDDILSKPVTKDIISQKLKQFC

>jgi|Synplu1|207836|SplumRim15
DVLIADDNPVACKILETVLGKLNCRCVVVRNGAEAIRCAMGKVVFDIIFMDVVMPIVDGE
AAARMIKSTNNANRQTPIIAVTAYEHPIHLARVFDEVLVKPVDKATLQHRLRYFCQ

>jgi|Sporo1|33825|SroseRim15
PATIDCLVAGRNPIVTKVLETMLQRLGCRVVVVPNGAEAILAAGGIPFDVLFLDLKMAVV
DGEKAARMIKSTVNPSAQAPIVAVCSHPAAIDDAAGTLFTATLSKPIMKADLLHVLAYLG
F

>tr|K1VU09|TasahRim15|
SLVVLVAEDNPISQMTLLSRLGCRCVCVQDGPEALAAAMGSISGENVAFNDQHQPEHTHL
LCDTTQTVLKGRAPSVPRQSRLFT

>tr|A0A0D1CAE8|UmaydRim15|3879-3996
ACLIAEDNPIALRMLETMLVKLGCRCTAVRDGAEAVRLAMGDHKFAVMFIDVTLPIVGGE
DVTRMVKSTRNVNSTTPIVALASFDRGEPIDAAGSLFDAVLAKPLEKMDVCAILSQLG

>jgi|Umbra1|236075|UramaRim15
DCLIADDNPISCKILETILYTLNCRCVVVRNGAQAIRCAMGGVKFDIIFMDIRMPIIDGE
AAARMIKSTINMNSTTPIIAVTAYEHTVQLAGAFDDILNKPVTTQIILNRLKQFCD

Skn7 (n = 55)

>tr|A0A068RNW3|AcorySkn7A|385-499
HVLLVDDDMIFRRLSTKLLQVAGCTIDVASDGLEAITKLGSRSYDIVLMDIMMPKLDGIS
ATRNIRQYDTWTPIISMTSNTTDRDVQEYILSGMTDVLPKPLDSGTLRKLLERYC

>tr|A0A068SGS5|AcorySkn7B|407-521
RVLLVDDDSIFRRLSTRLLEMAGCTIEVAVDGVEAIDKLGTDHYDIVLMDIMMPNLDGIS
ATRNIRQYDTWTPIISMTANTTDRDVREYIMSGMTDVLPKPLDQSTLCKLLERYC

>tr|A0A068SC36|AcorySkn7C|465-579
HVLLVDDDRVCRDLSGKLLQLVGCTIDLAKDGVEALHKLTVKKYDLVLMDIMMPNLDGIS
ATRNIRQYDNFTPIVSMTSNFTDKDIMLYAGSGMTDILPKPFTADTLYSILERYC

>tr|A0A068S490|AcorySkn7D|419-533
RVLLVDDDSVYRDLSGKLLNMIGCTIDLAKDGVEALHKMNAEKYDLILMDIVMPNLDGVS
ATRNIRQYDMLTPIISMTSNFTDNDIMQYVGSGMTDILPKPFSKRTLYSMLEKYC

>tr|A4D9Q8|AfumiSkn7|366-480
HILLVEDDATCRQIGGKFLYSFSCVIDTAFDGLEAVNKIQDGSKYDLILMDIIMPNLDGV
SACHLIRQFDRTPIIAMTSNIRSDDIQLYFQHGMDDVLPKPFTRKSLLDMLEKHL

>tr|G1XIP1|AoligSkn7|317-437
KILLVEDDPTCRKIGLKFLEAVGCLPFYACDGLEGIRKFQDLNGPHYDLVLMDIVMPHLD
GASACGEIRKGRPDMKDPPVIAMTSNIRTTDINLYFNSGMIDVLPKPFTRDGLLQTLQKW
L

>tr|J5JXW8|BbassSkn7|359-476
HVFLVEDDPTCAKIGIKFLKSMGCEVEHAPDGAEAFNRMNAVGRDHFDLIFMDIIMPKLD
GVSASMYIRQQSPSTPIIAMTSNIRPDEVNGYFEHGMNGVLAKPFTKEGMLKSVKTHL

>tr|M7TZI2|BcineSkn7|372-489
RILLVEDDQVCARIGTKFLESFECGVDIARDGLEAVSKINLGAENHFDLILMDIIMPHLD
GVSATVCIREIRANIPIIAMTSNIRTDDIEMYFRYGMNDVLPKPFTKEGMMKALEKHL

>tr|C5GW12|BdermSkn7|371-485
RILLVEDDQTCRQVGGKFLLSFCCTVDYALDGLEAVNKIQEGSKYDIILMDIIMPNLDGV
SACSVIRRVDTTPIIAMTSNIRSSDIELYFQHGMNDVLPKPFTRQSLLTVLERHL

>tr|N1JCY5|BgramSkn7|337-453
SILLVEDDKVCARIGTKFLQSFDCAVDTAQDGLEAVNKVNTDQFAFDLILMDIIMPRLDG
VSASVCIKELRPNIPIIAMTSNIRADDIEMYFSHGMNGVLPKPFTKEGMLRALEKHL

>sp|Q5A4X5|CaSkn7|425-539
HVLLVEDDNVCIQLCRKFLVKYGCSVTVVTDGLNAISTVEHTKYDLVLMDIVMPNLDGAT
ATSVIRSFDTKTPIIAMTGNIEDNDLVTYLQNGMSDILAKPFTKDDLYAILSKHL

>tr|A8PG33|CcineSkn7|550-664
RVLLVDDDAVSRKLSSKFLQVFGCTTDVAVDGVGAVNKMNLEKYDLVLMDIVMPKMDGIS
ATSIIRQFDKGTPIISMTSNSKPNEIMTYYSSGMNDILPKPFTKQGLLDMLEKHL

>tr|A0A137PIJ3|CcoroSkn7|374-488
NVLLVDDDPICRDMYSKCLQKLGCSFDIAADGAEAVNKMHTRKYDMVLMDIWMPQMDGLS
ATTIIRQFDQQTPIIAITGDYRETDKGFYLGQGINEVLGKPFKIDQLGECMQKHW

>tr|Q6SLC2|CheteSkn7|353-467
QILLVEDDPTCRRIGSKFLYAFHCSIDSALDGLEAVNKMNAGSKYDLVLMDIIMPNLDGV
SACHLIRQFDTTPIVAMTSNIRSDDISMYFQHGMNDVLPKPFTKEGLLHMLEKHL

>tr|J3KJU1|CimmiSkn7|369-483
HILLVEDDQTCRQIGGKFLYSFSCVIDTAFDGLEAVNKIQSGSKYDLILMDIIMPNLDGV
SACSVIRRFDATPIIAMTSNIRSSDIELYFQNGMNDVLPKPFTRQSLLNMLEKHL

>tr|J9VQU1|CneofSkn7|703-818
RILVVEDDIVYRTLSKKFLQKFGCETETVENAQGAVDKMNGTKYDLVLMDIFFGPNMDGR
KATSLIRQFNNYTPIISMTSNAQPQDVDSYYQSGMNDILAKPFTKNHLFTILDKHL

>tr|M1W0Q5|CpurpSkn7|371-488
RVLLVEDDPTCAKIGIKFLNSMGCEVDHAQNGVDAYTRVSSFSRDHFDLMFMDIIMPRLD
GVSATMYIRQHCPSTPIIAMTSNIRPDEVNEYFEHGMNGVLAKPFTKEGMLKSIKTNL

>tr|W7LYD8|FvertSkn7|347-464
RIFLVEDDPTCAKIGIKFLKSMGCEVEHAQNGADAYSRITSVARDHFDMIFMDIIMPKLD
GVSTTMYIRQDCPAIPIVAMTSNIRSDEVHCYFEHGMNGVLAKPFTKSGMQKIVENHL

>tr|A0A138ZXL0|GprolSkn7|340-453
HVLLVDDDPVCRMLSARFLQLFGCIYDLASDGSEALEYIDMGRKYDIVLMDIVMPNIDGI
MATTHIRQFDQITPIISMTSSTTERDCIHYFSSGMTDILAKPFNRDSLLKVLET

>tr|F0U7T1|HcapsSrra|370-484
RILLVEDDQTCRQVGGKFLLSFCCTVDYALDGLEAVNKIQEGSKYDLILMDIIMPNLDGV
SACSVIRRVDTTPIIAMTSNIRSSDIELYFQHGMNDVLPKPFTRQSLLTVLERHL

>tr|G0RI53|HjecoSkn7|360-477
VVFLVEDDGTCAKIGIKFLKSMGCEVEHAINGVEAYNRINAVGRDHFDLIFMDIIMPRLD
GVSATMYIRQHCPTTPIIAMTSNIRPDEVNGYFEHGMNGVLAKPFTKEGMLKSVKTNL

>tr|B0DFY8|LbicoSkn7|247-361
RVLLVDDDAVTRTLSKKFLQVFGCTTDIAVDGVGAVTKMNLEKYDLVLMDIVMPKLDGVA
ATSLIRKFDQGTPIISMTSNSKPNEIMTYYNHGMNDILPKPFTKEGLLDMLEKHL

>tr|A0A0A1V8T5|ManisSkn7|351-468
QVLLVEDDQTCAKIGIKFLNSMGCEVEHALNGADAYTRVSDVGRDHFDLMFMDIIMPRLD
GVSATAYIRQHCPTTPIIAMTSNIRPDEVIRYFEHGMNGVLAKPFTKEGMLKSVRTHL

>tr|S2JAA9|McircSkn7|338-452
RVLLVDDDSLFRRLSTRLLQIAGCTIDVAVDGMEAVRKLGTGKYDLVLMDIMMPKLDGMS
ATRNIRQYDTWTPIISMTSNTTDQDIQQYFMSGMTDVLPKPFNQGSLGSLLERYC

>jgi|Morel2|706983|MelonSkn7A
KVLLVEDDDTCRRLSSRLLQIFGCPFDVAEDGVAAVGKMSHQKYDIVLMDIMMPKLDGVS
ATTQIRQFDAMTPIISMTSNTTANDIMTYFANGMNDILPKPFSKDSLLSMLEKHCQ

>jgi|Morel2|498468|MelonSkn7B
KVLLVDDDDTCRRLSSRLLQIFGCPFDVAEDGMAAVGKMSHQKYDIVLMDIVMPKLDGVS
ATTQIRQFDAMTPIISMTSNTTNNDIMTYFANGMNDILAKPFSKAGLLNMLEKHC

>tr|F9XE66|MgramSkn7|379-494
KILLVEDDQTCRRIGGKFLYAFQTSIDSALDGLEAVNKLNNGNKYDLVLMDIIMPNLDGV
SATHLVRQFDNTTPIIAMTSNIRSDDISMYFQHGMNDVLPKPFTKEGLLSMLEKHL

>tr|F4RQL4|MlariSkn7|317-431
TVLLVEDDQIYRSLSGRILEFIGCKMVTCNDGIEAVQVMAQQQFDLVFMDIYMPFMDGIL
ATSLIRQFDLFTPIISMTSNFTPTEINKYLGIGMNDCLPKPFTKEKMLKMLEKHL

>tr|G4N7Y2|MoryzSkn7|363-479
CILLVEDDKVCQRIGSKFLTHFGCDVETAMNGFDAVSKFSSNPNRYSLIFMDIIMPEMDG
VSATSSIRQRGAVIPIIAMTSNIRQEDITTYFEFGMNDVLAKPFTKEGMVRIVKKHL

>tr|Q7S471|NcrasPrr1|362-480
RILLVEDDKTCARIGAKFLHVLECSVDIAKDGLEAVEKINNPENNEGFDLIFMDIIMPNL
DGVSATAMIRMVTARVPIIAMTSNIRQEDIQTYFQYGMNDVLAKPFTKDSMIRVLRKHL

>tr|C1GBB4|PbrasSkn7|345-459
RILLVEDDQTCRQVGGKFLLSFCCTVDYALDGLEAVHKIQEGSKYDLILMDIIMPNLDGV
SACSVIRRVDSTPIIAMTSNIRSSDIELYFHHGMNDVLPKPFTRQSLLTVLERHL

>tr|K5W707|PchrySkn7|636-750
RVLLVDDDAISRKLSSKFLQVFGCTIDVAVDGDVAVNKMNLEKYDLVLMDIVMPRLDGVS
ATSLIRQFDHMTPIISMTSNSKPDDITKYYSSGMNDVLPKPFTKDGLLEMLERHL

>tr|A0A1Y1V9B9|PfinnSkn7|532-646
RVLLVEDDAICRALSSKLLQVFGCKFDIAVDGQSAIERMGDRKYDIVLMDIVMPKIDGVT
ATNRIRQFDQLTPIISMTSNTTENDCITYLRNGMNDVLPKPFNKNGLLNMLERYC

>tr|E3K3B6|PgramSkn7|410-529
RVLIVEDDQICRRISSTILELMGCRIEFACDGLNAVSRMKTQIDANDPFDLVLMDIFMPN
MDGLSATSLIRKFDLKTPIISMTSNFQPVDVLKYINIGMNDCLPKPFTKEGMIIMLQKHL

>tr|B6QE20|PmarnSkn7|361-475
NILLVEDDDTCRQIGGKFLGSFQCQIDNAYDGLQAVTKVQSGKRYDLILMDIIMPNLDGV
SACHLIRQFDRTPIIAMTSNIRKDDIELYFQHGMDDVLPKPFTRKSLLDMLEKHL

>jgi|PleosPC15_1|153194|PostrSkn7
RVLLVDDDAVSRKLSSKFLQVFGCTIDVAMDGVAAVNKMNLEKYDLVLMDIVMPKLDGVS
ATSLIRKFDHMTPIISMTSNSKPNDIMTYYSSGMNDILPKPFTKEGLLDMLEKHLM

>tr|B8PEA7|PplacSkn7|425-539
RVLLVDDDAVSRRLSSKFLQVFGCTIDVAVDGVGAVNKMNLEKYDLVLMDIVMPKLDGVS
ATSLIRQFDHMTPIISMTSNSKPNEIIKYYSSGMNDILPKPFTKDGLLDMLEKHL

>tr|A0A4P9YPY3|RalloSkn7A|417-531
KVLLVEDDAVCRTIGSKLLQVFGCTFDIASDGVDAIQKMNIQRYDIVLMDIVMPNLDGVS
ATCRIREFDKQTPIISMTSNVRRDDCVKYLANGMNDILPKPFSRESLLAMLDRYC

>tr|A0A075AW50|RalloSkn7B|455-569
KVLLVEDDAVCRTIGSKLLQVFGCTFDIASDGVDAIQKMNIQRYDIVLMDIVMPNLDGVS
ATCRIREFDKQTPIISMTSNVRRDDCVKYLANGMNDILPKPFSRESLLAMLDRYC

>tr|I1CQL2|RdeleSkn7A|371-485
RILLVDDDSVYRDLSERLLHVFGCTIDLAKDGVEALKKMGLERYDLILMDIVMPKMDGIS
ATRSIRQYDALTPIISMTSNFTDNDIMQYIGSGMTDILPKPFSKRTLYQMLDKYC

>tr|I1CEL8|RdeleSkn7B|340-444
RVLLVDDDSIFRRLSTKLLQMAGCTIDVAVDGEEAVRKLGTMKYDLVLMDIMMPKLDGMS
ATRNIRQYDTLTPIISMTSNTTDQDIQQYILSGMTDVLPKPFNQV

>jgi|Gloin1|34040|RirreSkn7A
KILFVDDEIFVREGSSQWFQAYGCQFDVAVDGITAVNKMQIHQYDIIFMDIVLQSEFDGL
SVTQQIRRFNNEIPIIGMPPPHSSSNSNNNQSANNSTNADYVNYLRSGMNDLLPKPFTRQ
ELYDKIDKFCS

>jgi|Gloin1|14063|RirreSkn7B
KVLLVDDDAVYQNIGSKFLQVFGCAIDIAVDGISAVNKMNFEKYDLVLMVIL

>sp|P38889|ScereSkn7|378-492
HVLLVEDDAVSIQLCSKFLRKYGCTVQVVSDGLSAISTLEKYRYDLVLMDIVMPNLDGAT
ATSIVRSFDNETPIIAMTGNIMNQDLITYLQHGMNDILAKPFTRDDLHSILIRYL

>jgi|Synplu1|656966|SplumSkn7
KVLLVEDDIVCRRLSGKILQVFGCTFDEAEDGLTAVNKMNISKYDLVLMDIMMPHLDGMS
ATAQIRQFDRYTPIVSLTANFTQDARIVYMSCGMNDVLPKPFSKDTLLSVVEKYCV

>sp|O14283|SpombPrr1|369-483
RILLVEDDELSRRMTIKFLTSFDCQVDVAVDGIGAVNKANAGGFDLILMDFILPNLDGLS
VTCLIRQYDHNTPILAITSNISMNDAVTYFNHGVTDLLVKPFTKLTLLQLLKKQL

>jgi|Sporo1|33220|SroseSkn7
RVLLVEDDAVCRKLSSKFLEVFGCDIDVAVDGVSAVNKMKMQKYDLVLMDIVMPNLDGVS
ATSMIRQFDGTTPIVSMTSNSGPNDVMTYFSHGMTDVLPKPFTKQNLRGMLDRHLP

>tr|U7Q424|SscheSkn7|344-460
KILLVEDDKLCARIGAKFLSQIDCSVDIARDGAEAVAKVNQDPERYDLIFMDIIMPVFDG
VSATACIRIAAPRVPIVAMTSNIRSEDIATYFHWGMNDVLAKPFTKEGMVRILRKLL

>tr|K1VBS4|TasahSkn7|605-720
RILVVEDDVVYRQLSSKFLEKFGCIVETVENAQQGIERMNTAKFDLVLMDIFFGPSMDGR
KATSLIRQFDMYTPIISMTSNVQTKDVDSYLQSGMNDVLAKPFTKHGLFCILDKHL

>tr|D5GD35|TmelaSkn7|357-471
RVLLVEDDPTCARIGSKFLQTAECSVDMASNGLIAVNKLNSGKYDLVLMDIVMPQLDGVS
AASLVRQFDSTTPIIAMTSNIRRDDINMYFNHGMNDVLPKPFTKEGLLNMLEKYL

>jgi|Usnflo1|815206|UflorSkn7
QILLVEDDPTCRRIGSKFLYSFRCAIDSAFDGLEAVNKMNAGSKYDLVLMDIIMPNLDGV
SATHLIRQFDHTPIIAMTSNIRSDDISMYFQHGMNDVLPKPFTKEGLLNMLEKHLG

>tr|A0A0D1E2D8|UmaydSkn7|366-480
RVLLVDDDQVCRRLSSKFLQVFGCSIDYAVDGMTAVNKMNQEKYDLVLMDIVMPNLDGIS
ATSLIRQFDSNTPIISMTSNSGPSELINYMSSGMTDILPKPFTKEGLLNMLEKHL

>jgi|Umbra1|247899|UramaSkn7A
RVLLVDDDSVCRNLSSKLLQVFGCTFDVATDGVEALKKLGLEKYDLVLMDVVMPNLDGVT
ATRNIRQYDTLTPIISMTSNTTDSDIMEYFGSGMNDVLPKPFSTNGLLDMVVKYCA

>jgi|Umbra1|211819|UramaSkn7B
RVLLVDDDSVFRNLSSKLLQVFGCTFDVAADGVEAMHKLGLEKYDIVLMDIVMPNLDGVS
ATRNIRQYDTMTPIISMTSNTTDSDILEYIGSGMTDVLPKPFSKQSLYDMLDKYCA

>jgi|Xylhe1|161803|XheveSkn7
QILLVEDDPTCRRIGSKFLYSFRCAIDSALDGLEAVNKMNAGSKYDLVLMDIIMPNLDGV
SACHLIRQFDSTPIIAMTSNIRSDDISMYFQHGMNDVLPKPFTKDGLLHVLEKHLG

Srr1 (n = 19)

>sp|Q59M56|CaSrr1|155-273
SFLIVDDNIINLKILNRILLKLFPKCHIVQIQDSKLVKDLLHKQSFDSIFIDIEMPDVNG
IDIAQFVRQDTKFDNMGMVAVTTRNSTQDLELFKQCGIDFTFHKPLNYSLDFMANSIDD

>|Cepal1_1|32007|CalbiSrr1|185-307
KFLLIDDNPINLKILSKVLIKLYPNSTIHPTSNPKEVLSLVESNKYDVLFLDIEMPEMSG
IDIAKIIRTKQAEVKKYEKLSLIAVTSRSSMENMKEYEELGIDLTLPKPLTRDYNSLISK
IDK

>tr|A0A0L0P6S4|CauriSrr1|46-165
HFLLVDDNIIYLRIFARILRKLFPQALVRAIQDLLVLELTEELFLRFDCVFLDIDMPRVT
GIDVATQLRSFLSLDHVGLVAVTTKALLSDMELYESIGFDHTFPKPLNVAHGDLLEKIEQ

>sp|B9WLY5|CdublSrr1|158-276
SFLIVDDNIINLKILNRILLKLFPKCHIVQIQDSKLVKDILHKQPFDSIFIDIEMPDVNG
IDIAQFVRQDSKFDDMGMVAVTTRNSTQDLELFKQCGIDCTFHKPLNYSLDFMANSIDD

>|Cepfr1_1|473726|CfragSrr1|216-354
RFLLVDDNPINLKVLSKILSKLYPNAIIKATPDPAEVLNILSDPKTLNEERIDILFLDIE
MPEISGIDIAKKIRHRTSPEPADNTNELRSNSFDKLSLIAVTSRSSTENLKEYENIGFDL
TLPKPLSRDYHTLINRINN

>sp|C4Y489|ClusiSrr1|62-181
SFLLVDDNEINLRIFRRMLLKLFPNASVRTVQESASVEISHRTLSHYHLIFLDIEMPIVT
GTEIASKVRSSRELDQVGLIAVTTKYLCADLELYEKLGFDFTFRKPVEYPTNYILQKIEQ

>tr|H8X666|CorthSrr1|177-295
KFLIVDDNVINLKILNRILLKLYPKASIVQVLDSTLVEEMVFKQSFDSIFIDIEMPEVTG
LDIARIVRNDSQFDTTSLIAVTTRNAPQDLQLFKQVGIDYTFGKPLNYKLDFMAETIDG

>|Canpa1|3218|CparaSrr1|178-296
KFLIVDDNIINLKILNRILLKLYPKAAIIQISDSTKVEQIVSEQSFDSIFIDIEMPHVSG
IDIAQFVRKDPQFDTTSLIAVTTRNTPQDLQLFKQVGIDYTFGKPLNYKLDFMAETIDG

>tr|A0A1E4SKR0|CtanzSrr1|14-132
SFLLVDDNAINLRILERVLNRMYPNSHITKIQDLTKVQPVIAHQAFDMVFLDIEMPCITG
VDLARHMRSQRKYDSWGIVAVTTRNTASDVAIYKSAGIDFTFPKPLATTHDHMMDTINY

>tr|G3B442|CtenuSrr1|94-212
KFLIVDDNLINLKILYKILNKLFPKALIVKLNNSSTVLKLVEQEKFDLIFLDIEMPPING
IEISKRIRSTKKFNRMGLIAVTTRSTEKDMKIYKKIGIDFTFKKPLNCNLNLILNNIEI

>sp|C5M3F1|CtropSrr1|138-256
RFLIVDDNIINLKILNRVLLKLYPRSNIVQVQDSKLVNEILQRQHFDSVFLDIEMPDITG
IDIAKFIRSDERFNQMAVIAVTTRNSAKDLQQYKECGIDHTFHKPLNYSLDLIGGSIDD

>sp|Q6BGW4|DhansSrr1|118-236
NFLLVDDNFINLKILERVLLKLYPNCTIVKTQDSTKIMALLHSQTFDVAFLDIEMPGLTG
IELAKMIRMEDKLNQVGIIAVTTKSLPCDKIIYEQAGIDHTFAKPLNYSFDHIITCIEK

>tr|A0A1E4RMU5|HburtSrr1|109-227
NFLLVDDNVINLKILAKFLNKLYPNSQCVQIQDSTKVMKTLEKQAFDLVFLDIEMPILTG
TDLASLIRLNHSNDGLGLIAVTTRHYIEDLKLYSKVGIDFTFAKPLNYSHNYMMSCIDQ

```
>sp|A5E0V1|LelonSrr1|209-327
KFLLVDDNLINLKILNRILLKLYPKAQITQVLDSTKVAKLVEENEYDAVFIDIEMPVVNG
VQIAQFIRSDVSKDDLTVIAVTTRNSKEDLALFEKTGIDYTFGKPLNYKLDFMANVIDE

>tr|A0A1A0HC25|MbicuSrr1|22-139
KFLIVDDNEINLRIFRRVLKRLFPNSAIDMMQDSSQVDTAKLLLYQIVFLDIEMPHVTGV
DIAKTVRLDPALHRLGLVAVTTKSAAADLETYDACGFDFTIPKPIYKGYNGIFRGIEQ

>|Metfru2|1254|MfrucSrr1|58-176
KFLVVDDNEINLRIFRRVLKKLFPYSLIDTIQDSTQVDPCALSQYKIVFLDIEMPQVTGV
DIANAVRSRTALNNLGLVAVTTKSTPPDMKLYEKCGFDMTIPKPVHRGYSGILRDIEQV

>sp|A5DLE2|MgullSrr1|90-209
RFLLVDDNSINLKILTKILLRLYPRAHIVELCDSTSVLAYLASTPPFDCVFLDIEMPVVS
GTELAYRIRQSPKLCRLPLIAVTTRTQEEDLAQYKDVGIDWTFGKPFNYPYRVVLDVVDN

>tr|G3AHH5|SpassSrr1|152-270
KFLIVDDNIINLKILNRILKKIYPRAEVTQVQDSTKVKPMLETATFDVVFLDIEMPKVNG
IELAQHMRYNSKFDKWGVIAVTTLTSARDLRIFKSCGIDYTFDKPMKVGLNEIGRIIDE

>sp|A3LP85|SstipSrr1|163-281
NFLIVDDNIINIKILERILFKLYPNCNIKKLQDPTKVANAIKTHKFDVVFLDIEMPEITG
VDISREMRQQPVFNSVGIIAVTTRTMAHDLLVYETVGIDYTFAKPLTYNYDFVMDRIDE
```

Ssk1 (n = 48)

>tr|A0A068RZE5|AcoryMcs4|1033-1201
NVLLVEDNMINIAILSTWMKKHKIGCTVANNGQEAVERWQQGGFHLVLMDLQLPVMSGIE
ATKKIRAVEKQQKIGVLPMNTSSSTTTDNGGEKASGTLPSLTTSSSTTTASDNPDELAPS
TFRSPVIIVALTASELESDRHAALAAGCNDFLTKPISLEWLEKKIIEWG

>tr|A0A068S9K8|AcorySsk1B|984-1131
NVLIVEDNMINQAILSTWMKKHKIKCSVASNGQEAVERWKGGGFHLVLMDIQLPVMDGIT
ATKTIRSIEKEQRIGVLPAEQDTASSSSLQQVPEDTQVSSFQSPVIIVALTASAKDSDRH
AALAAGCNDFLTKPISLEWLEKKIIEWG

>tr|Q4WUG7|AfumiSskA|558-719
NVLIVEDNIINQKLLEAFMKRLSVRWKCAANGEEAVRKWREGGFHLVLMDIQLPVMNGLD
ATKEIRRLERLNGIGVFTKTASGRSSASSLSPEVNQASSEVSLSEEDTLHDLSLFKSPVI
IVALTASSLQSDRHEALAAGCNDFLTKPVGFPWLEQKVTEWG

>tr|A0A6G1LZ59|AoligSsk1|793-939
NVLIVEDNIINLRLLEAFMKRLKVRWATAMNGREAVTKWRTGGFQLVLMDIQLPIMNGLD
ATKEIRRLERLNGIGSFSSSPSGPEDELSSADKLDKSLVFKGSVIIVALTASSLRSDRDE
ALAVGCNDFLTKPVNFTFLERKVMEWG

>tr|J4KQ56|BbassMcs4|523-673
SILIVEDNPINLKLLEAFVKRLKVRWQTATNGREAVKKWRTGGFHLVLMDIQLPVMNGLE
ATREIRRLERVNSIGVFSSVPTTPQEELALELNDKDRLENRHHFKGPVIIVALTASSLQS
DRHEALAAGCNDFLTKPVNFVWLERKVTEWG

>tr|M7UTT6|BcineSsk1|617-771
NVLIVEDNIINLKLLEAFMKRLKVRWQTAMNGREAVNKWRGGGFHLVLMDIQLPIMSGLE
ATKEIRRLERLNSIGVFSSSASSSAPSEKMLAEEEPEEDDKLPSTVLFKSPVIIVALTAS
SLQSDRHEALAAGCNDFLTKPVNFVWLERKVMEWG

>tr|C5GIQ5|BdermSsk1|642-810
NVLIVEDNIINLRLLEAFMKRLKVRWSTAMNGREAVNKWRAGGFHLVLMDIQLPVLSGLE
ATKEIRRYERLNNIGVFSRTVSGPAVKGSSSSSSPSSPVEGRHSRCPEILRPEDKLENPE
AFNSPVIIVALTASSLQSDRNEALAAGCNDFLTKPVNMVWLEQKVTEWG

>tr|N1JCZ6|BgramSsk1|302-453
NVLIVEDNFINLKLLEAFMKRLKVRWQTAINGKIAVDKWKTGGFHLVLMDIQLPVMSGLD
ATREIRRLERLNSIGVFNSSASSTAPQDIVDEAEGHDKLPSDALFKSPVIIVALTASSLQ
SDRHEALAAGCNDFLTKPVNFLWLERKVMEWG

>sp|Q5AKU6|CaSsk1|507-653
SVLVVEDNAINQAILGAFLRKRKIHYQIAKNGQEAIDKWKKGGFHLVLMDIQLPVKSGIE
ATKEIRHLEKLNRIGVFHENEIGKNVIINEEDRLTSNTFRSPVIIVALTASSNSSVDKTN
ALTAGCNDYLTKPVNLVWLQNKITEWG

>tr|A0A1Y2HGB6|CanguSsk1|2-169
RVLIVEDNPINLRILSAFLTKRNIQWAAAKDGAEAVRKFASCPPRTGLEFHLVLMDLQLP
VMDGLEATRKIRQVEDAWPPAALMAQSQQQQLRSVTPELLVDEGGLLHSVSIAPYHADLA
PSFRSIVVALTASNSSDDRKAAMAAGCNDFISKPINLRWLQAKIMEWG

>tr|A8PGR8|CcineSsk1|820-971
SVLIVDDNPINQTILSTFMKRKRISYDIANNGEEAVTKWRVGNFHLILMDIQMPIMDGIQ

ATKEIRRMEKANALAGYPPLTPNGEGQRTPSDTSSVASRATASPYRSSVIIVALTASSLQ
SDRDAALAAGCNDFLTKPVSLLWLNNKIIEWG

>tr|D9ZKR9|CheteSsk1|534-691
NVLIVEDNIINLRVLGAFMQRLKVRWQRAMNGKEAVTKWKAGGFHLVLMDIQLPVMNGLE
ATKEIRRLERVNGIGVFSSGSSEAPNTRLGSDSKSQDELREDDKLGDKGMFKSPVIIVAL
TASSLQSDRHEALAAGCNDFLTKPVNFVWLERKVKEWG

>tr|A0A0D8JWH7|CimmiSsk1|556-714
NVLIVEDNTINLKLLEAFMKRLKVRWQTAMNGREAVNKWRGGGFHLVLMDIQLPVMNGLE
ATREIRRLERVNNIGVFSKGVAATSPHAMVDTKGNDVFPPKPEDVLDKKSSVKSPVIIVA
LTASSLQSDRDEALAAGCNDFLTKPVNMIWLEKKVTEWG

>tr|J9VL35|CneofSsk1|994-1141
NVLIVEDNPINQNILSMFLRKKKIKNSSAKDGAEAVEKWRTGGFHLILMDIQLPVMDGIA
ATKEIRRLERHNNIGVFPSTPAAELPRGQNVADSPPPSSPFRSSVIIVALTASSLQSDRV
AALAAGCNDFLTKPVSLKWLDKKIVEWG

>tr|M1W6S0|CpurpMcs4|743-894
NVLIVEDNPINLKLLEAFVKKLKVRWQTAMNGRDAVDRWRTGGFHLVLMDIQLPVMNGLD
ATREIRRLERVNSIGVFSSTPDNNPDGNDESDLKEQDRLQNMSLFKSPVIIVALTASSLQ
SDRHEALAAGCNDFLTKPVNFVWLERKVMEWG

>tr|Q6SLA6|FvertSsk1|584-735
NVLIVEDNPINLKLLEAFVKRLKVRWSTAMNGRDAVKKWRTGGFHLVLMDIQLPVMNGLE
ATREIRRLERVNSIGVFSSSPDGVVTTENPDELDDKDRLQNPSLFKSPVIIVALTASSLQ
SDRHEALAAGCNDFLTKPVNFVWLERKVMEWG

>tr|F0UM88|HcapsSsk1|570-738
NVLIVEDNIINLRLLEAFMKRLKVRWSTAMNGREAVNKWRAGGFHLVLMDIQLPVLSGLE
ATKEIRRYERLNNIGVFSRTVSGQSAKGSTSSSTPSSPVEGKDNRCHETLRPEDKLENPE
AFKSPVIIVALTASSLQSDRNEALAAGCNDFLTKPVNIVWLEQKVTEWG

>tr|G0RFR5|HjecoSsk1|251-403
SVLIVEDNPINLKLLEAFVKRLKVRWQTAMNGSEAVKKWRSGGFHLVLMDIQLPGMNGLE
ATREIRRLERLNNIGVFSAAPGSIPETAPAEEELTEQDRLENIAKFKSPVIIVALTASSL
QSDRHEALASGCNDFLTKPVNFVWLERKVMEWG

>tr|B0DK70|LbicoSsk1|943-1101
SVLIVDDNPINQTILSTFMKKKKITYQLASNGQEAVEKWRTGQFHLILMDIQMPVMDGIQ
ATKEIRRLEKSNAASGYPPNLTPSTETEGQRTPSESSVSATSSESRPPASPYRSSVIIVA
LTASSLQSDRVAALAAGCNDFLTKPVSLLWLNNKIIEWG

>tr|A0A0A1UPZ7|ManisMcs4|694-844
NVLIVEDNPINLKLLEAFVKRLKVRWQSAMNGQDAVKKWRMGGFHLVLMDIQLPVMNGLD
ATREIRRLERINTIGVFTSTPDNATGNSEEDIKDQDRLQNPSLFKSPVIIVALTASSLQS
DRHEALAAGCNDFLTKPVNFVWLERKVMEWG

>tr|S2JKS9|McircSsk1A|1016-1203
TVLIVEDNMINQAILSTWMKKHSIKFSVASDGKEAVEKWKGGGFHLILMDIQLPVMDGIA
ATKMIRSIEKERKIGVLPSSSTFLADHDMAATEAANSTIVEEEPTKEKEEEEKEEEEEEE
DKTKKESPTPSASSSSNVSSFQSPVIIVALTASSLESDRQAALAAGCNDFLTKPVSLEWL
EKKIMEWG

>tr|S2JP13|McircSsk1B|1091-1271
NVLIVEDNIINQAILSAWMKKHKIKFSVASNGKEAVDKWKSGGFHLILMDIQLPVMNGIE
ATKMIRSIEKEQQIGVLPMSSSFLRQQQQEQQLQQEPLEVVAAAAAIASAAVNEMKLQQD
SNNTIKNDDKPSIFRSPVIIVALTASSLESDRHAALAAGCNDFLTKPVSLEWLEKKIIEW
G

>tr|F9X7W8|MgramSsk1|328-494
NVLIVEDNIINLRILEGLMKRLKVRWQTAMNGQIAVDKWRSGGYHLVLMDIQMPIMNGLQ
ATKEIRRLERVNGIGVFSSSAPNSPAELDVATTNGRPGEKKEAVKHDPADDKLAMEEGLF
KSPIIIVALTASSLQSDRHAALAAGCNDYLTKPVDFVWLERKVKEWG

>jgi|Mellp2_3|1955931|MlariSsk1
NVLIVEDNRINQTILATFLRKKLVRYDVAMNGQEAVDKWRTGSFHLVLMDLQLPVKDGIE
ATKEIREAERIMQIQTPPILGNPSNGSHPSSHSNSSHPISVIIVALTASSLDVDREVALA
AGCNDFLTKPVSLAWLEKKLLEWGS

>tr|G5EH08|MoryzSsk1|626-776
NVLIVEDNPINLRLLEVFVKRLKVRWQTAVNGREAVDKWRQGGFHLVLMDIQLPVMSGLE
ATREIRRLERVNSIGVFSSSADSAPDEVLGEPSEEDKLGNTDMFKSPVIIVALTASSLQS
DRHEALAAGCNDFLTKPVNFVWFEKKIMEWG

>tr|V5IP58|NcrasRrg1|872-1028
NVLIVEDNIINLKLLEAFVKRLKVRWQTAMNGREAVNKWRKGGFHLVLMDIQLPIMSGLE
ATREIRRLERMNSIGVFSNSNSNGGTNGGESGGDPDVMPEEDKLENIELFKSPVIIVALT
ASSLQSDRHEALAAGCNDFLTKPITYIWLERKVMEWG

>tr|A0A0A0HVY6|PbrasSsk1|611-779
NVLIVEDNIINLKLLEAFMKRLKVRWATAMNGREAVNKWRAGGFHLVLMDIQLPIMGGLE
ATKEIRRYERLNNIGVFSRTVSGQSLKGSSVSGRNLSGKAGNNNHLLEPLRPEDKLENPE
AFKSPVIIVALTASSLQSDRNEALAAGCNDFLTKPVNIVWLEQKVTEWG

>tr|K5X973|PchrySsk1|979-1129
SVLIVDDNPINQTILTTFMKKKKIKHDVAKNGEEAVQKWRSGIFHLILMDIQMPVMDGIQ
ATKEIRRLEKQNGQPFPSTPQSEGQQTPSETSTTDSRNMSTPPYRSSVIIVALTASSLQA
DRVAALAAGCNDFLTKPVSLQWLNSKIIEWG

>tr|H6QRJ2|PgramSsk1|1391-1541
NVLIVEDNRINQTILTTFMRKKSVKYDVAMNGQEAVDKWRTGSFHLVLMDLQLPVKDGIE
ATKEIREAERSVNINAFANTPPAVGGNTPPALVAPNTMPRMTPHNVSVIIVALTASSLDV
DREVALAAGCNDFLTKPVSLAWLEKKLLEWG

>tr|B6QED8|PmarnSsk1|277-434
NVLIVEDNVINQKLLEAFMKRLSVRWQCAQNGEVAVKKWRQGGFHLVLMDIQLPVMNGLD
ATKEIRRLERLNGIGVFSKTASGRSSATPSSSVPADTPLKEEDTLQDLSLFKSPVIIVAL
TASSLQSDRHEALAAGCNDFLTKPIGFPWLEQKVTEWG

>tr|A0A067NMJ0|PostrSsk1|793-947
KVLIVEDNPINQTILSTFMKKKKITYDVAENGLEAVEKWRTGSFHLILMDIQMPVMDGLE
ATKEIRRLEKSNAMAGFPPTPLAEDGSLALRTPSESSSSLIEARSSPYRSSVIIVALTAS
SMQRDRVTALAAGCNDFLTKPVSLKWLENKIIEWG

>tr|B8PEC3|PplacSsk1|676-829
SVLIVDDNPINQTILSTFMTKRRIKYDVAKNGEEAVAKWQTGGFHLILMDIQMPVMDGIE

ATKEIRRMEKYNVLGGFPSTPQSEGQRTPSEASATESRSSTMSTPPYRSSVIIVALTASS
LPSDRVAALAAGCNDFLTKPVSLQWLNSKIIEWG

>tr|A0A075B1D9|RalloSsk1|110-255
ENIINTQSTSILKLSVEEPNWLDDKISEGGSPASSLPSLVKVKPTKSILSVASNAGSNKI
TDELKNMGETHPLRILVVDDNIEMGGLETTKQIHLKLSKNEQPTIIAVTGSESTEESLQC
MKLGMAYYVAKPIGIMQLVDLLLKCE

>jgi|Rambr1|115309|RbrevSsk1
RVLIVEDNPVNHSLLVKFMKRKKIKYASAYSGEQAIDIWRKGTFHIILMDIQLPGISGFD
ATREIRRIEQMRESGGWIKNRVIIVALTASALKSDKEKAFEAGCDDFLTKPFDLVWLESK
IVEWGC

>tr|I1BQL2|RdeleSsk1|887-1043
NVLIVEDNIINQAILSAWMKKHQIKFSVASNGLEAMDIQLPVMNGIDATKTIRSIEKEQK
IGVLPMSSSFLQQQQAIAAAAANTIMAEEETPLLPPMLTEEDKNVTPSIFRSPVIIVALT
ASSLESDRHAALAAGCNDFLTKPLSLEWLEKKIIEWG

>jgi|Gloin1|9453|RirreSsk1
NVLIVEDNPINQAILSTFMKKKKIKYECASNGQEAVDKWQKGGFHLVLMDIQLPVMDGIE
ATKKIRGLEKSQKIGVFPSTPPSASSTPVSTPMKQTPPSTPDLGLIPVIIVALTASSLPS
DRTNALAAGCNDFLTKPVSLVWLERKIQEWGC

>sp|Q07084|ScereSsk1|505-647
NVLIVEDNVINQAILGSFLRKHKISYKLAKNGQEAVNIWKEGGLHLIFMDLQLPVLSGIE
AAKQIRDFEKQNGIGIQKSLNNSHSNLEKGTSKRFSQAPVIIVALTASNSQMDKRKALLS
GCNDYLTKPVNLHWLSKKITEWG

>jgi|Synplu1|653315|SplumSsk1
KVLIVEDNPINQTILSTFLRKRKIEYAVASDGREAVEKWKNGGFHLVLMDIQLPVLDGIE
ATKEIRRFEKERKAAALSPTNALGGEVSGEQRRLTTQLHTPVIIVALTASALQADRNTAL
AAGCNDFLTKPVSLPWLEKKITEWGC

>sp|P87323|SpombMcs4|363-505
NVLIVEDNIINQKILETFMKKRNISSEVAKDGLEALEKWKKKSFHLILMDIQLPTMSGIE
VTQEIRRLERLNAIGVGAPKLTQPIPEKDQLNENKFQSPVIIVALTASSLMADRNEALAA
GCNDFLTKPVSLVWLEKKITEWG

>tr|U7PTB4|SscheSsk1|480-628
NVLIVEDNIINLKLLEAFVKRLKVRWQTAMNGRDAVAKWRTGGFHLVLMDIQLPLMSGLD
ATREIRRLERVNSIGVFASTPSDSIGVGRDPQSDKLANLALFKSPVIIVALTASSLQSDR
NDALAAGCNDFLTKPVNFVWLEKKIMEWG

>tr|K1WMU7|TasahSsk1|669-811
KPFDVVVPPINVLIVEEYLEHVLPQEEDQTSDGEGRPRGRGEVAHGKLPFDPVMDGIAAT
KEIRKMEKHNKIGVFPSTPMSESSRPVEAVPAASPFRSAVIIVALTASSLQTDRVNALAA
GCNDLLTKPVSLKWLEKKTVEWG

>tr|R4XD49|TdefoSsk1|88-232
VVLIVEDNMINQRILETFMKKRKIRCLTAKNGREAVDKWKKGGVHLVLMDIQMPVMSGIE
AAKEIRRLERTNSIGVFSRQESSAGSVDEADKLTNLHFKSPVIIVALTASSLQSDRHEAL
AAGCNDFLTKPVSLVWLEKKVLEWG

>tr|D5G4P6|TmelaSsk1|297-448
NVLIVEDNVINLQLLEAFMKRLKVRWQSAVNGKEAVDKWRVGGFHLVLMDIQLPVMNGLD
ATKEIRRLERVNSIGAFSQSPSSPSREDAPAVPDEKDKLPNSILFKSPVIIVALTASSLQ
SDRHEALAAGCNDFLTKPVNFIWLERKVTEWG

>jgi|Usnflo1|879133|UflorSsk1
NVLIVEDNIINLRLLEAFMKRLKVRWSTAMNGRDAVTKWRDGGYHLVLMDIQLPVMNGLD
ATKEIRRLERVNSIGVFSNSASSTPIFPNGAGFNGMIDGEDSLMNTALFKSPVIIVALTA
SSLQSDRHEALAAGCNDFLTKPVNFEWLTRKVMEWGC

>tr|A0A0D1CFM5|UmaydSsk1|1322-1543
KVLIVEDNPINQRILSMFMGKKKIKYDVANNGREAVEKWKTGGYHLILMDIQLPVMDGIE
ATKEIRKLERSANIGILPNTPPAGAESRMEVPSSSLSGGSRTLTAKGLMKHAVAGCGNVG
APEGDKRLPSKQPGPKVSSTIGGSNASCSELVSGLGIADRVSTNIRGASGSINAFRASVI
IVALTASVLSSDRVEALAAGCNDFLNKPVSLPWLNQKILEWG

>jgi|Umbra1|237654|UramaSsk1A
QVLIVEDNIINQVILSKWMKNHGIKYEVASNGQEAFDKWSQGAFHLILMDIQLPVMDGIT
CTKKIRAVEKENKLQQMQQSLHREVPRSLSFDTLGSMEEKATEFRSPVIIVALTASSLDS
DRQEALAAGCNDFLTKPVSMEWLDRKITEWGC

>jgi|Umbra1|276918|UramaSsk1B
SVLIVEDNVINQAILSTWMKKHKIKFGTASNGQEALDKWNGGGYHLILMDIQLPVMDGIE
ATQRIRAIEKEQKTGKIPRQTVTDEPSVFRSPVIIVALTASSLASDREAALSAGCNDFLT
KPVDLVWLEKKIIEWGC

>jgi|Xylhe1|238871|XheveSsk1
NVLIVEDNMLNLKLLEAFMKRLRVRWQTAMNGRDAVTKWQTGGFHLVLMDIQLPIMSGLE
ATKEIRRLERVNGVGVFSSSVTSSTTPQTSPKQEPEDKDKLPNTDLFKSPVIIVALTASS
LQSDRRAALAAGCNDFLTKPVNFVWLERKVMEWGC

Unclassified RR (n = 44)

>tr|A0A1Y2HER7|CanguRR1|299-420
QVLVVEDDMINQRIIRAMLTKLGYKQITFAADGADALFHYNSMKEEGKYFDIILLDQSLP
SLSGDDTCARIRRNDRTQVIISCSANAQLTADPELCRALGYDEAINKPIYLDLLAEVLNR
WT

>tr|A0A1Y2HHN9|CanguRR2|3-124
FPTRKEDDVSNQRMIRTMLTRLGYSQVTFAADGADALFQYNNLKLDGKFFDIILLDSSLP
TLPGDDTCVRIRQHDRTQVIISCTASTHLVNDPELRRALGYDEAMTKPFHLDGLKEVLAR
WC

>tr|A0A1Y2H5R7|CanguRR3|42-99
RVLLVEDDAICRKLSGKLLSIFGCTFDVAQDGLAAVKAVNSGQYDVVLMDIVMPRMDG

>tr|A0A1Y2HQ52|CanguRR4|635-759
RVLVVEDNDLVRRINCSGLKRLGFTTIEADSGEAAVAVFERILRGAEPNVALVLMDLVMP
KMDGIAAARAIRAMGPPTWPVPIVAFTANAIGDEQETVMKSGHFCAFYTKGTSKGVGVLE
AIVSK

>tr|A0A1Y2HDC6|CanguRR5|42-130
RVLLVEDDAICRKLSGKLLSIFGCTFDVAQDGLAAVKAVNSGQYDVVLMDIVMPRMDGVS
AASQIRQFNTWTPIVPVTSNISEDDRLTY

>tr|A0A1Y2HHP3|CanguRR6|1-109
MIRAMLTHLGYSQVTFAADGADALFQYNTFKLDGKFFDIILLDSSLPTLPGDDTCVRIRQ
HDRTQVIISCTASTHLVNDPELRRALGYDEAMTKPFYLDGLKDVWHGAT

>tr|A0A1Y2HRV0|CanguRR7|296-417
QVLVVEDDMINQRIIRAMLTKLGYKQITFAADGADALFHYNSMKEEGKYFDIILLDQSLP
SLSGDDTCARIRRNDRTQVIISCSANAQLTADPELCRALGYDEAINKPIYLDLLAEVLNR
WT

>tr|A0A1Y2HAQ9|CanguRR8|1-92
MDGLECVKRMNDESKPKVDLILMDLVMPNMSGMDAMKTLRDAGYSKDALPIVAVTANALP
EEREACMEAGFNEFVTKPLKKDGLVDICKRFI

>tr|A0A1Y2H4R8|CanguRR9|13-148
RVLVVEDNPIATRILSIFLARRSIEHVTVTTGPQAIDHFAHSGPFHVVLMNLGLAQQPGQ
ADMDGLECTRAMRQFEHEHGGGGDGAHGIMPPAVIIALTADASAETHRAALLAGCNDVAV
KPVRLTWIADKLVEVA

>tr|A0A1Y2HN41|CanguRR10|135-296
RVLVVDGDFRHARLVVAQLANAGYPVDVVDSTSAALTRLRRPPPMGLLGGWHPQNGACVD
EEEESGQVSPSAGSSSVARHVPPPPPVCPEYSLVICDISTVQGIELLRSIRSDLDLLLVP
VIMMASVDEMELAYSCLRAGADDYLVKPVKHEAVKGVWRSVW

>tr|A0A1Y2HAQ1|CanguRR11|247-367
RILVVEDNALVLRVTCKLVKTLGYEADTAMDGLECVKRMNDESKPKVDLILMDLVMPNMS
GMDAMKTLRDAGFSKDALPIVAVTANALPEEREACMEAGFNEFVTKPLKKDILIGLCSGF
L

>tr|A0A137PDE4|CcoroRR1|769-877
NVLIVEDNPINQTILATFMRKRNISYSVANDGREALQKWKDGSFHLVLMDIQLPVMSGIE
ATKEIRRIEKTRKIGCFPSESPTQQSLTADRTEALAAGCNDFLTKPVSL

>tr|A0A137PHN8|CcoroRR2|12-137
RVMIVDDNLIYHQLVTRLLKKKLGICHIINCINVEQAIAQLNERPFDLILMDIDMPLITG
VMATQQIRDPLNSWNILESNRTIPIIALTTNSLEEDYKLYMKVGMNGMIPKPVVEKTLRT
TLQECL

>tr|Q6SLC4|CheteRec1|205-325
RILIAEDNMINQKVMLKFLRRLGLNDIVTASDGQAAVNIVQECMDRYQPFDLIFMDDTMP
VLSGHDATHLIREMGFRGPICGMMVNLRLANKQKAFDAGITTIIHKPFQLRAIREVLEQY
L

>tr|A0A139A6V1|GprolRR1|113-234
RVLLAEDNPLSRMVATKFLGKLGVRADDAHDGQERLICVNEEMGTGRGMRSCSWMDKCQL
TGLEAAHAIRSLLPPFRQPYIIALAASVTEGDITQAQSAGIREHLGKPVSLEDLTKALER
AV

>tr|A0A139AN39|GprolRR2|100-232
NVLIVEDNHVNQMLLSSYLKRRGINCALAVNGEEAVQKWVEGKFHLVLMDILMPVMDGIE
ATRQIRQIERTRREQRSRLGTFTGYHAADVVIVALTASTSPTDRDEALAAGCNDYLTKPV
SLIWLEKKIIEWG

>jgi|Ganpr1|468420|GprolRR3
HVLVVEDNIVVAKVTSKMLARNGYQVDLADDGLKCVQRVKVRALNGYDVILMDLHMPNLD
GVGAAQQLRELGFDRAIVALTAYTAQEDQRKCLDSGLMQAVLGKPVKEQDLINCVTRYSG

>tr|A0A0A1UZ69|ManisRR1|213-331
AILIAEDNKVCCLVLAKLLFKLGLNFKVAQNGQEAIDEYKRDPKRYWCVFMDLGMPVVDG
DTASRWIRGFERATGLEPCVMVSMHSGRRETGLKTDVFDYFLDKPIRRSGIESILRAEK

>jgi|Morel2|156655|MelonRR1
NVLIVEDNMINQTILIKFMQRRKIKFDVACNGREAVDKWRVGGFHLVLMDIQMPVMDGIE
ATREIRQLEKAQRIGVFPTDKPNSIGNTMSLMAASASASSSATSPQGTTTPSSPFRSPVI
IVALTASAESEESRNTALLAGCNDYITKPIDLPWLERKIVEWGC

>jgi|Morel2|70033|MelonRR2
KVMIIDDNSVNLTILSRLLNKHFADTIQLTSVQNSGLAALDQLTKDEVDLILMDIDMPVL
SGVETTTAIRNNDSHSENSILEQNMKVPIIAVTTSDAEEQRELYRQVGMADCVSKPIAIP
KLRMAIEEAMK

>jgi|Morel2|73814|MelonRR3
KVMIVDDNSINLSILSRMLEKHFTDVIQRTVIMTSGVEALKRLATEEFDLILMDIDMPVL
TGVQTTVAIRQNSSECPVLEHNQNVPIIAVTTSDGAEQRELYQQVGMSDCVSKPIVLSTL
RSAIEGAMK

>jgi|Morel2|57438|MelonRR4
KVLVVDDNPVNLKVVCKMLGRLGVEPETANNGQEAPAGTGPHIVPYDLIFMDIWMPKMNG
LDASSYIRKNLSGNTPDRPYIIAMTACVMPGDREKCIASGMNDYISKPLRKEELEQCLRL
FT

>jgi|Morel2|60291|MelonRR5
VNQKVISRMLGRMGIKPDLANNGQEAIDKCKARAEAYDLIFMDVWMPVKDGLEATEEIRK
SVDGITGTEPFIVAMTACVMPGDREKCLASGMNAYLSKPIKKEELCSILEKWLD

>jgi|Mycgr3|29210|MgramRR1
RVLVAEDNKVNQEVVLRMLKLEDIYDVTVAKDGQEALDLVKESMCGDERKAFNLIFMDVQ

MPNVDGLQSTRLIREIGYQAPIVALTAFAEESNIQDCLDSGMNYFLSKPIRRPQLKKVLR
EYCA

>tr|F4R5X0|MlariRR1|78-206
HVLVVEDNLINVKVIKRQLSLKGYSVSVAMDGRQGLDILYEDDQHPSELGRIGIVLMDIQ
MPVMNGLDAITELRASEKTGKVKQRYPVIAVTGNARKEQTEQFLASGFDDICVKPYKIED
VQNRMEALL

>tr|F4S2B6|MlariRR2|31-162
RILVVDDNDLNLRVMRQILSKKTPDLVHLPSLRVASSGLQALALLSQHVFDLILLDISMP
GISGIELCRRIRDLDPNQKSLVLNHNRTIDICAVTTDLQDWQIEIYKKVGMNGVIGKPLK
EQDIRYALQLSC

>tr|F4R5X1|MlariRR3|7-122
TILIADDNDDMRSFVRSVLSRYYTVAEASDGLEAYHWAKAHHPDLVVSDAMMPALNGFEL
LKRLKTDPDTAGISVILLSAHAGSEIRVEGLAEGADDYLVKPFEAKELVARINTHL

>tr|E3L225|PgramRR1|22-146
KVLIVDDNLLNLRVMKQLLKTKLADFLDLDSLQTADSGFEALDLLRNNDFHLLFLDISMP
GISGLEVCRRLRNDSRYSSSKLHICAVTTDLADWQVQLYKSIGMDGVIGKPLKNLDLEFA
LRACL

>tr|A0A067PB73|PostrRR1|345-459
RVLLVDDDAVIQKLSSRLLRIFGCTIDVATDGVAAVHKMNTAIYDLVFMDIMMPRLDGIS
ATCRIREFDSMTPIISMTSNSQQSDVQVYYTSGMNDVLCKPFGKEGILMILEKYL

>tr|B8PJM0|PplacRR1|804-918
RVLLVDDDAVSRRLSSKFLQVFGCTIDVAVDGVGAVNKMNLEKYDLVLMDIVMPKLDGVS
ATSLIRQFDHMTPIISMTSNSKPNEIIKYYSSGMNDILPKPFTKDGLLDMLEKHL

>tr|A0A075AN70|RalloRR1|140-257
CCLIAEDNPIIQKVAQALLKSIGFQTTCVDNGLKAVEECQKVKFDIIFMDIMMPEMDGIE
GTKIIRSSKGINEKSPIVAFSAIGSDENRKEFISQGFNDWLEKPFTKNDLYKMIKKWI

>tr|I1CAK9|RdeleRR1|103-217
RILLVDDDSVYRDLSERLLQVFGCTIDLAKDGVEALKKMSLERYDLILMDIVMPKMDGIS
ATRSIRQYDALTPIVSMTSNFTDNDIVQYIGSGMTDILPKPFSKRTLYQMLDKYC

>jgi|Gloin1|20972|RirreRR1
KVLVVEDNAVNRMILVTFLKKRGIRLDEAENGAIRPFTQKNRILQRRVSINSQNNSFSIT
SLLLKPKISIKYLNQQQNSKHHLFTIPSPIPSSPSFPIIENNTINTITKRSHSLTGLASE
EDKDLAFESEVDGFLTKPVSLKMLEKVLKKWSE

>jgi|Gloin1|349496|RirreRR2
TLIVDDNVITAKLLSKILSKEFGHDTTCTISGKDALLKLSRETFDIIFMDIDMPELSGIE
TTLLIHAKNSNVLEQNRHIPIIAYTTNPCEDRFFEAGMTGWVGKPAKQMMVRQELERVYR

>jgi|Gloin1|80670|RirreRR3
KTLVVDDNIITGKILAKILEKEFNHKVKCVVSGNEALELLSNEMFDIVFMDIDMPKQNGV
ETCIKIRNTSIVLEENRKIPVLAYTTNPWEESFDQAGMNGYIAKPASSAKVQAELEKVYQ

>jgi|Synplu1|312668|SplumRR1
PLALCCDDNPINLRLLLRRMESVGFDCLAATSGEEAVARAKQLRRSDPPQMLDLIMMDFH
LPGIDGIEAVHQLRELDWPREPLCVLNTADTSEDLKARASSAGIWEYLTKPFPVDVLRSY
RDRINL

>tr|R4XFZ6|TdefoRR1|49-172
EVLLVEDNKINQTIAVNMLKKMNFKTRVANHGQEALDILHAGSDGSYMPSLVLMDCQMPV
LDGYETTRLLRNDFISAIRRLPVIAMTASAIRGDKEKCLEAGMNDYLSKPVKQEQLGQIV
AKWM

>tr|R4XIL3|TdefoRR2|1-113
NQVIAQKTLQKLGLAVHVAENGQEAIDYLISTPAKDLPEIVLMDCQMPLLDGYEATAQIR
KLVNSRIRNLPIVAMTASAISGDREKCLAAGMNDYVSKPINSVLLSQTLRKYL

>tr|R4XF21|TdefoRR3|118-237
RILLAEDNLINSRVALQHLKRMGYSAVHAKDGQYCLEEEDKAIYDVILMDVQMPRLDGCQ
AASKIRAKYLKDPERCPRIIAMTANAMRGDMERCLEAGMHAYCQKPIIVDVLAQRLREAT

>tr|A0A0D1DUG2|UmaydRR1|28-154
RILLVDDNHVNLSILSMLLKRRFSHVLARPPVSLDSGLKALQLLRTEIFDLIFMDIEMPY
LDGVECARRIRAGEDGILSANRNAHIVAVTTNVGPEPASLYRHVCMDGMISKPVRFENFH
QYLCPLS

>UPI00003C2556|UmaydRR2
FVVLVVDDNPINRKVLTIPLRKQGIQVVEAENGLEAVQRYAQIRPALVLMDISMPIMDGF
EATRQIRMHEQAQSTSSSDFVAAGALDEMINMVLTHQRARIVAATTHSADRDFDEGKQAG
MDDWLLKPIRPSVLVQDILEYRR

>jgi|Umbra1|260787|UramaRR1
SQVVIENHGHAALAGIRERLQKNYMPTILLIDLDTCPHANQLVHPSISLLQDVVKELKAG
TLHDVAPVVCSTNDSAKLMLQCINEGAADYILKPLRKDVLKTMFLNLHR

>jgi|Umbra1|248240|UramaRR2
MKVLLVDDNLINLAILSQTLRKHMAHLVEHLEVAKSGVKALELLKLHSYDLILLDIDMPI
LNGIETARHIRQSTAEYDVLACNRSIPIVAVTTNDSAEWKRAYYDVGMNGCVSKPIAVDI
LKKTINGVLT

>jgi|Umbra1|221206|UramaRR3
RVLLVDDNDVNLRILERLLKKHLSHIIHTVHVAQGADQALQLLSTIKFDLVMMDIEMPGM
SGVEAAARIRAGMAGERHRLIPIVAVTTKYSPYWRSLYLENGMNGCISKPIVLDTLLDTV
NDILC

Group I HHKs (n = 50)

>tr|Q4W9C4|AFUMIFHK1|1005-1141
HVLIVDDNDINLKILATFMRRIGCSYETATNGLVALEKYQQAQRQFNYVLMGRLPLLGVM
VQLTAIDLSMPVMDGIISTSKIREYEEENSLPRAAIMAVTGVASATMQQQAFAAGIDDFL
VKPLSLRDLKRVMMNIA

>tr|Q4W955|AFUMIFHK2|1118-1249
SVLLVDDNDVNLKLLVAFMKKAKFPYYTASNGLEALEVYKANAGYIPVVLMDISMPVMDG
LEATREIRLFEKMHREGSTNAGHSCFKPTTVIVLSGLGSAPVRQEAFNSGIDLFLSKPIR
FQELVKQIDELM

>tr|A0A0U8TB10|AFUMIFHK3|1140-1263
RVLVVDDNSINLNLMLTFMKKQELEVLDSAENGKVAVDAVERMQQGYDLIFMDISMPVMN
GFEATRAIRALEKERDGCGPATIIALTGLSSSRDESEALTSGVDLFLTKPVSFKEVSRLL
DEWA

>tr|Q4W989|AFUMIFHK5|1076-1211
NILLVEDNQVNLKLIEMCVKTTGFSYGTAKNGLEALEKFKTGTYDAVVMGELTSVILHSA
HLTSADISMPVMDGLTATRQMRAFERKNKRPATTIIILTAVLSASMQHEAMMSGVNLFLT
KPTPLKQLKEILRNLS

>tr|Q4W955|AFUMIFHK6|1118-1249
SVLLVDDNDVNLKLLVAFMKKAKFPYYTASNGLEALEVYKANAGYIPVVLMDISMPVMDG
LEATREIRLFEKMHREGSTNAGHSCFKPTTVIVLSGLGSAPVRQEAFNSGIDLFLSKPIR
FQELVKQIDELM

>tr|G1XJT9|AOLIGHK2|1276-1395
SILLVDDNPLTMNLMTAFIAKKGFLFSEASNGLQALETFRGRGGGYDIVLMDLQLPVMSG
LEAVKAIRDLERTENRLKRAFIIALTGSSNEAEALAAGCNKFFTKPIRLLDLSNLITEYQ

>tr|A0A384JVS8|BCINEHK6|1082-1234
HILAVDDNALNLQLIHRYLQKRKTDTIVSAADGLEAVAAVKASKNGFDVIFMDISMPKMD
GFEATRHIRDWESELCVGLDGLIGWGHGREKEKEMEAEGKTDGELSRGKAYVVAMTGLGS
QKARDEADRSGFDDFMTKPVKLPKVGELLKRLS

>tr|M7TQA4|BCINEHK9|1223-1359
NLLLVDDNMINLKLLRHFITRLGYTTVHEAENGLEAVKKVEERPEGYDIIFMDISMPILD
GFDATKEIRKLEKSRRERAIINDGIDKGEKKNEALVVALTGLAGKDDVEKARACGIDLFM
TKPVAMKEVRKMLENWR

>tr|G2Y0B1|BCINEHK10|1165-1286
IALLVEDNTINMKILIACMKKLSVQYYCAVNGLHAVTTYQNARVKPDIIFMDISMPIMNG
FEASRKIRGLEKKEGWKASVIVAVTGLASDESQREAYSSGINLFMSKPVPLKELGKIVVE
ME

>UPI0001585802|BcineHK16
TVVLLVEDNAINLKLLVHSMKKLKEAYDTACNGLEAFEKYKKAPGSYKIIFMDISMPIMD
GLTSARCIRDHESLYNLPRVRIVALTSFGTEEHRRDAAMSGIDLFLTKPISMKSLKPVVD
LNPE

>tr|A0A384JY60|BCINEHK17|1067-1194
TILCVDDNAINLRLLRAYFRKLNFDITCAENGAVAFEKYRLQPNGYDLVFMDISMPICDG

YQSTRMIRSLDKLQQSISPTPLPATRIVAISAAYSDADMEMAKAAGIDDFYTKPMKVSRL
ETLMKNWG

>tr|N1J8K3|BgramHHK2|1213-1354
RILVVDDNEINLQLLSTYLRRRAYPQIDNAHNGLEAVQKCEAMANSYDIIFMDLTMPILD
GFGATRKIRAMEELRRMHVDLGEGSTSEPSSSKSGFVSAFIIAFTGRSSIEDQNEALSCG
IDLFMTKPVAFKEVGKICDNWI

>tr|N1JG60|BgramHHK9|1096-1217
RVLLVEDNKINLRLLVATMKKLKTAYATATNGLEALNTYKEHTGKFDAIFMDISMPVMSG
IESSKNIRTYERENNLSPVVLIALTGAANESTRQEAFSNGIDIFLTKPVPMKALGNMLEV
LS

>tr|D6RM33|CcineHHK4|1389-1514
RVLVIEDNNILRNLLAKWLIKKGYDFEEAVDGQAGVTAFQEHGPFDVALIDLSMPVLDGV
AATKEIRRIESSKAGGDDSYPSVKILALTGMSSLEDKRRAFEAGVDGYLVKPVAFKTLDE
MFHKLG

>tr|Q6SLE3|CheteHHK4|1085-1210
RCLCVDDNPINLRLLRTFVNKLGHEHTLAVDGLEALEAYIAANNEEESRIDVVLMDINMP
VMDGLEATRQIRAHEIRHNLPKVTIIALTGVADTDIQQEANSSGINLFLIKPVRLADLEV
ILKGVV

>tr|Q6SLE0|CheteHHK7|1031-1152
HILIVDDNMLNRRLLVAFMNKNDLQHQEASDGLEALRKYQADPHKFEVIIMDMSMPVMDG
MAATRAIRKHEQNHNLPRSCIIALTGLTSSSARLEAWSSGIDHYITKPVNYQKLRELLKS
EK

>tr|Q6SLD8|CheteHHK9|1020-1147
RVLLVDDNAINLKLLVVFAKRQNLRYVEATNGLEALETFKAESRSTASPSRPFEFVLMDL
SMPVMDGLTSTRQIRQYEAKMGLPKSHIVALTGLASAQDQMDAQEAGVDMYLVKPVKFAD
IKRIFGGK

>tr|J9VEN4|CNEOFTCO6|1474-1620
RVMVVEDNVINRRVLGAFLKKRGFEYAEAVDGQAGVELFENAPPNYWDVILMDISMPIMN
GHQATRAIRRIEATRRNSLSDIPIVPPPSKPVTISSQKAVQARVKIFALTGLATPDDKRE
AFWSGVDGYLVKPVSLSSLDIIFKKIG

>tr|M1VUX2|CpurpHK9|1163-1284
RFLVVDDNPINLKILSSCLQKMGHLHDAVCNGKEAVHAFRRGAGAYKGIFMDLSMPVMSG
FEASCLIRQHEKEAKLETCTIFALTGLASAEAQQEAFACGIDLFLTKPVRLKELRQVLTS
KG

>tr|Q6SLA0|FVERTHK2|1095-1226
HVLLVDDNKINLNLLTMFMKKCGFSYEEAENGEEAVDTFKRSTIGDAEQGAPVKKHFDYI
LMDISMPVMNGVEATKRIRKIEAEYKVPRTTVFALTGLASADARQDAMSAGVDLFLPKPV
KFAELKTMIENN

>tr|Q6SLA2|FVERTHK4|1060-1181
EFLLVDDNFINLKILSSYMKKLKQPYQTASDGLEAVTAYEADPGRYSCILMDISMPVMDG
FEATRRIRAFESQQGLRPALILALTGLASEEAQREATVSGLDLFLTKPVRLKELGPILRA
KG

>tr|Q6SLA3|FVERTHK5|1127-1248
SFLLVDDNPINLSVLCAYMKKLKSKYITAADGLEAVERFQESPDEFSCILMDISMPRMDG
IEATKEIRAFENKEGREPVIILALTGLASASAQQDAYACGVDVFLSKPVKLKELSSILRE
RS

>tr|Q6SLB0|FVERTHK6|984-1103
QFLLVEDNPINLKMLTHFMRKLQKPYRTAVNGQEAVNAFKETPGQFKCVLMDISMPVMDG
LEATRQIRAFERYSGIAASKILAITGLGSESTREEATRSGVDVFITKPIKLRELAVIINS

>tr|Q6SLA9|FVERTHK11|1118-1239
RFLLVEDNAINMKILQTYMKKLGVAYDSASDGLRALECYKAQEGCYRCILMDISMPVMDG
FEATRQIRGFEKASDLPRRHIVAISGLASKDAQEDAFANGLDLFLSKPVQLKELSRILKS
RG

>tr|G0R854|HjecoHHK1|967-1088
KFLLVDDNYINLKVLSTYMRKRNVAFKEAKNGQEAVDCFLSHPGAYACILMDISMPVMDG
FEATRQIRVHEAQMGLTPVPIIALSGLATEDAQQEAFGSGMDVFLTKPVKLGALGSLLES
HG

>tr|G0RFR9|HjecoHHK2|1012-1133
EFLLVEDNSINLDILSIYMKKLGRPYHTATNGAEALSAYKSNPKHCKYIFMDVSMPIMDG
FEATRRIRAYERDNQIKPAIIFALTGLASESAQQEAFGSGVDLFLTKPVKLKELGMILRS
RG

>jgi|LacbiH82_1|506531|LbicoHHK3
RVLVVEDNSILRNLLIKWLSTKGYDFRDAVDGRDGVSVYETEGPFDVVLLDLSMPVLDGV
SATAEIRRIEKSHPDDSRITRILALTGMSSLEDKRRAFEAGVDGYLIKPVAFKTLEEMFR
RLGI

>tr|A0A014PP15|ManisM232|1069-1190
SFLLVDDNPINLKILITYMKKLRLPYRTATNGQQAVDLFREGNGLYKCVFMDISMPVMDG
FEATRHIRSTEMEKGLHRCTIFALTGLASADAQQEAFTSGIDLFLTKPVKLAELSQILSA
RF

>jgi|Morel2|1828054|MelonHK4
RILIVEDNITNRMILRTFFKKNGVVTVVEAENGQIGLHRFEEELVRQGPGGRAAFDFVLM
DLQMPVMDGNMATKRIREAEGKWIKALEGKYCPSTIFALTGLAGEEDKRLAFECGVDGYL
TKPVSLKGLANLLTTCR

>jgi|Morel2|1828755|MelonHK5
RVLIVEDNITNRMILRMFLKKRGIGVVEAENGLLGVERFQEEVWRRDGKAGFEFVLMDLQ
MPVMDGNMATKKIREFEQGLVRQGQLNVNRANGFQTGPPSTDQEGAYRRTTIFALTGLAA
DEDKRLAFECGVDGYLTKPVSLKILGELLETNP

>tr|F9XGU6|MgramHHK3|966-1098
SLLLVDDNPINLRLLTTYAEKYSHPYRGASNGLEAAQAYESAASKFPHATMSNAPKPDVI
FLDIQMPIMDGYEAAQRIRAFEKTAGVKPAIIVAITGLASNQSQQDAYSSGIDMFLTKPV
RPRDITRVLESIS

>tr|F9X4G2|MgramHHK4|1055-1213
RTLLVDDNELNLSLLKTYMRRNGHGYTCARNGVEAVHAYKEAYSASFDSTTPILVLMDLT
MPIMGGLEATRQIRAFEREQVHNSDRGSRSTSNAGLSGEDMDSRRSSLKGSHPPSAVVVA
LTAISGSETKMAAFGSGVDLYLTKPVGFKNLGEILKGLL

>tr|F9XCM9|MgramHHK9|911-1042
SVLLVDDNPINLTLLRRCIKRLGRIDLAAVNGQEALLVYKESHSHRLEHVRTSSDGKSTR
TAPVRFILTDITMPVMDGLEFTRRVRAHERALDLKPAMIVALTALASSADRHEAYGSGVD
LFLTKPFRYKDI

>tr|G4NKR9|MoryzHik3|1143-1268
RFLLVDDNPINLKILVSYAKKLGRRFACATNGLEAFEAFRESIGQGADAFKFVLTDISMP
VMDGFESTRRMRSLEMERGLPRCNIFALTGLASAGAQEEAFASGIDLLLTKPVRLKELNK
ILESKG

>tr|Q6IFT0|NcrasHHK7|1407-1529
ACLLVDDNPINLHILASAMRKTHRPYATARNGLEAVEIYKENPGRYKWVLMDISMPVMDG
LEATRKIRDTEGELINGLEPATVVALTGVTSGTIQKDALASGVDLFLTKPVRMKDLGAIL
GDG

>jgi|Phchr1|4508|PchryHHK4
RVLVVEDNAILRGLLVKWLRSKGYDYCEATDGLEGVGATVKMRDVEALRKKQGQIDRHVQ
DTRILALTGMSSLEDKRRAFDAGVDGYLVKPVAFKTLDMMFHKLG
>tr|B6QJ11|PmarnHHK1|1076-1197

HVLIVDDNDINLKVLSTFIRKLGHTYETASNGLIALNKYKESSTPFNLVLMDISMPVMDG
LVATSQIRGFEKQKSLSPVTVLAVTGVASATMQEQAVAAGIDNYLIKPLNLQQLKRAIEK
SM

>jgi|PleosPC15_1|33985|PostrHHK4
RVLVVEDNSILRTLLVRWLKTKGYDYRDAVDGRDGVNIYQAEGPFDVVLLDLSMPVLDGI
GATSEIRQIEAAREQSDNKPARACILALTGMSSLEDKRRAFEAGVDGYLVKPVAFKTLED
MFHKLGIT

>tr|B8P4B7|PplacHHK4|1456-1588
RVLVIEDNSILRNLLVKWLRNRGYDFREAVDGEEGVRIFESDGHFDVVLVDLSMPVLDGV
GATTQMRSIELARSMNGSPSSSAESSAHAARILALTGMSSLEDKRRAFEAGVDGYLVKPV
AFKTLDTIFHKLE

>jgi|Gloin1|138626|RirreHK4
RVLVVEDNAVNRMILATFLKKRGIRFDEAENGAIGVENLKKH

>jgi|Sporo1|31634|SroseHHK5
RVLIADDNAIGRSILTKLFAGKGIDFAQAENGQEAVDVYAAEKGRFNLILIDVQMPVLDGI
EASYEIRKYERANGLPRCRIVALTGFAEDHQHHSSSSSDRSSTDDIDSNPRNAVDSWLVKG
GKSLAIILREVKN

>tr|K1VIT0|TasahHHK5|784-952
RVMVVEDNAINRRVLTAFLRKKGFGYSEAVDGAKGVQLFRDTPPSHWDVILMDINMPIMD
GLEATRMIRTIEASRRHCVKSPNGDSKPKLMGNEISGKGADTSNAEDTPPTRDPPKSSRA
HDPTHVKIFALTGLATGEDKRTAFNAGVDGYLVKPVSLSSLDVLFKKIG

>tr|K1VMH1|TasahHHK6|641-758
TVLVVDDAPVNIQVCRRILELYGYKDVDSATDGLQATELAEKRRYDLILLDLQMPVLDGF
GALERIKASPLAGEPCCVSLSANVDKATRDRCSDVGFFAVLDKPVDIPRLGEILIKVH
>tr|R4XCB7|TDEFOHK1|982-1103

HCLIVEDNPINMMLLVAFAKQRNITVTTAVNGALAFERVKERPRSYDAILMDINMPVMNG
FQSIEAIRKYERENEQTPAKIVALTGLSTPEDQKLARDLGADAFFTKPVKMRDLAELFVE
WK

>tr|R4XE51|TDEFOHK2|846-971
RVLIVEDNPTNSRLLERFMQKRGVVYTAVVNGQEAVEEIDSIGTGDKQAYDMILMDIQMP
VLDGLEATERIRRIETSKFMPKAQIIACTGLSAPEDKRRASEVGCDKYLTKPISLVTLKH
LFDDWS

>tr|D5GIJ2|TMELAHK4|1280-1401
TALIVEDNPVNLMLLATFMKKRGYPFEKATNGLEALRAAESRPGGFDIILMDLQMPIMSG
IESTRAIRRLERQSRAKGSVIIALTGLAAASDKVEAYEAGIDLFMVKPVSFKQLESTMRE
YS

>jgi|Usnflo1|690143|UflorHK1
RLLLVDDNKINLRLLETYMRKRKYQLVDSAENGQLAVEAAESHEHGYDIIFMDISMPIMNG
FEATRAIRDIEEARTHNDNAGSHTPSSSPALIIALTGLASSRDQSEAFTSGVDLFMTKPVS
FKEVGRLLDNW

>tr|A0A0D1DQL8|UMAYDHK3|1992-2147
QILSVEDNAINRKVIAAFLAKLEVDFVEATNGEEGIAQFSRYPPNHFDVILMDLSMPVMD
GISAIAAIRKIELERYQGGKSDSGSSGNNSSSTHTIGSLGRTGTNKLVARQRSKIFALTG
RSTDEDKRQAFQTGADGYIVKPLSFKVLSSLLRMLM

>jgi|Xylhe1|266356|XheveHK2
KVLLVDDNQINLRLLRTFMKKRKYDLVDMAVDGFLAVEAAKKTCYDIIFMDISMPRLNGFE
ATSQIRELEEERRATHLRSDQAGPSPSPAFIIALTGLASSKDQKEAFEVGIDLFMTKPVVF
KEVGKLLDNW

>jgi|Xylhe1|280918|XheveHK3
TVLVVDDNEINARILTTYMKKLGCNYMVAYNGLEAFEAFKAEPARFSVILMDISMPIMDGL
DSTRHIRELEKQLRTPKPATIVALTGLGQEGIQRDAIASGMDLFFAKPVPLKQLAQVIEET
K

Group II HHKs (n = 11)

>G1XKT2|AoligHK4|854-978
RILIVDDNPTNLGILVMYCKRRKHDYVTAVNGKEAHQKYLEAAAEDKCISLVLMDLDMPV
KNGIDATHDIRVSEATEGLQPSMVFMVTGQNTDEDKEKSVAAGANGYYVKPLSMKALDDL
ISLHF

>tr|J4KMC9|BbassHHK3|861-990
MVLVVDDNLVNLRILQMYCKKRGLPFLSAANGTQAVEVFSKHQASSAAGGGAGIELVLMD
LQMPVCDGLEATQQIRALEKKNFWAASVVFMVTGQDSQSDREAASAVGADDYMVKPISMV
RLDTELKRYF

>tr|Q6SLE4|CheteHHK3|869-1004
RALLVDDNTVNLRLLQMYCDRRGISYQTAKDGLQAVKIFSEAVITRYDPLLQRDVSATAF
DLILMDLQMPECDGIDATRQIRRLEEENKWEKSVLFIVTGQDSPTDRKNAEEAGADEFLT
KPLGPKVLDQWVKKWY

>tr|M1WFS9|CpurpHK7|876-1008
TALLVDDNIVNQRIMQMYCKKRKMSYHCAADGAQAVDIFSRQQSLMAAAGPGEEQGIQLI
FMDLQMPVCDGFDATRQIRELEREHGWPESTIFIVTGQDSPSDKKTAEMIGANEYFVKPV
GPKVLDVGVKRYF

>Q6SLB1|FvertHK7|862-982
MTLIVDDNAVNLRILEMYCKKRGLPYLSAIDGHQAVELFKKQQTSAPIDLILMDLQMPVC
DGISATRQIRAMETSSKAVLFIVTGQDSLMDREAASKAGADNYLVKPVGIRMLDSSLKRY
F

>tr|A0A4P9YS74|RalloHK6|432-556
QVLVVEDNPLIMRMMLMFLKKSNFTFDIAGTVKLFSLLESGELALELWDKTNFEVVLMDI
SLPKMSGVDVARHILKCTDRNIPHMIAITADVNFENSGEQTKLFEKVLTKPVELKFLKGM
ITEYL

>jgi|Gloin1|94431|RirreHK8
KVLMVEDNETNQRIALHYLEKMGQKVTLAANGIEALEKMKNETFDILFVDLQMPYMDGYT
LTRRIREAEREAEREAEQEACRCDKQDCESLLFSVSESSTPPPTPPGSSSALTNGHQSVI
PLVSLIQSSQSISPLHINTQPPIISSTSSSLSSLSSSNYSYSSSSSSSESSSSSYSQSLL
PSPTTTSGSTKNNVTIKNSHQSVPSSPPISPPSSSHKRLKHVPIVACTANVLDEEKGRCK
QHGMDGFLSKPYKMEQMKQCIEELVE

>jgi|Gloin1|6899|RirreHK9
NVLVVEDNAVNRTILTTFLKKRGIRFDEAENGAIGVEKFKKALEDDDGENPNRKGFDIVL
MAITTNSTLAKSPAASSTITNFKFPPSSLFNKTIPSSPVSESNNHLTHLLKRIDFYKEEY
QIMIHSKRSTADKFEIEPNNFRFMKAIENNFALYDSDKARSLAKKPVYQKFSRKWVDSFM
S

>jgi|Synplu1|252105|SplumHK4
RGLVLYVEDNAINQKLGALFLKKLGYQAELAGDGQEAVRKVTETEDIKYDIILMDCQMPI
MDGFEATRLIRKHEANHWISSLQQSKSPSTSRPSTDTASSNGPTAAVAGASTASGVTVSR
TPIIALTASTFDSWKTRCYEAGKYY

>jgi|Synplu1|682744|SplumHK5
TQFVEDNFVVRKLIVRMLGRLGMVNVVECENGLVAVQQCRKEHYDLVLMDCGMPVMDGFE

ASRQILPRDSDSKEAHTEGSSSCQICKESQVRVTKLTTSTTNRIDYCILKPVRFDDLHEV
VRQLAV

>R4XNI5|TdefoHK8|450-572
SCLIVDDNKVNLTILSTFLKRKGYNCHVALDGGEAVAAVKAREACRQFDIILMDINMPNM
NGVEASRRIREYQKSKSLTQSMIVAVSGCSDAEQQSAMARGMSHFFKKPVNLKALARFLE
KPD

Group III HHKs (n = 57)

>tr|A0A068RNC3|AcoryHHK1|1565-1684
SVLLVEDNIVNQRLAVKLLEKMGHEVTVAQNGQEAVAMYEEESIDVILMDIQMPVMGGFE
ATALIRKYEKENERPRVPIIALTAHAMIGDREKCLSNDMDEYVSKPLRMPELAAAINRFM

>tr|A0A068RXI9|AcoryHHK2|2277-2397
EILLAEDNPVNQKLAVRILEKFGHHVHIVANGQLAVEAFQNKRLDLILMDVQMPVMGGFE
ATQQIRRLEKSSGTNAHIPIIALTAHAMIGDREKCLNAGMDEYVTKPLRFPELIAAIKKF
A

>tr|A0A068RW61|AcoryHHK3|1863-1983
RILLAEDNMVNQKLALRILQKFGHNVTTVSNGKFAVEKFQNEPFDMILMDVQMPVMGGFE
ATQRIRQLEKESGTDSHIPIIALTAHAMIGDREKCLASGMDEYVTKPLRLPELIAAIKKF
A

>tr|A0A068RHK7|AcoryHHK4|1558-1678
EILLAEDNIVNQKLAVRILEKFGHQVKIVSNGQLAVEAYQSHAFDLILMDVQMPVMGGFE
ATQKIREIERMSGTDAHIPIIALTAHAMIGDREKCLNAGMDEYVTKPLRFPELISAIKKF
A

>tr|A0A068S6A8|AcoryHHK5|726-846
RILLAEDNVVNQKLALRILQKVGHNVKIVSNGKLAVEAFETQSFDMILMDVQMPVMGGFE
ATQLIRHIEHESGTHSRIPIIALTAHAMIGDREKCLDAGMDEYVTKPLRLPELIAAIKKF
A

>tr|Q4WI04|AfumiHK9|1146-1265
DILLAEDNDVNQKLAVKILEKHNHNVSVVGNGLEAVEAVKKRRYDVILMDVQMPVMGGFE
ATGKIREYEREAGLSRTPIIALTAHAMLGDREKCIQAQMDEYLSKPLKQNQMMQTILKCA

>tr|G1XR43|AoligHK1|1108-1227
NLLLAEDNVVNQKLAVKILEKYHHTVEVVENGLQAVDAVQKKRYDCVLMDVQMPVMGGFE
ATAKIREYERDFGLPRTPIVALTAHAMLGDREKCIQAQMDEYLSKPLKQNQLIQTISKCA

>tr|J4KR52|BbassNIK1|1073-1192
EILLAEDNTVNQRLAVKILEKYHHVVTVAGNGWEAVEAVKEKKFDVILMDVQMPIMGGFE
ATGKIREYERSMGSHRTPIIALTAHAMMGHREKCIQAQMDEYLSKPLQQNQLIQTILKCA

>tr|M7UGL0|BcineHK1|1109-1228
DILLAEDNIVNQRLAVKILEKYHHVVTVVGNGQEALDAIKEKRYDVILMDVQMPIMGGFE
ATAKIREYERSLGTQRTPIIALTAHAMLGDREKCIQAQMDEYLSKPLKQNHLIQTILKCA

>tr|C5GBV1|BdermDrk1|1091-1210
DILLAEDNDVNQRVAVKILEKCNHGVTVVSNGLQAVEAIKKRRYDVILMDVQMPVMGGFE
ATGKIREYEKKNGLSRTPIIALTAHAMLGDREKCIQAQMDEYLAKPLKQNQMIQTILKCA

>tr|N1J8E8|BgramHHK4|1101-1220
DILLAEDNIVNQRLAVKILEKYHHIVTVVGNGQEAFDAIRAKRYDVILMDVQMPVMGGFE
ATGKIREYERNLGTQRTPIIALTAHAMLGDREKCIQAQMDEYLSKPLKQNHLIQTILKCA

>tr|O74271|CaNIK1|875-999
KILLAEDNLVNQKLAVRILEKQGHSVEVVENGLEAYEAIKRNKYDVVLMDVQMPVMGGFE
ATEKIRQWEKKSNPIDSLTFRTPIIALTAHAMLGDREKSLAKGMDDYVSKPLKPKLLMQT
INKCI

>tr|A8PI69|CcineHHK1|632-756
DILLAEDNLVNQKLAVKILEKYGHSVEIAENGSLAVDAFKARVAQNKPFDIILMDVSMPF
MGGMEATELIRSYEMHKGLTATPIIALTAHAMIGDRERCLQAGMDDHITKPLRRGDLLNA
INKLA

>jgi|Conco1|38317|CcoroHK1
HILLAEDNIVNQKLAVRILEKFGHKTTIVPNGLQAVEAVKNNHYDLVLMDVQMPIMGGCE
ATQHIREWEREHGHHTPIIALTAHAMIGDRKKCIESGMDDYVSKPLRFNELLAAINKCTM

>jgi|Conco1|148367|CcoroHK2
NILLAEDNIVNQKLAVRILEKFGHKATIVSNGLLAVEAVKNNRYDLILMDVQMPIMGGFE
ATQHIREWELESGHRTPIVALTAHAMIGDREKCIASGMDDYVSKPLRFNELMTAINKSVT

>tr|Q6SLC7|CheteNIK1|1120-1239
QILLAEDNAVNQKLAVRILEKYHHRVTVANNGLEAFEHIQKKRYDCVLMDVQMPVMGGFE
ATAKIREWERENGIPSTPVIALTAHAMVGDREKCLAAQMDDYLSKPLRQNQLIQTILRCA

>tr|J3KDN0|CimmiDrk1|1104-1223
DILLAEDNDVNQKVAVKILEKCNHGVTVVSNGLAAVEAVKQHRYDVILMDVQMPIMGGFE
ATGKIREWEKENGLQRTPIIALTAHAMLGDREKCLQAQMDEYLAKPLKQNQMMQTILKCA

>tr|J9W1S0|CneofTCO1|1236-1360
SVLLAEDNLVNQKLAVKLMEVAGHKIEVADNGEIALEKYKRRQLARTPFDVILMDVSMPV
MGGMEATGLIREFEANEGVPRTPIIALTAHAMIGDKERCLAAGMDEYVTKPLRRGDLLAS
IAKVL

>tr|C4R9I5|CpurpHK1|1107-1226
EILLAEDNTVNQRLAVKILEKYRHVVTVVGNGWEAVEAVKEKKFDVILMDVQMPIMGGFE
ATGKIREYERGMGAHRTPIIALTAHAMTGDREKCIQAQMDEYLSKPLQQNHLIQTILKCA

>tr|Q6SLB2|FvertHK1|1082-1201
EILLAEDNTVNQRLAVKILEKYHHVVTVVGNGWEAVKAVQSKKFDVILMDVQMPIMGGFE
ATGKIREYERGIGSHRTPIIALTAHAMMGDREKCIQAQMDEYLSKPLQQNHLIQTILKCA

>tr|F0UKI6|HcapsDrk1|1092-1211
DILLAEDNDVNQRVAVKILEKCNHGVTVVSNGLQAVEAVKKRRYDVILMDVQMPVMGGFE
ATGKIREYEKSNGLSRTPIIALTAHAMLGDREKCIQAQMDEYLAKPLKQNQMIQTILKCA

>tr|G0RFI1|HjecoNik1|1107-1226
EILLAEDNTVNQKLAVKILEKYHHVVTVVGNGWEAVEAVKQKKFDVILMDVQMPIMGGFE
ATGKIREYERGMGTHRTPIIALTAHAMMGDREKCIQAQMDEYLSKPLQQNQLIQTILKCA

>tr|B0DPL6|LbicoHHK1|1018-1142
DILLAEDNLVNQKLAVKILEKYGHTVEIAENGSLAVDAFKGRVAQAKPFDIILMDVSMPF
MGGMEATELIRSYEMHKGLAPTPIIALTAHAMIGDRERCLQAGMDDHITKPLRRADLLNA
INKLA

>tr|A0A0A1V407|ManisHHK3|1102-1221
EILLAEDNTVNQRLAVKILEKYHHVVTVVGNGWEAVEAVKEKKFDVILMDVQMPIMGGFE
ATGKIRDYERGMGTHRTPIIALTAHAMMGDREKCIQAQMDEYLSKPLQQNHLIQTILKCA

>tr|S2J4F3|McircHHK1|1002-1122
EVLLAEDNDVNQKLAVRILEKFGHHVKVVANGKLAVEAYESQTFDLILMDVQMPVMGGFE
ATQKIREIEHSMNNNSHIPIIALTAHAMIGDREKCLQAGMDEYVTKPLRFPELIAAIKKF
A

>tr|S2K402|McircHHK2|1097-1217
EILLAEDNIVNQKLAVRILEKFGHKVEIVSNGKLAVDAFEANTYDLILMDVQMPIMGGFE
ATQKIREIEHNAGTGSHVPIIALTAHAMIGDREKCLQSGMDEYVTKPLRFPELIAAIKKF
A

>jgi|Morel2|1347040|MelonHK1
HILLAEDNVVNQKLAVRILEKFGHKVTIVANGKTAVEFFSSMHFDLILMDVQMPIMGGFE
ATQEIRRLEMLRRAAGGSAINGNNDHLPIIALTAHAMIGDREKCLAAGMDEYITKPLRVN
ELIATINKFPP

>jgi|Morel2|1278143|MelonHK2
HILLAEDNVVNQKLAVRILEKFGHKVTIVSNGKMAVECYDNKHFDLILMDVQMPIMGGFE
ATQEIRKLERLKGKGDHLPIIALTAHAMIGDREKCLAAGMDEYITKPLRVNELIATINKF
PP

>tr|F9WW69|MgramHHK5|1105-1224
DILLAEDNAVNQKLAVKILTKHNHTVTVANNGLEAFEAIRKKRFDVVLMDVQMPVMGGFE
ATAKIREYERTHELARSPIIALTAHAMLGDREKCIQAQMDEYLSKPLKPNQLIQTILKCA

>tr|F4RNQ7|MlariHHK1|955-1074
KILLAEDNHVNQKVATKLLDMGGHKVEVVDNGARAVEAATKNTYDLVLMDVSMPTMGGLE
ATSLIRRHEEEHHLERVPIIALTAHAMIGDRERCIDAGMDEYVTKPLRKADLDASMSRCV

>tr|F4RAY5|MlariHHK6|1004-1124
KILLAEDNKINQIVARRILASSGLNCLVVVENGQEACDAVKSTQFDIVLMDVSMPVKDGR
EATQDIRKWEQTHSVSPVPIIGVTAHALVGDREKCMKAGMSGVCTKPLLAPELLAAMTKA
I

>tr|G4MUD6|MoryzHik1|1104-1223
EILLAEDNTVNQRLAVKILEKYNHVVTVVSNGAEALEAVKDNKYDVILMDVQMPVMGGFE
ATAKIREYERSLGTQRTPIIALTAHAMMGDREKCIEAQMDEYLSKPLQQNHLIQTILKCA

>tr|Q1K908|NcrasOS1|1087-1206
EILLAEDNTVNQRLAVKILEKYHHVVTVVGNGEEAVEAVKRKKFDVILMDVQMPIMGGFE
ATAKIREYERSLGSQRTPIIALTAHAMMGDREKCIQAQMDEYLSKPLQQNHLIQTILKCA

>tr|C1GJZ3|PbrasDrk1|1092-1211
DILLAEDNDVNQRVAVKILEKCNHDVTVVSNGLQALEAIKQRRFDVILMDVQMPVMGGFE
ATGKIREYEREHGLPRTPIIALTAHAMLGDREKCIQAQMDEYLAKPLKHNQMVQMILKCV

>tr|K5VW01|PchryHHK1|1202-1326
RILIAEDNMVNQKLAIKMLEKYSHTVDIAENGQTAVDSYVSRYEAGEPYDVVLMDLSMPL
MGGMEATQHIRRYESQRSLTHVPIIALTAHAMIGDRERCLQAGMDDHITKPLRRNDLINT
MNKLI

>tr|H6QT55|PgramHK2|693-813
KILLAEDNKINQIVARRLLASSGLKCLEVVENGQEAFEAVQANQYDIVLMDVSMPVKDGR
EATQDIRMWEQTHSVTPVPIIGVTAHALVGDREKCMKAGMSGVCTKPLLAPELLAAMSKA
I

>tr|E3JTC4|PgramHK5|1471-1590
KILLAEDNHVNQKVATKLLDMGGHKVEVVDNGELAVEAATKNTYDLVLMDVSMPTMGGLE
ATSLIRKHEQDNHLERVPIIALTAHAMLGDRERCIDAGMDDYVTKPLRKADLDASMSRCV

>tr|B6QNX8|PmarnHHK2|1128-1247
DILLAEDNEVNQKLAVKILEKCNHGVTVVGNGQEALDAVKNRRFDIILMDVQMPIMGGFE
ATAKIREYERENNLSRTPIIALTAHAMLGDREKCIQAQMDEYLSKPLKQNQMIQTILKCA

>jgi|PleosPC15_2|1052949|PostrHHK1|1018-1142
DILLAEDNLVNQKLAVKILEKYGHTVEIAENGSLAVDAFKGRVAQNRPFDIILMDVSMPF
MGGMEATELIRTYERENGLYATPIIALTAHAMIGDRERCLQAGMDDHITKPLRRADLLNG
INRLA

>tr|A0A067N7F8|PostrHHK6|1018-1142
DILLAEDNLVNQKLAVKILEKYGHNVEIAENGSLAVDAFKSRVTQNRPFDIILMDVSMPF
MGGMEATELIRSYEMHTGLSPTPIIALTAHAMIGDRERCLQAGMDDHITKPLRRGDLLNA
INKLA

>tr|B8P856|PplacHHK1|1006-1130
DILIAEDNLVNQKLAVKILEKYGHQVEIAENGSLAVDAFKARIQRNRPFDIILMDVSMPF
MGGMEATELIRAYETSHGLDPVPIIALTAHAMIGDRERCLQAGMDDHITKPLRRSDLINA
INKLA

>jgi|Rambr1|162570|RbrevHK1
HILLAEDNTVNQKLAVRILQQHQNSVTVVQNGKEALECIIAGRYFDMILMDVQMPIMGGY
EATQKIREWERRNIHIDDRHIPIVAVTAHAMKGDREKSLAAGMDEYITKPLRADALMQLI
RQFYE

>tr|I1BQ06|RdeleHHK1|2223-2343
EVLLAEDNDVNQKLAVRILEKFGHHVSVVANGKLAVEAYESQYFDLILMDVQMPVMGGFE
ATQKIREIEKASASNRHIPIIALTAHAMIGDREKCLEAGMDEYVTKPLRFPELIAAIKKF
A

>tr|I1CME0|RdeleHHK2|1691-1811
EILLAEDNIVNQKLALRILEKFGHNVEIVSNGRLAVEAFESRSFDLILMDVQMPVMGGFE
ATQKIREIEHLSGGNSHVPIIALTAHAMIGDREKCLQSGMDEYVTKPLRFPELIGAIKKF
A

>tr|I1BN05|RdeleHHK3|1866-1986
DILLAEDNIVNQKLAVRILEKFGHKVEIVSNGKMAVEAFENRNYDMILMDVQMPIMGGFE
ATQKIREIENISGCHTRVPIIALTAHAMIGDREKCLQSGMDEYVTKPLRFPELIAAIKKF
A

>tr|I1CDG8|RdeleHHK4|2665-2784
EVLLAEDNDVNQKLAVRILEKFGHHVTVVANGKLAVEAYESQYFDLILMDVQMPVMGGFE
ATQKIREIEKTSSNRHIPIIALTAHAMIGDREKCLEAGMDEYVTKPLRFPELIGAIKKFA

>jgi|Gloin1|93283|RirreHK1
DILMAEDNIVNQKLAVKILEKFGHRVEIVSNGQLAVEAFKAKRYDLILMDVQMPIMGGFE
ATQKIREFEQETGGHVPIIALTAHAMIGDREKCLFAGMDEYVTKPLRMNDLIATINKFPV

>jgi|Synplu1|619180|SplumHK2
HILLAEDNIVNQKLAVRILEKFGHRVSIVSNGQMAVEAVQKDTYDLILMDVQMPIMGGFE
ATQRIREWERCTEVIHPIPIIALTAHAMIGDREKCMAAGMDEYITKPLRFNELIATINKF
VI

>jgi|Sporo1|209|SroseHHK1|921-1040
KVLLAEDNIVNQKVALKFLESAGHQTEVVENGALALEAVKKNFYDIVLMDLSMPFMGGME
ATQIIRKFEEQNGLERLPIVALTAHAMLGDREKCIQAGMDDYLTKPLRKPDLLAIINKIV

>tr|U7PS27|SscheDrk1|1141-1261
EILLAEDNTVNQRLAVKILEKYHHVVTVVGNGEEAVEAVKRRNFDVILMDVQMPIMGGFE
ATSKIREYERNLGGTQRTPIIALTAHAMMGDREKCIQAQMDEYLSKPLQQNHLIETILKC
A

>tr|K1WTP2|TasahNIK1|1150-1274
AILLAEDNVVNQKLAVKLLEVAGHKIEVADNGEIAIEKYKRRQMERKPFDVILMDVSMPV
MGGMEATGLIREYEAVSGVAPTPIIALTAHAMIGDKERCLAAGMTAYVTKPLRRGDLLAA
IAKVL

>tr|D5GKW4|TmelaHK3|962-1084
HVLFIDKNRENADIPEMLKMLDLKYHHTVQVVENGLQALEAVKNKRYDVILMDVQMPVMG
GFEATAKIREYEKDAGLPRTPIVALTAHAMLGDREKCIQAQMDEYLSKPLKQNTLIQVIL
KCA

>jgi|Usnflo1|820304|UflorHK8
DILLAEDNEVNQRLAVKILEKYHHGVTVANNGQEALDAVKMKRYDVILMDVQMPVMGGFE
ATKEIRAWERAEGLSRTPIIALTAHAMMGDREKCLQAQMDEYLTKPLKQNQMMQTILKCA
T

>tr|A0A0D1CS93|UmaydHK1|1227-1346
DILLAEDNVVNQKLACKILTNQGHKVDIVDNGHLAVMAVKKRQYDVILMDVSMPVMGGIE
ATMAIREFEKTLGDEQVPIVALTAHAMLGDKEKCLQAGMSAYVSKPIRRVELISTLHSLL

>jgi|Umbra1|243181|UramaHK1
TILLAEDNIVNQKLATRILEKFGHKCEIVSNGKLAVEAFQNKRFDLILMDVQMPIMGGFE
ATQKIREIEQNAATGERIPIIALTAHAMIGDREKCLSIGMDEYVTKVNIHISEGGKNDSK
LT

>jgi|Umbra1|242096|UramaHK2
EILLAEDNIVNQKLAVRILEKFGHKVKIVSNGKMAVDEFGDRPYDLILMDVQMPIMGGFE
ATQKIREIERSAGLGQHIPIIALTAHAMIGDREKCLSAGMDEYVTKPLR

>jgi|Xylhe1|211628|XheveHK6
DVLLAEDNVVNQRLAVKILQKYHHVVTVVGNGLDAFEAVKKKRYDVILMDVQMPIMGGFE
ATAKIREWEKEEGLPRSPIIALTAHAMLGDREKCIQAQMDEYLSKPLKQNQLIQTILKCA
S

Group IV HHKs (n = 12)

>tr|Q4WMD9|AfumiHK1|589-706
HILLVEDNMVNQIVMLKLLKSLGFERVDTAWDGADAVRQVKQTPLSYNVILMDINMPVMN
GLEATTKIREVNSEVPIIALTGNALKGDAETYLARGMNDYVAKPVHRKRLVQLLWKWL

>tr|C5GDW9|BdermFos1|561-679
QLMLVEDNKISQAVMLKLLNNLGFERIDAAYDGAQALKLLRAKPFSYHAVLMDINMPVLD
GVKTTMEIRNTLKLDVPIIAMTANALKGDAESYLSKGMDGYIAKPVNRNLLVKNLLLVL

>jgi|Catan1|397421|CanguHK5
RVLVVDDNPLNRRLLVATLTRLGHATEQACDGKEAVDRVLHDDKPQLDCVFMDFRMPLMD
GLEATRRIVHHYSTHDSKQRRRPIIIGLSADAMTEQEDEGLLNGMDKYLRKPVLKKDLFQ

>jgi|Catan1|156475|CanguHK6
RVLVVDDNPLNRKLMQAMVAKLGHEVELAENGKVAVDRVLAMPAASIGDPAADGARVEAD
EDDTGVANVDCIFMDVRMPVLDGLEATRLIVAHFDSLPDPAQRARRPVIFGLSADALQES
EETGLFHGMDVYLRKPLLRKDIETALNQYFG

>tr|J3KGI2|CimmiFos1|638-755
QLLLVEDNMVNQVVMLKILKSLNFERVDAAWDGAEAVHLIKKKPLAYHAVLMDVSMPVMD
GLEATAAIREMRNEVPIIAVTGNALKGDFETYLAKGMNDFVAKPIHRKELARVLLQWV

>tr|Q6SLA4|FvertHK10|558-675
RILAAEDNIINQKVLVGMLHGFGFKEITVVSDGAQAVSSLSAAADTFDLILMDISMPVMN
GYEATLRIRRSSIRLPIIAMTAYALKGDMERCLEKGMDDYIPKPMDRQVLMRKLLKWL

>tr|G0R843|HjecoHHK4|599-716
RILAAEDNLVNQKVLARVLKSLGFHNTTIAANGAEAVSTLKASPNTYDLVLMDISMPVMD
GFEATRKIRTQGIPIPIIAMTAYALRGDSETCLEKGMDDYISKPVNINKLLQKLLHWL

>tr|E9FDL8|ManisTCS1|561-678
RILVVEDNIINQKVLVGMLHSFGLNNIAVASDGAQAAAMVNQGQGEFDMVLMDVSMPVMD
GFEATASIRRLGSSVPIVAMTANALHGYREKCIRCGMDDYVPKPVGRNVLLQKLLLWL

>tr|Q7RZ05|NcrasHHK8|651-768
SILLVEDNVINQKVMLGMLRSLGFRSIDLAADGAAAVKMVTGKPVGYDIVLMDINMPILD
GNEASQKIRDAGVKVPIIAMTAYALKGDREKCLEYGMNDYVPKPVDRKFLIKVLATWI

>tr|C1GIQ3|PbrasFos1|573-691
QLLLVEDNKISQAVMLKLLKNLGFGRVDAAYDGAQALKMLRAKPFSYHAVLMDINMPVMD
GVEATVEIRNSLKLDIPIIAMTANALQGDAEMYLSKGMDGYVSKPVNRNHLIKNLVRVL

>tr|A0A075AS81|RalloHK3|745-859
KVLVAEDNDINQTIMRSFLLKFGINQVQMAGDGVEALELFKRSKYDLVLMDLMMPRMGGV
EAAKLIREYELLKQLEKTIIIAVTADGHSKFRTPLCFIRFSYSFGFESSKRQNAL

>tr|U7Q251|SscheFos1|779-898
RILVAEDNTTNQRILLRILASFGFDTDCVTVASDGSQAVKHVIERSKDFDLCFMDISMPV
MDGHEATVQIRKNGIQLPIIAMTAYALKGDREQCLAFGMDDYVSKPVNKRLLVDKILTWL

Group V HHKs (n = 41)

>tr|A0A068SGI6|AcoryHHK15|1526-1648
RVMLVEDNLVNQKVLTRYLVRVGLDVEVASDGQECVDMFLSHPHGYYSIILCDLFMPVKD
GYEATRDIRRWEHENSQESIPIVALSANVMSDVAQKCMDCGFSTYISKPVNFATLSDVIR
KHL

>tr|Q4WYC3|AfumiHK12|1788-1910
RILLVEDDETNRMVMLKYLDKIKVMAETATNGQECTELVFSKEPGYYSLIICDIQMPVKN
GYETCREIRRWEMKNHYPQIPIMALSANAMTDQIENAARAGFNDYVTKPIKHNELGKMMM
GLL

>tr|G1XLY9|AoligHK5|1741-1814
KVLLVEDNPVNQKVLLRFLGRVGVDVETATDGEECVAKVLEKGVGWYSLILVSISPPLLP
EYFKGVCIHNLIGD

>tr|J4VV14|BbassHHK2|2103-2225
KVLVVEDNKINQKVLTKYLEKIGVEVEIAEDGIECTDLVFNKPHNYYSLILCDIQMPHKD
GYETCRDIREWEAKNGFDRLPVIALSANVMSVVYDKCTAAGFSDYLTKPVDFISLSKAMV
KYF

>tr|A0A384JGA5|BcineHK2|1988-2110
KVLLVEDNLVNQKVLLKFLSKVGIAVELAMDGVECTEKVFSKPHGFYSLILCDLHMPRKD
GYQTCREIREWESQGSTTKMPIIALSANVMADVLEKCVQAGFNSYVTKPVDFKELSKAMS
SLL

>tr|C5GA53|BdermHHK2|1804-1926
RILLVEDDETNRTVMLKYLEKVKLASETAGNGQECIDMVFSKEPGYYSLIICDIQMPVKN
GYETCQEIRSWEQRNHFPQIPIMALSANAMMDQIDDASRAGFNDYVTKPIKHNELGKMMM
ELL

>tr|N1J706|BgramHHK1|1937-2059
RVLLVEDNATNQKVLLKYLGKVGIAVDLALDGLECTEKVFSKPRSYYSLVLCDLHMPNKD
GYQACREIREWEKKQNYNSMPIIALSANVMTDVIDRCNKAGFSNYITKPVDFKVLSTIMG
DCL

>tr|A0A137PEE5|CcoroHK4|615-733
QVLIAEDNLINQKIMKKFLGKIPNIKVTMVENGLEALSSYHEHPNNHYCLILLDHMMPVM
NGDLVCQLIRENNPSIPIVSVSASTLNNELENFKRVGMSDHLAKPFTSKQLESLISKWL

>tr|A0A137PEG2|CcoroHK5|634-752
EVLVAEDNPINQKIIKKFLGKIPNVKITMVENGLEALNAYHDHPNSHYCLILLDHMMPVM
NGDLVCQLIREKNSTLPIISVSASTMNNELENFRRVGMSDHLAKPFTAKQLETLIRKWL

>tr|A0A137PFY6|CcoroHK6|316-434
FILIAEDNPVNQIIIKKYISKLSTVGFVLVSDGKLAWEEYQKHPAGYFDAILLDHLMPEM
GGDEVCKLIKEKDPSQVIISVSANALPTDIEYFKQIGMDDFLEKPFTFPKFKTLLQKWL

>tr|Q6SLE5|CheteHHK2|1876-1998
KVLLVEDNKINQTVILKFLARIDIATETVLDGVQATEAIFSKPAGYYSIVLCDLHMPNKD
GYQACKEIRRWEKKHGYRRHPIIALSANVLGDVYAKCAEAGFNSYVTKPVEFKELSIAMT
TFL

>tr|J3KBC0|CimmiHHK2|1693-1815
RVLLVEDDETNRAVMLKYLEKVKLRSEVAGNGQECIDMVFSKEPGYYSLIICDIQMPVKD

GYETCREIRSWEAKNHFPQIPIMALSANAMTDQIDDAARAGFNDYVTKPIKHNELGQMMM
ALL

>tr|M1WCE7|CpurpHK3|2047-2169
RVLLVEDNPVNQKVLDKYLKKIVVDVELAVDGAECVEIVLSKPHKYYSLILCDLHMPRKD
GYQACREIRQWEQANGLPAIPMIALSANVMSDVHEKCVAAGFSDYVTKPVDFIDLSRAMA
NFF

>tr|Q6SLA1|FvertHK3|1950-2072
KVLLVEDNPVNQKVLMKYLKKIGVDVEIAVDGAECTNMVLSKPHKHYSLILCDLHMPRKD
GYQACREIRQWEIANNFPPMPIIALSANVMSDVQDKCVAAGFSDYVTKPVDFIDLSRAMS
KFF

>tr|A0A139B0R1|GprolHK5|614-736
RILLAEDNRINQIVAIKFLEKVGLSAEIAVNGREAVDTVIEGARKGEPFDIILMDVMMPT
MSGIDAAEEIARRRDELPRRPHIIALTASASEGDIRKCLVYMQDFLSKPVLLERLVELLE
RVA

>tr|A0A139AVG9|GprolHK6|1211-1332
RILIAEDNEINQRVVVKLLSKLALSSEIASDGREAVDSALRAVQEGKPFDVILMDIMMPR
MSGIEATHAIRAFPPEYPQPHIIALTANASERDVSSYLKIMEGFVSKPIQFENFVTTLEH
VV

>tr|A0A139AF55|GprolHK7|622-743
RILLAEDNKVNALVVMKFLSKVGLSAETAVDGREAVQKALDAASAGKHFDVILMDIQMPV
LDGMEASEAIQRSLSPQQRPFIVALSAAAAPSNVEKCLRIMDLFLAKPVRLEDLVATLET

>tr|A0A139AE29|GprolHK8|560-682
RVLLAEDNPVSRMVATKFLGKLGVRADDAHDGQEAVDMCQRGNVDGKGYEVVLMDGQMPK
LTGLEAAHAIRALLAPLEQPYIIALTANVTEGDIAQARSAGIREHLGKPVSLEDLTKALE
RAV

>tr|A0A138ZZ94|GprolHK9|568-692
RILSVEDNEINQALMRKFLGSMGVQAVAAMNGKEAVELVAKAADKNQHFNLVLMDLQMPI
LSGLEATYAIRNTLGIPPDKQPYIIAVSANAYEHDIVQGRNAGMEDFVAKPTILGALRQA
LEKAI

FA>tr|A0A139A877|GprolHK10|462-584
RVLLTEDNPMYRMVATKFLAKLGVRPDNAHDGEEAFDMWERGCGDGKGYGVVLMDGQMPK
LPGLEAARIIRSLLPHLQQPYIIALTANVTEGDIAQALCAGIQEHLGEPVTLEDLMRVPE
RAV

>tr|A0A139AZL0|GprolHK11|828-965
QLLLAEDNLVNQTLVARLLEKSGFRNVEMCLNGAEAVKTVSERGKNGIPIDLILMFFGID
AGGGDVDTFVDIQMPLMSGYEATKLIRKMDLTVQPWILALTANAQNGDKKRSIEHGMDGH
LSKPLELVDLVSAIDAFG

>tr|A0A139AZS0|GprolHK12|862-985
RILVAEDNPVNQKLMIRLLDRLSFMADLAVNGTEAVQKAKQCALESNPYDLILMDLQMPE
TDGLSATRLIRANPLLTACQPVIVGVTANASVSDRDECLAAGMDGYITKPLKLEQLVDKI
RICI

>tr|F0UAC2|HcapsHHK3|1775-1897
RILLVEDDETNRTVMLKYLDKVKLASETAGNGQECIDMVFSKEPGYYSLIICDIQMPIKN

GYETCQEIRSWEQRNHFPQIPIMALSANAMMDQIDDASRAGFNDYVTKPIKHNELGKMMM
ELL

>tr|G0RJP1|HjecoHHK5|1798-1920
RVLLVEDNTVNQKVMTKFLMKVAVDVDIAVDGAECVERVFSKPHNYYSLILCDLHMPRKD
GYQACREIREWEMHYNYPQLPIIALSANVMSDVQEKCAAAGFSDYVTKPVDFIDLSRAMS
RFF

>tr|A0A014P6C9|ManisHHK2|1965-2087
RVLLVEDNPVNQKVLNKYLKKIGVEVEVAVDGVECTEIVLSKPHSYYSLILCDLHMPRKD
GYQACREIREWEKLSSLPKLPIIALSANVMSDVQEKCVAAGFSDYVTKPVDFIDLSRAMA
KFF

>tr|S2K5K5|McircHHK6|1751-1876
RVLLVEDNLVNQKVLTRYLTRVGLNVDVAVHGGECIELFHKHPKDYYCLILCDLFMPVKD
GYETTREIREWEKQHLAPNEKPKPIVALSANVMSDVANKCLECGFSTYISKPVNFATLSD
VIRGYL

>jgi|Morel2|123498|MelonHK9
RILLAEDDLTNQKVIRRYFQMVGAELVIANDGNECLDKFKSQPKGHFSLILCDLFMPGKD
GYEATRAIRQWEDEHLEDGEQRIPIVALSANVMANVAEQCLSSGFTSYLSKPVDFKRLSE
TLRKLVL

>tr|F9XDR5|MgramHHK2|1468-1590
RVLLVEDNKVNQMVILKFLAKVSIKADTALDGVQCTDKVFAKPHGTYSIILCDLHMPNKD
GYQTCKEIRKWERKNKHPHLPIIALSANVLGDVYQKCVDAGFNSYMTKPVDFKELSQVLM
SFM

>tr|G4MKP6|MoryzHik5|1871-1994
RVLLVEDNPINQKVLIKYLKKVGVAVEVARDGEECTEMVFGHGPGYFALILCDLHMPKKD
GYTACREVRQWEQQTPDGPKLPIIALSANVMADVQERCIQAGFDAYVTKPVDFVDLSNTL
ARFF

>tr|V5IR18|NcrasNIK2|2053-2177
KVLLVEDNLVNQKVLQKYLKKVGVDVELVADGVECTDVVFSRPHDYFSLILCDLHMPRKD
GYQACREIREWEAKGNFPGRALPIIALSANVMSDVQDKCLQAGFNDYVTKPVDFINLSNA
LSRFF

>tr|C1FYM7|PbrasHHK2|1788-1910
RVLLVEDDETNRTVMLKYLEKVKLVSETASNGQECVDMVFSKEPGYYSLIICDIQMPIKN
GYETCQEIRSWEQRNHFPQIPIMALSANAMTDQIDSASRAGFNDYVTKPIKHNELGKMLM
ELL

>tr|B6Q5B7|PmarnHHK3|1746-1868
RILLVEDDETNRMVMLKYLDKVKVVSETASNGQECLDMVLSHEPGYYSLIICDIQMPIKN
GYETCREIRAWEAKNHFHQIPIMALSANAMTDQIDDAARAGFNDYVTKPIKHNELGKMMM
SLL

>tr|I1C0W4|RdeleHHK10
RVLIVSQWEMSRESTVKHVDSILSNIITKNNGEQQILLLTKSICSSLQQQQANVLVITTP
MQRSAITESAKNKEDEVIPKNCGFVFKPLKQTKLQWYFGTRKQ

>tr|I1BV17|RdeleHHK11|1440-1567
RVLLVEDNLVNQKVLTRYLARVGLSVDVAVHGGECIELYMKHPKNYYHLILCDLFMPVKD

GYETTREIREWEKENLGKDEKSIPIIALSANNPDKPLEKPVLQTYKVDLNACGPMVLDAL
IKIKNEED

>sp|Q9P7Q7|SpombMak1|1507-1629
SVLLAEDNIINIKVISRYLERIGVKFKVTMDGLQCVEEWKREKPNFYSLILMDLQMPVMD
GYQACNEIRKYELENDYPKVPIVALSANALPHVVLSCKDSGFDSYLAKPITLQHLSLIIS
GIL

>tr|U7PKW3|SscheHHK2|2144-2267
KVLLVEDNMVNQKVLTKYLRKVGVDVDMAADGEECTDTVFARPHDYYSLILCDLHMPRKD
GYQACREIRGWEAKGNYKKPIPIIALSANVMSDVYDKCIEAGFSKYITKPVDFIVLSRAL
AEFF

>tr|R4X9N1|TdefoHK3|1353-1474
RVLLVEDNPVNQKVMTKYCKKAGLDVDTADDGEQCLKLYDANPALYNIILMDLHMPNLDG
YQACARIRESERKSDRSAMPIIALSANVMSDVADRCKLAGFTSYLSKPVAFNTLSTRISE
LI

>tr|D5GEY7|TmelaHK6|1832-1954
RVLLVEDNRVNQKVLLRFLARVGLEVETASDGEECVERVFEHEPGHYGLILCDLHMPRKD
GFQATKEIRLWEQEHKAHRVPIVALSANVMSDVADRCIAAGFSRYVSKPVDFKELSSTIK
DLL

>jgi|Umbra1|253934|UramaHK8
RVLLVEDNLVNQKVMVRYLVRVGLEVDVVSDGSQCVDAVLSRPHNYYALILCDLFMPVKD
GYEATKEIREWEAKTLHTNAYPIPIVALSANVMSDVAEKCKQVGFSNYISKPVNFTTLSN
VVRGYIQ

>jgi|Umbra1|229472|UramaHK11
RILIAEDDRINQQVLQKFCQGLGYPNVTVVGDGRSAVKKCNKRHFDIVLMDKSMPIMGGM
DAVQAIRDGQAGDKDLHIILISGDTNIDYARYRDLNINGHLVKPIRMPDLKRELDRYSA

>jgi|Xylhe1|244028|XheveHK10
RILLVEDNEVNQLVLQKFFRRISVVVETVSDGAQCIERVFSQPHAFYSAIICDLHMPNKD
GYETCREIRQWEDKQHFSEHDLIPIIALSANVMADVVDKCIEAGFNNYVSKPVNFGELSK
VLMDLLN

Group VI HHKs (n = 22)

>tr|A4D9V5|AfumiSln1|961-1081
RVLVAEDNVVNQEVVLRMLRLEEVYDVTVVKDGQEAYDTVKANMEEGKVFDVIFMDIQMP
NLDGLESTRLIRGMGYSAPIVALSAFSEESNIKDCMDSGMDMFISKPIRRPALKQVLNRS
R

>tr|G1XF75|AoligHK6|959-1079
RVLVAEDNKINQEVVIRMLKLEQIENITLADDGVEAVDRIKEAIESNQAFHIVFMDVQMP
NLDGLESTRKIRELGYSAPIIALSALTEESNIKECLECGMNYFLAKPIRRPALKHVLRTY
C

>tr|G1XC90|AoligHK7|1094-1214
KVLVAEDNRVNIQVIKKMLKLEAVSDITLAEDGAVAVSRVKEALAVGKKFDIIFMDIQMP
NMDGLQATKVIREMGYASAICALTAFAEQSNVKECFDAGMDYFLAKPIKKNDLRKVLQLY
G

>tr|G1XR22|AoligHK8|A993-1113
RVLVAEDNPVNQEVVSRMLRLESVYDIVIAKDGQEAVNRVKEALSEGKHFHIILMDVQMP
TLDGIQATSVIRSLGYRAPIVALSAYSTENNIKECYDSGMDYFISKPVKKSQLRTVLKRY

C>tr|J5K510|BbassHHK4|992-1119
KVLVADDNATNIEVVSRMLKLEEVYDVTVAKDGQEAYELVKETMATPPPHTPPHKQFDVI
FMDVQMPNVNGLESTRLIRKIGCVAPIVALTAFSEESNVQECMASGMNEFLSKPIRRPAL
KKVLSKIS

>tr|J5JIT0|BbassHHK5|1034-1154
CVLVADDNATNIEVVRRMLKLEKVLDVCIAKDGREAYEVVKANMERNRKFDLIFMDVQMP
NLDGIQSTKLIRQMGYAAPIVALTAFSEESNRKECIESGMDEFLAKPIRRSALKSVLTKF
A

>tr|A0A384K2E5|BcineHK15|1000-1120
RVLVAEDNLVNQEVVLRMLKLEDVYDVVVAKDGQEAYDIVKQSMEEGKFFNLIFMDIQMP
NLDGLQSTRLIREMGYSAPIVALTAFAEESNVKECYESGMDHFLSKPIRRPALKQVLKKF
A

>tr|O42695|CaSln1|1249-1370
RVLVAEDNSVNQEVISRMLKQEGITNLTMACNGAKAIDFVKESIENNENFDLIFMDVQMP
EVDGLKATKMIRKNLQYNKPIIALTAFADESNVKECLNSGMSGFITKPISKTNIKKVLVE
FL

>tr|Q6SLE2|CheteHHK5|984-1104
KVLVAEDNKTNQEVVLRMLKLEDVYDVTVAKDGQEALDKVKESMERQAPYNLIFMDVQMP
NLDGLQSTRLIRQSGFSAPIVALTAYAEESNVKECLDSGMDFFLSKPIRRPALKHVLKTY
C

>tr|M1WEM7|CpurpHK6|983-1103
RVLVADDNSTNIEVVSRMLKLEKVHDVTVAKDGQEAYDLVKADMEDNQHFDLIFMDVQMP
NLDGLQSTRLIRQMGCVAPIIALTAFSEESNVKECMESGMNEFLSKPIRRTALKKVLTKF
S

>tr|Q6SLB3|FvertHK12|988-1108
RVLVADDNSTNIEVVSRMLKLEDVYDVTIAKDGQEAYDLVKATMEKNQAFDVIFMDVQMP

NLDGLQSTRLIREMGYTSPIVALTAFSEESNVKECIDSGMDEFLAKPIRRPALKQVLKKF
A

>tr|G0RXE5|HjecoHHK6|1000-1120
RVLVADDNATNVEVVSRMLKLEDVYDVAIAKDGQEAYELVKASMEKNQRFDVIFMDIQMP
NVDGLQSTRLIRKMGYVAPIVALTAFSEESNVKECIESGMDEFLSKPIRRPALKKVLKKF
V

>tr|E9EK45|ManisHHK5|990-1110
RVLVADDNSTNIEVVSRMLKLEKVNNVTIAKDGQEAYELVKANMEEDQPFDLIFMDIQMP
NLDGLQSTRLIRQMGYVAPIVALTAFSEESNVKECMESGMDEFLSKPIRRPALKNVLTKF
A

>tr|F9XM85|MgramSln1|947-1069
RILVAEDNKVNQEVIQRMLRLEKIQHITIASDGRIALGHIISSHATPDHPPFDLILMDIQ
MPNMDGLQATRKIRAEGFKNPIVALTAFAEQSNVEECFGAGMDFFLAKPVKRAKLKDVLV
QCC

>tr|G4MV04|MoryzSln1|1002-1122
RVLVADDNSTNVEVVSKMLRLEHVSDVTVASDGQEALEVVKANMENNLYFDLIFMDVQMP
NLDGLQSTRLIRQMGYSAPIVALTAFSEESNVKECMESGMNEFLAKPIRRPALKQVLRKF
A

>tr|Q1K6A7|NcrasSln1|1024-1144
RVLVADDNSTNVEVVSRLLKLEEVYDVTIAKDGQEAYDLVKANMEHNLQFDVIFMDIQMP
NLDGLQSTRLIRQMGYSAPIVALTAFSEESNVKECMESGMDEFLSKPIRRPALKQVLKKF
A

>tr|B6Q1G5|PmarnSln1|987-1107
RVLVAEDNAVNQEVVLRMLKLEDIYDITVVKDGQEAYDTVKAAMSEGNKFDLIFMDIQMP
NLDGLESTRLIRQMGYSAPIVALSAFAEESNIKDCMESGMDMFLSKPIRRPALKQVLKKF
A

>sp|P39928|ScereSln1|1089-1210
KILVVEDNHVNQEVIKRMLNLEGIENIELACDGQEAFDKVKELTSKGENYNMIFMDVQMP
KVDGLLSTKMIRRDLGYTSPIVALTAFADDSNIKECLESGMNGFLSKPIKRPKLKTILTE
FC

>tr|U7Q3R0|SscheSln1|1048-1168
RVLVADDNSTNIEVVSRMLKLEDIYDVTIAKDGQEAYDLVKANMEKNQRFDVIFMDVQMP
NLDGLQSTRLIRKMGYSAPIVALTAFSEESNVKECMESGMDEFLSKPIRRPALKQVLQKF
A

>tr|D5GG06|TmelaHK5|891-1011
RVLVAEDNKVNQEVVLKMLRLEEIYDVTIAKDGQEAVDRIKEALDVGKHFQLVLMDIQMP
NLDGIESTKIIRHLGYDAPIVALSAFAEEGNVKDCLDAGMNYFLAKPIKRPELKQVLKTY
C

>jgi|Usnflo1|809842|UflorHK2
RVLVAEDNKVNQEVVLRMLKLEDIYDVTVAKDGQEAFELVKESMEQRKYFNLIFMDVQMP
NLDGIQSTRLIREMGYSAPIVALTAFAEESNVKECMDSGMDFFLPKPIRRPALKQVLKRY
C

>jgi|Xylhe1|94789|XheveHK5
KVLVAEDNIVNQEVVLRMLKLEDIYDVTVAKDGQEALDLVKESMEKNQLYNLIFMDIQMP
NLDGLQSTRLIRSIGYSAPIVALTAFSEESNVKECMDSGMNYFLAKPIRRPALKQVLKTY
CA

Group VII HHKs (n = 13)

>tr|Q4WNY6|AfumiFhk4|765-928
TFLVAEDNMINRRVLVNMLKRLGYQDIYEACNGKEAVRIMQDVLAAQKPKASSRLSDCDQ
LGDCGCKRMKPVDVVLMDLWMPEMDGYEATSKILRLVDDYQRRTLQEQPRSQANNLLLPL
PTVLAVSADVTDEALGRASKVGIKGYMTKPYKLSDLERLIVEFC

>tr|G1X8V5|AoligHK9|739-865
RILVVEDNNINRNLITKMLRNLGFADIIEAEDGEDAVAAMAAATSFGRPIELILMDLWMP
KMNGIEATEKIFALPEYANGSKKLTVLAVSADASPDAHRDITRVGMQGFLKKPYTIEGLE
QLILDNF

>tr|M7TQZ0|BcineHK14|765-892
NFLVAEDNVINRKFAITALSRLGYSADNITLAFDGADAVRSYQASLSKPSGRYDIILMDI
WMPNMDGYEATKEITKLAEAHGETATIVAVTADITDDCSMRAKEAGMQSFLAKPYRVIDI
ERLIIGNF

>tr|C5GFX3|BdermHHK5|772-959
TFLVAEDNKINRRILVSMLAKLGYNDVYEAFNGKEAVRIMSEIILNDMSPDLEPAPQSPS
GRSVSEMSTDTVTSLIEDCEMSTFSEPPIKPAPTKSKFVDVILMDLWMPDMDGYQATEKI
LDMVGNHRDRLRNENVPDVLLPPSPTVLAVSADVTDEALGRATKVGMEGYMTKPYKLTDL
ERLIVEFC

>tr|Q6SLD0|CheteHHK17|756-881
NILVVDDNAINRRVLVSMLGRLGYKNITTAFNGNDAVETVRRNALGPASATFDVVLMDLW
MPLLDGFQATEVILSMKELTTKPTILAVSADITDAALDKAVKSGMKGFVTKPFVTRDIAR
LLRTYC

>CIMG_09533|CimmiHHK5
TFLVAEDNKINRRILVNMLAKLGYSDVYEAFDGKEAVRIVSEILSSQASSPPPSTPSAPI
STRSRSNSDITLPNTPVNQPPSNHNTLAMDPANPTQTQSPPDSDQEADKTLPSTKDYKAI
DLVLMDLWMPDMDGYEATEKIFALVEEYRERLANIDLNADGHGQTSQPVSLGPPTVFAVS
ADVTDEALDRATRVGMAGYMTKPYKLRDLERLIEEFCS

>tr|F0UA42|HcapsHHK4|637-822
TFLVAEDNKINRRILVSMLAKLGYNDVYEAFNGKEAVRIMSEMILNDVAPDPGPPPQSPS
SRSVSEMSTDTITSLIEDCQMSTFNEPSIKQVPKSKFVDVILMDLWMPDMDGYQATEEIL
DMVGSHRDRMRSANPDVLLPPSPTILAVSADVTDEALGRATKVGMEGYMTKPYKLADLER
LIVEFC

>tr|F9XF34|MgramHHK17|764-894
NFLVAEDNKINRKLLVSMLSKFGYKNILEAHDGTEAVRQMSVARSPAEQVDVVLMDLWMP
LMDGYEATQRILGMDAARLEHGAVVKRPTVLAVTADVTDGALERAAQVGMKGYMTKPYKL
MDLQRLITEYC

>tr|C1G9X7|PbrasHHK5|780-964
TFLVAEDNKINRRILVNMLSKLGYNDVYEAFDGREAVRIMGDILLNDIPSGPTQPQSPSS
RSFSERSTDTTTSLIENCEISLLHEPNNQIPTRPKFVDVILMDLWMPGMDGYQATEKILD
MVENYRVRLKKQKPNILLPPIPTVLAVSADVTDEALSRATMVGMEGYMTKPYKLSDLERL
IVEFC

>tr|B6QHD3|PmarnHHK5|741-930
TFLVAEDNQINRKILVNMLRKLGYRDVYEAYDGREAVRIMQETLLSSYPLPSPMSSPSIQ

GSDSGIEQSHSPDMDNPGNRKKVKPIDVVLMDLWMPEMDGYEATSKIFEMVDEHCHRLAP
INTEPGLVENWDQRSGSDPPSLFNISPKVLAVSADVTDEALNRASQVGIQGYMTKPYKLA
DLERLIVDCC

>tr|D5G5M9|TmelaHK8|751-879
SILVAEDNKINRKLLTSMLRKLGYTDISEACDGQEAVEIMSSPRHPPIDVVLMDLWMPRM
DGYEATKRILESRRRGAKGGNGGVGVTVLGVSADATQAAMVKTSSVGMKGFMSKPYKIVD
LERLIVEFC

>jgi|Usnflo1|879743|UflorHK4
TFLVAEDNRINRKLLVNMLGKLGYTGVHEAYDGAEAVRQMGIDRNARGERPIDVVLMDLW
MPNMDGYEATRQIFAMEKERERRVRDEFDEDEDGDDDGRTRRKRSRGVTVLAVSADVTDT
ALEKARDVGMEGFMTKPYKLLDLERLVLEYCS

>jgi|Xylhe1|237529|XheveHK7
NILVVEDNIINRKLLVNMLSKLGYSKVYEAYNGVEAVRQMSIDRGRTGAIDVVLMDLWMP
VMDGYEATEKILSMHPGAKNSITILAISADGTTEAKERAARVGINGFLTKPYKLLDLERL
IIEHCA

Group VIII HHKs (n = 35)

>tr|Q4WMN9|AfumiFphA|1184-1315
HILVAEDDPTNSAILRKRLEKSAHTVHITENGKECASVFRDNAHSFDAVLMDIQMPIVDG
MGSTKMIREYEALTASPTSVSATCRRQRIPIFAVSASLVEKDRQTYIDAGFDGWIMKPID
FKRVAHLLDGVY

>tr|Q4WA25|AfumiFphB|1218-1348
RVLVAEDDPVNSKIVEKRLSKLGHAVILTGNGEECAAAFRRDSTKVDVVLMDIQMPIVDG
TESTRMIRQFEQEFQPPLSEKASRNGHVPVFAVSASLLEKDAQLYIEAGFDGWIMKPINF
NRLGVLLDSLE

>tr|G1XV20|AoligHK3|850-973
KVMVVEDNWALQNITKLRLEKLGCEAIIAPNGIDALRLLSTKLAEPDFILMDLQMPLMDG
YDTTKNIRSAAEPGLKDFQYIPIIAVTASTLEEDKEKAFKVGMSDYVFKPLGMNVLKELI
AKYG

>tr|G1XTB9|AoligHK10|1200-1331
HVLVAEDDLVNSRIIKKRLEKLGHRVTLTVNGEECAAVFAERGTDFDAVLMDIQMPIMDG
YASTKTIRKSETEGNSDALPDTHKLNGRIPIVAVSASLLEKERQMYIDCGFDGWILKPVD
FKRLNTLMDGIV

>tr|J4WCZ1|BbassPHY1|1237-1368
RALIAEDDPINLKIVRKRLERAGHMVTHAVNGEDCAALFKSCASTVDIILMDMQMPIVDG
LTSTKMIRSMEHESQIEGHSDVAKRNGRVPIIAVSASLVEGQRQMYIDAGFDAWILKPID
FKRLSVLMGGIH

>tr|G2XN17|BcineHK7|1072-1198
SILIAEDNPINSKLLHKRLSKLSHKPEITAEGQSCYDYYTSGNNKVDVILMDLQMPLVDG
TKATRMIRKFERDNLELHQIRRRVPIIAASASLLEEHRFDYIEAGFDGWIMKPINFSRLE
FLLQGLN

>tr|G2YL65|BcineHK8|1329-1469
KVLVAEDDPVNSRIINKRLDKGGHDVYHTINGEECASAYGDNVKKSSATASLADKRFDVI
LMDMQMPIVDGLTSTKMIRSFEKTYLDLSPRAKLNGRVPIFAVSASLVERERQTYIDAGF
DGWVLKPIDFKRLNVLLKGIV

>tr|G2Y9A1|BcineHK13|1052-1180
NVLVAEDNPLNSRLLEERLRKRGHVVTVTINGQACAEAVMATPDGFDIVLMDIQMPILNG
FTSTTLIRSFTTNTLPSPRASLYGRLPIIAVSASLEESKRDEYVTRGFDGWILKPIDFQT
LEGMLASVE

>tr|N1JCD0|BgramHHK8|1134-1265
KILVAEDDPINSKIIHKRLVKSGHEVYHTINGEDCANVFAERGAYFDVILMDLQMPIVDG
LLSTRMIRLFEKANPQRNLSKCAILNGRVPIFAVSASLNECELQTYIEIGFDGWVLKPID
FERLHVILLGTI

>tr|N1JC78|BgramPHY2|916-1047
NILVADDDLNSLRMLEEKLSQMGHSVSVARDGQECHDRFALNPANTDVILMELKMPYVDG
VLSTRMIRIAEKEAKHRGTSTPQSGRHRVPIIAMSTQLDENSRFDYIQSGLDGWILKPID
FQRLDVVLQGLS

>tr|A8NRX2|CcineHHK2|1288-1418
RVLIVEDNDINRVILAKRLTMKGHTVVNTTNGQEGLDMVKRDQAFDVIFMDIQMPILNGF

EATEQIRQVEPQPSMPSRPSCKLNDGRIPIFAVSASLKESQRDELAEHGMDGWILKPIDF
KRLNTILKGWG

>tr|Q6SLC6|ChetePHY1|1259-1342
HVLVAEDDPVNSRIVKKRLEKLGHHVHLTVNGEECASAYCDNSKDIDVVLMDMQMPIVDG
LTSTKMIRSFEKSHSNMYSPRAAL

>tr|J9VUN2|CneofTCO3|1705-1834
RIMVVEDDPINSQILQKRLKMDKHAVVAVTNGQEAVDQLEKDRDIDAILMDIQMPIMDGR
TSAKEIRELEARTPQPDDIEPFKVDGRTPIFAVSASLYEDDRANLAENFDGWLLKPLDFS
RVRAILEGLE

>tr|M1VVU6|CpurpHK10|1302-1434
TVLVAEDDPINMKILRKRLERVGHEVHHTVNGEDCAAVYKEKSFKFDVVLMDMQMPIVDG
LTSAKMIRSTEHSQDHKGHSRLAKNNRHRIPIFAVSASLMEAKKQLYVDAGFDGWILKPI
DFKRLSTLLSGID

>tr|Q6SLB4|FvertHK8|1267-1398
EVLVAEDDPINMKILRKRLERVGHGVHHTANGEDCAAAYRERSKEFDVVLMDMQMPIVDG
LTSTKMIRSMEASGEHQGHSSLANSNYRIPIFAVSASLVEREKQTYVDAGFDGWILKPID
FKRLNTLLAGIS

>jgi|Ganpr1|123888|GprolHK1
RILIAEDNAINQKILQQRLKKDGHDVVITDDGKQCLDVFIQNYESNPFEICIVDLQMPIL
DGFQCCSEIRSFEQSRSSRDSPTRSPISSSPVDLPFKTPQTVPVITPKPVNGTSIVSSSS
DSASPPSLVRKRLPGSTFSLSGRMPSTLSVSSTPWRTPIIACTAQSHASDAEMCQLGGMN
GFIAKPIDFKRLRLILGGVRD

>tr|G0RIQ9|HjecoHHK7|1218-1349
RVLVAEDDPINMKILKKRLERSGNTIQHAVNGQDCAARYNDGSSAFDVVLMDMQMPIVDG
LTSTKLIRAMETSSEHKGHSPLASRCGRIPIFAVSASLVEELKDIYVEAGFDGWILKPID
FKRLATLLRGIY

>tr|E9EPN5|ManisHHK4|1281-1412
NILVAEDDPINMKILQKRLERVGHTVQHTVNGEDCAMVYKDKSPDFDVILMDMQMPIVDG
LTSTKMVRSTEQSSEHKGHSTMADANRRIPIFAVSASLVEENRQVYVDAGFDGWILKPID
FKRLSTLLAGIH

>tr|F9XAU7|MgramPHY1|1289-1424
RVLVAEDDPVNSRIMKKRLEKLGHEVVLTVNGEECSSMYCDNPGQFDIVLMDMQMPIVDG
LTSGKMIRSFETTHPTPSPTPVLSPRAALNGRVPIIAVSASLEERNLQTYIRTGFDGWIL
KPISFPRLSEIMEGIV

>tr|F4S5A3|MlariHHK2|1263-1395
RVMVVEDDAINRVILKKKLTQSGHAVALTVHGQEAVELFSKDAQFDIILMDLQMPICDGL
EATRRIREFEKERTKWDVSLPKSHRINCGVPILAVSASLHERQRAEITDAGMDGWILKPV
DFARLSSLMVGAI

>tr|G4MU11|MoryzHik7|1345-1476
RVLCAEDDPINMKLLRKRLEKAGHVVSHTVNGADCASVYKELPSSFDVILMDMQMPIVDG
LDSTKMIRAFELEEEKKPEGESTTSGRTIPIIAVSASLLESKKSTYVEAGFDGWILKPID
FKRLETLLTGIG

>tr|Q7S6K1|NcrasPhy1|1069-1203
QVLVAEDDPINVKVLRKRLEKAGYKVTHALNGEDCAAVYEDKPVVFDVVLMDMQMPIVDG
LTSTKMIRAFEKTNRDGSGQQLSDIASDHGRVPIFAVSASLVEQEKDTYVDAGFDGWILK
PIDFKRLETLLQGIT

>tr|Q7S5Q3|NcrasPhy2|1013-1142
TVIVADDNMINVQILERRLTKLGHRVLVSRDGQECFNLFASNRSTVDFVLMDLNMPVVDG
FASIRMIRDQEYSHPTPSRVVQCCGRTPIFAVSGMLRRGQEQQCKEAGFDGWMPKPVDMK
RLVRCLAGGL

>tr|K5WHP4|PchryHHK2|1072-1203
RVLVVEDNDINRTILAKRLTLDGHIVVNTTNGQEGYDMVEQDRDFDCVLMDIQMPLLNGY
EATERIRALEQDRKGSPPNRLSYQLNGRLPIFAVSASLQEAQRDEMYSLGIDGWILKPID
FKRLRVILKGVT

>tr|B6Q433|PmarnPhy|1255-1386
HVLVAEDDPVNSKIVQKRLQRAGHSVYLTGNGEECADAYRNSPQAFDAILMDIQMPIVDG
MDSTAMIRKFEQSSHDYQLSDKAKPLERVPIFAVSASLVEKDVQKYIDTGFDGYIMKPID
FKRVNAILSALK

>tr|A0A067NQM3|PostrHHK2|980-1112
RILVVEDNDINRIILSKRLRNDGHAVVDATNGREGLDQVEADREFDLILMDIQMPILNGF
EATQSIRALETEDMGMRRPMRVSHQLNGRLPIFVVSATLTEHQHDELVNYGVDGWILKPI
DFKRLRTIMKGLT

>tr|B8P0K5|PplacHHK2|869-999
RLLIVEDNDINRMILAKRLSLDGHNVVNTTNGQEGVEMLESDWEFDCVLMDLQMPLLNGF
EATQRIRQIEQERQVGLHRISHKLNGRIPIFAVSASLFEDQREELFKLGFDGWILKPIDF
KRLKIILRGVV

>jgi|Sporo1|31263|SroseHHK2
RVLVVEDEMVNRMIIQQRLKKDGHDVVVVEHGGAAVRKFEEDRNFDIILMDLQMPIMGGL
EASQNIRRIERESPVSHSERRQSDLLNTRVPIFAVTASLPERERGTIVSAGLDGWALKPL
AFDRLRELMQGATD

>tr|K1VPR3|TasahHHK2|1732-1861
RVLVVEDDIVNSQILQKRLRMEHHNVIAVTNGQEAVDALSRDWDIDVVLMDIQMPIMDGF
QAADEIRRLESRINTLEGIDPVRVDGRIPIFAVSASLYEHDRGSLSKNFDGWILKPLDFA
RVRSILSGLE

>tr|R4X8Z1|TdefoHK4|1513-1637
NILVAEDDAINRSILKKRLEIDDHRIKLAFHGLECYEAYSKDTKAYDVILMDMQMPVLDG
MGAARKIRELEATCYADRKSPIPIIAVSASLLESQVEEAIAAGIDGWILKPVNFVRLKQF
FASLT

>tr|D5GFS0|TmeleHK9|527-671
RPHSASKSLIYYEILVAEDNPINSKMGHGVVLTVNGEECADLYSKHGQNFDIVLMDIQAS
LFDFRSSMPIMDGGTSATRIRQFEGADSDQCIPTRHRINRRVPIFAVSASLAEERRAEYI
DFGFDGWILKPVNFGRLKLLLDGIR

>jgi|Usnflo1|801184|UflorHK7
QVLVAEDDPINSKIIKKRLEKAGHEVFLTINGEECSSTYGERPAFFDVVLMDMQMPIVDG
LTSTKMIRSFEKSFPDEFLSKRASLNGRVPIFAVSASLIEKNRQVYIDAGFDGWILKPID
FKRLTLLMSGIVE

>tr|A0A0D1DSU0|UmaydHK6|1374-1509
RVLVVEDDPINRMILKKRLGLDGHTTLLAVNGEEGVRQFEQDAKEIDVILMDLQMPICNG
QEACIRIRDLEHKWAERGEQADRPASQILNGRVPILAVSATLVPQMRQEMVDIGMDGWLL
KPIDFARLGALLKGLL

>jgi|Umbra1|252734|UramaHK4
RVLCVEDNKLNQALICRMMQKLKYVYEVADNGQEAIDIYVRANTTKSTLQKDSFDVILMD
LQMPICDGFEATRQILEYQRHTFGAAVIAAPIIAVTAQAMSGDREICLSKGMKGYVSKPI
DFQVLRQLLEGLRC

>jgi|Xylhe1|239968|XheveHK9
NVLVAEDDPVNSKIVKKRLEKLGHEVYLTVNGEECAGTYGEKPGFFDVVLMDMQMPIVDG
LTSTKMIRSFEKTHASNILSARASLNGRVPIFAVSASLVEKDRHIYTEAGFDGWILKPID
FKRLSVLFEGIGN

Group IX HHKs (n = 21)

>tr|G1X703|AoligHK13|850-1028
HILIVEDNLINQQIATKLVKKLGFTASAVSNGQEALDYLIEANLQFPKDAAINIPARRSR
ATYAASIQLQVQQRLEQEDNMTPPLKLEEARNNRTDPTVNGKMFPDLILMDCHMPVLDGY
SATRALRLLPEPLRNVPIIAMTASAIKGDREKCREAGMSDYLSKPVVTANLERMLVKWL

>tr|J4VXM2|BbassHHK6|1105-1231
HILVVEDNAVNQKIATRTIRKLGFQVTAAWNGREALEYVLGASEGRNVKPDIILMDVQMP
IIDGYKCTHLMRHHRPYKGLIHNVPIVAMTASAIQGDREKCKRAGMDDYLAKPVTMSILE
RMLIRWC

>tr|M7TSD9|BcineHK4|1107-1235
KVLVVEDNAINQQIALKTIRKLGFSPSAVWNGKEALDYLLAADSPNPPHPKPDIILMDVQ
MPIIDGYRATHILRHHSPYRYSACDIPIVAMTASAIQGDREKCQKAGMDDYLAKPVKGKT
LEKMLVRWA

>tr|M7U607|BcineKH5|940-1106
IVLVVEDNAINQTIALKTVRNLGFQATAVWNGREALNYLSNPGPSNPRPDIILMDVQMPI
MDGYEATKILRTSKEYEKNLEPDIQPSENTNGLVSARRSNPNGSKMESPASRTQTKTKSK
LKDLPVIAMTASAIQGDQEKCLEAGMDGYLSKPVEKERLEETLLYWG

>tr|Q6SLE1|CheteHHK6|1037-1165
HVLVVEDNHINQQIALKTIKKLQFSVNAVWNGQEALEYLAKEFGPLHPRPDIILMDVQMP
IRDGYSATHAIRTEAPWRNMPEVQGVPIVAMTASAIQGDKEKCVSCGMDDYLAKPVKGKI
LEKMLVKWA

>tr|M1WIV6|CpurpHK4|1120-1246
HILVVEDNPVNQKIAIKTIQKLGFQVAAAWNGKEALDYMLAVLHGQSRKPDMILMDVQMP
IIDGYRCTHILRQHLPYRTVAQDVPIVAMTASAIQGDREKCQKAGMDDYLAKPVTMTILE
RMLVRWC

>tr|Q6SLB5|FvertHK9|1106-1232
HVLVVEDNPINQKIATKTIGKLGFQVTAAWNGKEALEYLAGVHKGIKQKPDIILMDVQMP
IIDGYKCTHLLRHHMPYKPLVEDVPIVAMTASAIQGDREKCTKAGMDDYLAKPVRGVILE
KMLLRWC

>tr|G0RK07|HjecoHHK8|1048-1174
HILVVEDNPVNQKIATRTIGKLGFQVSATWNGKEALDYMIGASKGEKKMPDIILMDVQMP
IIDGYKCTHLLRHHLPYKSLVQHVPIVAMTASAIHGDREKCTKAGMDDYLPKPVTMKVLE
RMLIRWS

>tr|A0A014PQK9|ManisHHK6|1128-1254
HILVVEDNPVNQMIAIKTIQKLGFQVTAAWNGKEALEYLMATSRGQNMKPDMILMDVQMP
IIDGYKCTHLLRHHSPYKTLLQDVPIVAMTASAIHGDREKCNKAGMDDYLAKPVTMNILE
RMLIRWC

>tr|F9XAD0|MgramHHK6|1063-1190
EVLVVEDNPINQQIALKTIRKLGFPVRAVWNGKEALDYLASPSSSQPRPDIILMDVQMPI
MDGYKATYTIRNSPLFTSSPLLQSTPIVAMTASAIQGDREKCQAAGMDDYLAKPVKKPHL
EKMLVRWA

>jgi|Mellp2_3|1816090| MlariHHK4|569-692
WILLAEDNPIMAKITIGLLKKIGLSKVDLVENGKEAVERIKNRIKIYDLVILDLIMPVLD

GF EATKKIRELELKGSDRVSLPIIALSASATRGDRERCTEVQMDDYLTKPIKSDVLEER
VLHLF

>jgi|Mellp2_3|1816084|MlariHHK7
QYWILLAEDNPIMAKITIGLLKKIGLSKVDLVENGKEAVERIKNRIKIYDLVILDLIMPV
LDGFEATKEIRELELKGSDRVSLPIIALSASATRGDRERCTEVQMDDYLTKPIKSDVLEE
RVLHFLA

>tr|G4MYN5|MoryzHik2|1106-1232
HVLLVEDNLIIQQIAIKNIKKLGFKVDAVWNGKEALAYLEGAQDGKNPKPDIILMDVQMP
VLDGYRTVHLLRHHFPYKTYVNDVPIVAMTASAIQGDRERCMQAGMDDYLAKPVKSKLLE
KMLVRWT

>tr|V5IQK0|NcrasHHK9|1094-1220
FILVVEDNEINQQIAIRTIRKLGFQVAAAWNGKEALEYLSKASTGKTRKPDIILMDVQMP
LIDGYLCTHLLRHHAPYRSYVRDVPIVAMTASAIQGDKEKCKRAGMDDYLSKPVKRTTLE
RMLLRWC

>jgi|Sporo1|28709|SroseHHK6|2230-2346
RVLLAEDNKLIREIVVRTLRGMKFHIDAVCDGLECLAQLEKEDYDVILMDGQMPRLDGYE
ATRSIRENPKKRHIRIIALTASAISGDKERCLTSGMDSYLAKPVRANDLQRTIFEQV

>jgi|Sporo1|23664|SroseHHK8
TWILVVDDNELNRSIIARLLTKMGFHVESAANGYDALDMVANKRYDLVLMDHQMDGMDGL
ETTVKMRQSENPDISGLKIIALTASALKGDQEKFIASGADGYLSKPVRSAVLEATILKAL
A

>tr|R4XH04|TdefoHK6|676-795
RILLAEDNDINCKIATSMLRKLGYACTVVKNGQEAVDVIDEQSSFDLVLMDVQMPILDGY
EATALIRKSPNISMRSIPVIALTASAVSGDRERCIAAGMDDYITKPVNKKNLEKKLQKWL

>tr|D5GL95|TmelaHK2|1022-1169
HVLVVEDNDINQQIAIRLIKKLNFSVSAVSNGLEALEFLKEAAPNGDITTSLSSTTSTPL
CPRRPDIILMDVQMPELDGYSATRIIRSGKLYSYPCAPNRHANDVPIVAMTASAIRGDRE
KCQEAGMDDYLAKPVRSATLEKMLLKWC

>jgi|Usnflo1|890636|UflorHK9
HILVVEDNAINQQIALKTVRKFGFSVNAVWNGKEALDYLLEAPSSTHPKPDIILMDCQMP
VLDGYRATHLIRHHSPYSAIASIRTLPIVAMTASAIQGDKEKCTEAGMDDYLAKPVRGKT
LEDMLLKWAV

>tr|A0A0D1CPY9|UmaydHK2|1161-1279
WILVAEDNLINAQIALRTLKKMGFSCKVAKDGLEAIDEVGKRSYDLILMDCHMPNCDGYE
ATKRLRKSDSVDIRTTPIIAMTASAIRGDREKCLAAGMSDYLSKPVKSAALESTLVKWL

>jgi|Xylhe1|66202|XheveHK4
HILVVEDNAINQQIALKTIKKLKFSASAVWNGKQALDYVSQEPTPQHPRPDIILMDVQMP
ILDGYRATHLLRRHAPYMDIPALLRVPVVAMTASAIQGDKEKCQKAGMDDYMAKPVKGKT
LERMLVKWIH

Group X HHKs (n = 57)

>tr|A0A068RTP7|AcoryHHK6|1356-1479
NILVAEDNPVAQKLLCKQLKRYGFNITCANNGAEAIAAWTKRPVGYFKMALFDHHMPKCD
GVEATKTIRRNEKEQNMKKRLPIVALTADIQDSARAICVNAGMDGYLTKPLNEFALLEFV
KQYC

>tr|A0A068SEM7|AcoryHHK7|2147-2270
NILVAEDNPVAQKLLCKQLKRYGFNITCANNGAEAIAAWTKRPVGYFKMALFDHHMPKCD
GVEATKTIRRNEKEQNMKKRLPIVALTADIQDSARAICVNAGMDGYLTKPLNEFALLEFV
KQYC

>tr|A0A068SJ11|AcoryHHK8|1733-1837
HILIAEDNVVAQKLLYKQLKRYGFNVTCANNGAEAIAAWESQPAGYFRIAFFDHHMPKCD
GVEATKTIRKMEKEEHGMKRLPIVALTADIQDSARAICVNAGMDG

>tr|A0A068SDM6|AcoryHHK9|2351-2474
NILVAEDNPVAQKLIYKQLVRFDFNVTCANNGAEAVDIWQKQPEGYFQMGFFDHHMPKCD
GVEATKRIRKMEANENRSIKLPIVALTADIQHSAKDLCMNAGMNDYLTKPLNQSVLVTTL
RRFC

>tr|A0A068SHG4|AcoryHHK10|2282-2405
HILAAEDNPVAQKLLYKQLTRLGFQVECANNGLEAVEAWLKHPPGYFQMGFFDHHMPRCD
GVGATKKIREIEKEHQCKVRLPIVALTADVQQSARETCLNAGMDAYITKPMNQKSLAEAL
RAYC

>tr|A0A068S2X6|AcoryHHK11 Rec1|1916-2038
SILLATPRDATANMLGRLLGDLQLKRTTTDMQHAIAQALQERHDILLLDIPPMPSSYIAQ
QLQSVDDDPECDLHIILLYTPATEGHKVAAEATNSASDRRGRIMKMAKPARRLKLLRVLE
QVL

>tr|0A068S2X6|AcoryHHK11 Rec2|2143-2300
PVLIAEDNMVAQKLLRKQLEKMGFVVESANNGEEAVQLWQQRPKDHFVIGFFDHHMPKCD
GVEATRKIRQLESSNNVESSSSSTTTTTTTDLASCSSSLSSTSSTACPPTPQRLPIVAL
TADVQTTARDVCVNAGMDGYLTKPLIPKDLAVTLRQLC

>tr|A0A068S672|AcoryHHK12|2579-2702
RILVAEDNPVAQKLLVKQLARLGFVVETCNNGFECIKTWTKRGPDYFLLAWIDHHMPGCD
GIEATKKIRQLEAEMHVAEPMPIIALTADVQKTAQQKCLDAGMTDYLVKPLMQKDLAAVL
RRYC

>tr|A0A068S8H8|AcoryHHK13|2142-2278
HILGAEDNPVAQKLLYKQLTRLGFQVQMTNNGLEAVEVWLSRPPGYFKMGFFDHHMPLCD
GVEATRRIRKLEQSRDEGRVTTEHSATTTATTSFPIVALTADIQDSAKHLCMEAGMNGYL
TKPVDTKELIEVLRKHC

>tr|Q4WYC5|AfumiPhkA|2156-2279
RVCIAEDNPINQKIAVKFVTGLNLQCEAYSDGRQAVEALRTRSQEGNPFHVVLMDVQMPT
LDGYNATREIRKDPDPNVNEVLVIAMTASAIEGDREKCLEAGMNNYLPKPVRSTVLSEML
DNYL

>tr|G1XKW0|AoligHK12|536-659
RVLVIDDDEVVRLVFVRHLEGAGCKVKTANNGQKGLEVLEEMAKSDCLPHLVLTDCRMPV

MDGYKFTTKVRTHPSADIRNLPVVAMSAATTDEERAHCIKVGMSRFLSKPAKKEELYEVL
SDCL

>tr|G1X416|AoligHK14|2325-2447
HILLAEDNPLNTRVALQHLKRMGYTAAHAKDGIEVLEMVEVAASENNQYDVILMDVQMPR
SDGIETSRELMKRYPDSQRPTIIALTANATPSDREKCLQAGMLSHIAKPIKPDDLAAALM
STK

>tr|J4KLM7|BbassHHK1|2227-2351
RICIAEDNPINQKIAIQYVKRLGYCNVVAYENGMKAIEGLRAKAKDGQPYHIILMDVMMP
VLDGYEATKLIRKDPIEAVRKVLVIAMTASAIQGDREKCLAAGMNDYLAKPVRADVLRRK
LDAYV

>tr|M7UJR4|BcineHK3|2220-2344
CICIAEDNPINQKIAIQFMQKLGFKIIDAYDNGMAAVEGIRKKAREGTPYHIVLMDVQMP
VLDGYEATKLLRKDPIDAVRGILVIAMTASAIQGDREKCLASGMNDYLAKPVRSGILKKK
LDQYL

>tr|C5GA56|BdermHHK3|2163-2286
RVCIAEDNPINQKIAVTFVRNLGLECEAFSDGQQAVEALRRASAEGNPFHLVLMDVQMPV
LDGYNATRRIREDLDPCVNDVLVIAMTASAIEGDREKCIDAGMNNYLAKPVRSDVLKRML
DQYL

>tr|N1J703|BgramHHK5|2153-2277
RVCIAEDNPVNRKIAVKFMHKLGFKFVDSYENGWEAVEGLRLKYREGQPYHLVLMDVQMP
VLDGYEATKLLRRDPIEEIRKILVIAMTASAIQGDREKCLDSGMNDYLAKPVRSNVLKMK
LDQYL

>sp|Q5AHA0|CaHK1|2340-2466
RILLAEDNLLNYKVCLKHLDKLGYKADHAKDGVVVLDKCKELLEKDEKYDVILMDIQMPR
KDGITATRDLKTLFHTQKKESWLPVIVALTANVAGDDKKRCLEEGMFDFITKPILPDELR
RILTKVG

>tr|D6RJX3|CcineHHK5|2204-2327
NVLIAEDNPIAQNLLVKQLERYQLTVTATSNGEEAIAAWESHPPGYFSVALFDHHMPICD
GVEAAKRLRLLERKRKIPIQLPIAALSADCQESTKNLCLSAGMNTFFSKPLKKNDLLSLL
SMFG

s>tr|Q6SLE6|ChetеHHK1|2165-2288
RVCIAEDNLINQRIAISFVQKLGFKCDAYLDGFKTIDALERASENGRPFHLVLMDVQMPH
CDGYEATKLIRKHPNPEIRNVLIIAMTASAIQGDREKCIESGMNNYLAKPVRAQTLKALL
ESYL

>tr|J3KBB7|CimmiHHK3|2151-2274
RVCIAEDNPINQKIAVNFVHNLGLTSEVFSDGQQAVWALQQRSKEGNPFHVVLMDVQMPV
LDGYDATRKIRENEDPNVNEVLVIAMTASAIEGDREKCIDAGMNNYLAKPVRSDVLRSML
DRYL

>tr|M1WHM7|CpurpHK2|2253-2377
RICIAEDNPINQKIAIQYVQRLGYPKVSAYENGLKALEGLREKAAEGEPYHIVLMDVQMP
VLDGYEATKLIRKDALEAVRQVLVIAMTASAIQGDREKCLAAGMNDYLAKPVRSEVLKRK
LDAYV

>tr|Q6SLB6|FvertHK13|2220-2344
RICVAEDNPINQKIAIQYVQRLGYKGVVAYDNGLKAVEGLRQKAKEGKPYHVVLMDVQMP
VLDGYEATKLIRKDSLDAVRTILVIAMTASAIQGDREKCLAAGMNDYLAKPVRSEILKKK
LDAYL

>tr|F0UAC4|HcapsChk1|2103-2226
RVCIAEDNPINQKIAVTFVKNLGLECEAFSDGQQAVEALRRASGEGNPFHLVLMDVQMPV
LDGYNATRKIREDSDPNVNDVLVIAMTASAIEGDREKCIDAGMNNYLAKPVRSDVLRTML
DRYL

>tr|G0RJN5|HjecoHHK9|2244-2368
RICVAEDNPINQKIAIQYVQRLGYTSVSAYENGLKAVEGLRQKAREGIPYHIVLMDVQMP
VLDGYEATKLIRNDPIDEVRKILVIAMTASAIQGDREKCLAAGMNDYLAKPVRAEVLKRK
LDAYM

>tr|B0CQW5|LbicoHHK4|2213-2336
NVLIAEDNPIAQSLLVKQLERYNLNVTATNNGEEAIAAWEAHNPGYFSVALFDHHMPICD
GVEAAKRLRSLEAKCKTPYTLPIVALSADCQDSTKKLCLSAGMNSFFSKPLRKGEERDSL
SSAG

>tr|E9EXM2|ManisHHK1|2246-2370
RICVAEDNPINQKIAIQYAHRLGYPNVVAYENGLKAIEGLRKKAAEGEPYHIVLMDVQMP
VLDGYEATKLIRKDSIEAVRKVLVIAMTASAIQGDREKCLAAGMNDYLAKPVRSEVLKKK
LDAYV

>tr|S2JKH4|McircHHK3|2139-2260
PVLIAEDNMVAQKLLRKQLEKMGFLVESANNGEDAVKLWRERPPNYFCLGFFDHHMPKCD
GVEATKRIRAFEIGTNSRLPIVALTADIQSSARDICFSAGMDGYLTKPLIPKDLATTLRK
LN

>tr|S2K226|McircHHK4|2437-2560
KILVAEDNFIAQRLIVKQLNRLGFIVEKCNNGFECFDTWRARGPGYFLLAWIDHHMPGCD
GLEATRKIRAFEKEMNWIPALPIIALTADIQLTAQTNCFNAGMNDYVTKPLMQKDLAMIL
RKYC

>tr|S2J6V9|McircHHK5|2211-2334
HILIAEDNPVAQKLLLKQLTRLGFQVECANNGLEAVNAWNNQPDGYFVMAFFDHHMPKCD
GVAATKRIREIEKEEERKTKLPIVALTADVQESAKQICMNAGMDGYLTKPLNQKVLAEAL
RKYC

>jgi|Morel2|684347|MelonHK6
NVLVAEDDFVSQKILEKQLTKLGMNVVIANNGQEAVNQWLAAERGHYTIAIFDHHMPIMD
GLAATRMIRSLEAEHKAEEEERRKLNPNNDDDAAHKDQSTHGEGSASSGIFKPNRNPNRI
PIVGLSADIQHATKEKCIKAGMDEYMTKPLLTQGLAILIQRYCC

>jgi|Morel2|128130|MelonHK7
NVLVAEDDFVSQKILEKQLTKLGMNVMIANNGQEAVNQWLSVERGHYTIAIFDHHMPIMD
GLAATRKVRALEREFAQEQNDGKEPIRIPIVGLSADIQQSTKESCIKAGMDEYMTKPLLT
KGLALLIQRYC

>jgi|Morel2|1837074|MelonHK8
RVLIVDDNSFNRNLLFHQLTKLGVSKIDQAGSGQEAVDTFIPGTHALVLMDLKMPTMGGF
EATALIRKKEVAYWGPVPVQRSTKNWTDASKLETDPWSPERFVAAPEIPERRSNCSGSGE

GEKKNADKAQSEEGKSATVIAVTADWTAEIGEDREKAVTGGFDDVMVKPISLPSLSVLLE
RYMDY

>tr|F9XCV3|MgramHHK1|2158-2281
RVCIAEDNPINQKIAISFVRKLSFRCEAYGDGQQAVDALTRASEEGDPFHLVLMDVQMPV
LDGYNATREIRKHSDPAVRDILVIAMTASAIRGDREKCLEAGMNNYLAKPVRADTLKQML
ESYL

>tr|G4ML57|MoryzHik6|2279-2403
RICIAEDNAINSRIAIQYAHRLGYITVDAYENGQLAVEALRKKAAEGVPYHICLMDVQMP
VLDGYSASKLIREDPVEEVRSILIIAMTASAVAGDRERCLAAGMNDYLAKPVRSDVLRKK
LDAYV

>tr|Q7RWB9|NcrasHHK10|2246-2370
RICIAEDNSINAKIAMQYMHKLGYPNVDTYDNGLKAVEGLREKARQGNPYHIILMDVQMP
VLDGYEATKMLRNDPIDSVRNVLVIAMTASAIQGDREKCLAAGMNDYLAKPVRGELLKRK
LDTYL

>tr|C1FYM9|PbrasHHK3|2163-2286
RVCVAEDNPINQKIAVTFVRNLGLECEAFSDGQQAVEALRYASAEGNPFHLVLMDVQMPV
LDGYNATRRIRQDPDPNVNDILVIAMTASAIEGDREKCIDAGMNNYLAKPVRSDVLKTML
NCYL

>tr|K5WND5|PchryHHK6|2197-2320
NVLIAEDNPVAQKLLIKQLERFDLNVVATFNGEEAIAEWEARGPGYFSVALFDHHMPVCD
GVEACKRIRILENKRRAQTLLPIVALSADCQESTKQLCLSAGMNSFLSKPLKRSDLVQLL
IQFG

>tr|B6Q5B9|PmarnHHK1|2158-2281
RVCIAEDNPINQKIAVKFVRGLGLECEAFSDGQQAVEALCQGSKEGRPFHVVLMDVQMPV
LDGYDATREIRKQTDPNVNQALVIAMTASAIEGDREKCIEAGMNNYLAKPVRSSVLSEML
DQYL

>tr|A0A067NTS4|PostrHHK5|2167-2290
NVLVAEDNPIAQNLIVKQLQRYDLIVYPTSNGEEALNEWQAHPPGFFSVALFDHHMPVCD
GVEAAKRLRFLENKRDVPTMLPIVALSADCQESTKQLCLSAGMNAFFSKPLRKNDLLSLL
SMFP

>tr|B8P999|PplacHHK6|2012-2135
NVLVAEDNPVAQKLLIAQLQRYQINVVATSNGEEAIAEWEKHEPGYFSVALFDHHMPICD
GVEASKRLRIMENKRRVPMVLPIIALSADCQESTKQLCLSAGMNAFFSKPLKKGDLLTLL
SSFG

>jgi|Rozal1_1|5218|RalloHK1
SILLAEDNLVNQRLIFHMLKKIGYKCDLAENGVQVLELLEKKTYNIILMDVQMPVMDGIT
CTRIILKKNLKNRPLIVALTANALSESKERCLDAGMDDFLTKPIKLSELENVLSKIQS

>tr|I1BH88|RdeleHHK5|1842-1963
SVLIAEDNFVARKLLKQQLEKLGFTVEAACDGEEAIELYKQRPPNYFSVAFFDHHMPKCD
GVEATKCIRSLEKKTGCRLPIIALTADIQSSAKQICVDAGMDDYLTKPLIPKNLAATLRQ
LY

>tr|I1C5D2|RdeleHHK6|2069-2190
SVLIAEDNLVARKLLKQQLEKLGFSVEAASDGEEAIQMYKQRPPNYFSLAFFDHHMPKCD

GVEATKRIRSLEKKTGCRLPIVALTADIQASAKQVCVDAGMDDYLTKPLVPKNLAATLRQ
LY

>tr|I1BHX9|RdeleHHK7|2084-2207
KILVAEDNFIAQKLVVKQLGKLGFVVETCNNGFECFDTWKARGPGYFLLAWIDHHMPGCD
GIEATKKIRAYEKKMKYEKPLPIIALTADIQATAQENCIQAGMNDYVTKPLMQKDLAIIL
RKYC

>tr|I1CTJ5|RdeleHHK8|2142-2265
NILIAEDNPVAQKLLFKQLTRLGFHVECANNGLEAVDAWINRPTGYFSMAFFDHHMPKCD
GVAATKRIREIEREEGKDVRLPIVALTADVQESARQICIKAGMDGYLTKPLNQKVLAEAL
RRYC

>tr|I1C4W8|RdeleHHK9|2184-2246
NILIAEDNPVAQKLLLKQLTRLGFHVKCANNGLEAVNEWINNPMGYFSMAFFDHHMPKVR
KYE

>sp|O14002|SpombMak2|2180-2303
YALIAEDNLIARKLLTKQLSNLGFQVHAAVDGVELVKMYEAKQFGFYSVIFADYHMPIRD
GAEAVMDIRAYERENNCSTPIPVIALTADIQKSAKQRCLEVGMNFYLTKPFTQKQLVNAV
REFV

>sp|O74539|SpombMak3|2211-2333
KILIAEDNPIVRMTLKKQLEHLGMDVDAAEDGKETLQIFESHPDNYYQVCFVDYHMPVYD
GLEVTRRMRKIERKHGCAPLPIFALTADMQPTMETQFQEVGITHYLSKPFKKETLIKMLL
QYL

>jgi|Sporo1|28029|SroseHHK7Rec1
RCLVVDTNETSRKALDQLLCSFGLQVTAPADISLAYEIATKAAEDRRPYDFFIVDAFLPS
FGAHALIRRLRGRGLSAPTIALTRMGSPIYEEIRQLDCSFLIKPLKKNRLYHALRTIFP

>jgi|Sporo1|28029|SroseHHK7Rec2
PLAILVAEDNPINVKVITHLLKRMGYTCDVAEDGQVALEKVQKKRYDLVLMDVNMPNMDG
ITSTKKIIELMPERNSRPSIVCLTANAMAEDKARCLASGADSYVSKPILVPDLLRVLEEA
GV

>tr|U7PKV0|SscheHHK4|2319-2443
RVCIAEDNPINRKIAIQYVQRLGYKTADAYDNGQKAVDALREKAKEGVPYHIVLMDVQMP
VLDGYEATKLLRRDPIKDVREVLVIAMTASAIQGDREKCLAAGMNDYLAKPVKSEVLKSK
LDAYI

>tr|D5GCS1|TmelaHK7|1820-1942
NILLAEDNLVNTRVALQHLKRMGYSAKHAKDGIEVLEMCEEAAERGDMFDVILMDIQMPR
ADGIETSLELRRRYVDEERPTIIALTANATANDRERCQQAGMSSHIAKPILPNDLATALM
STS

>jgi|Usnflo1|894318|UflorHK10
RICIAEDNPINQKIAVSFVSKLGFKSEAYNDGLQAVEALRQRSSEKNPFHLVLMDVQMPV
LDGYDATKVIRTDKDPAVRGVLVIAMTASAIQGDKEKCLEAGMNNYLAKPVRAAVLKAML
ENYLKQ

>jgi|Umbra1|237333|UramHK5
RILIAEDNPVAQKLLFKQLSRLGFNVDCANNGLEAVVLWESHPETYYAVALFDHHMPVCD

GVEATKRIRHTEKTSNRKRKLPIIALTADVQQTARDVCSSAGMNGYLTKPLNQNSLAELL
RRYL

>jgi|Umbra1|235482|UramaHK6
NVLIAEDNPVAQKLLVRQLQKLGFAVESVKDGEEAINIFTSSKRDHFSFAIFDHHMPKCD
GPEAAARIRHIEATDNLNRMPIFALTADVRAIARKASEQNGMEDYLTKPLIMERLVAAIR
NHC

>jgi|Umbra1|260316|UramaHK7
KILVAEDNIINQKVIVQLLKRMGFTTDIANDGAEALDMMEATTYDIVFLDLNMPKKDGLT
VAREACEKYPPDVRPTLVAMTANAMRGDREQCLQAGCKDYIAKPILIPELSRVLESCSK

>jgi|Xylhe1|265722|XheveHK1
RICIAEDNPLNQKIALSFVQKLGFKCDAYNDGQQALEALRERAAKGADEAYHLVLMDVQM
PVLDGYEATKLIRQDKMPLVRGVLVIAMTASAIRGDREKCLEAGMNDYLAKPVRAQVLKT
MLEQYLG

Group XI HHKs (n = 47)

>tr|G1X519|AoligHK15|1220-1351
RKFLYMQNLVNQNVIRKQLQKLGCETYVANHGLEALEKVMQSKLYMKATGDAYDLTVILM
DVEMPVMDGLKATGEIRKLEAEGSLVRRIPIIAVTANARPEQIKQMKEAGMNDVLSKPFR
MPELVNKLEGVL

>tr|G1WZ89|AoligHK16|1815-1946
KVLIVEDNLVNQKLLRKALENSGCTTYVANHGVEALEMIKESVYWKGAREDALELSLILM
DVEMPVMDGLTCSKQIRELQAEGSILGHIPIVAVTANARQAQIDRMFAAGMDDVVSKPYR
IKELIPKLHSVV

>tr|G1XIV8|AoligHK17|1045-1164
RVLVVEDNILNQKILRKQLAAQGCETVTANNGEEALELLKAGGKFTIVLMDMEMPVMDGA
TATGLIREYEAGAGGHVPIIGTSANARFEQVQFMINAGMDDVITKPFLILDLMEKIRQVL

>tr|J5K2W1|BbassHHK13|1204-1335
DVLIVEDNIVNQKVLQRQLVNCGNKTFVANHGQEALETLQKSRFWTGNESDGVDISVILM
DLEMPVMDGVTCARRIRALEREGTITKHIPIIAVTAYARPEQIANAKAAGIDDVISKPFR
IPELLPRIDELV

>tr|M7TCK0|BcineHK11|1083-1216
HILIVEDNIVNQKVLSKQLRSAGCIVSVANHGLEALEFLEKSTFSTSLPEGVSGIPCNIL
LMDLEMPIMDGLTCVRQIRKWEREGRVKGRVPVIAVTANARSEQIARAKEAGMDSVVTKP
FRIGELVPEMERWL

>tr|A0A384JYC2|BcineHK12|891-1022
HVLLVEDNLVNQKILEKQLKKAGCIVYVANHGLEALETLRQCSCWHEPVKDAKKLDIILM
DWEMPVMDGLTCSREIRALQQSGKILRHLEIIAITANAREEQVQLALHNGINFVMSKPFI
VSSLLSKMRERL

>tr|M7UAG3|BcineHK18|1414-1547
TILIVEDNLVNQRVLQKQLHRAGFVTQVSNHGLEALHKLEASSFWKNETPTAERTSISCV
LMDLEMPIMDGLTCTKKIRALEADGTIVRHVPIIAVTANARLEQIETALATGMDDVVSKP
FRMPDLIPKVEALV

>tr|Q6SL97|BcineHK19|934-1069
EVLVVEDNLLNQKVLVKSLRKEGYRINVANHGGEALEFLKKTTFWVGESGEEENTKQPLN
LILMDLEMPIMDGITCVRQIRKWEAQGVIRGHVPIIAVTANARKDQIMSTIEAGMDDVTT
KPYRIHDMLRQIETLV

>tr|G2YM64|BcineHK20|1296-1458
SVLIVEDNLVNQKVLAAQMKRLGCTVHVANHGKEAIEFMSKTNWMSEEVHHLNAVVSKKN
TSVPNQTQEPSKLHQESRLQKSPIPLTLILLDIEMPVMDGLTCAREIRKMENNGQLKGHV
PVVAVTANARAEQIQQALEAGMDEVMTKPFRITELVPVIEGVL

>tr|N1J5W2|BgramHHK3|644-774
HVLLVEDNIVNQHVVNKQLSKAGHKVYLANHGGEALEIIRQMDVWHETPEGKHLDIILMD
WEMPIMDGLTCSREIRRLQMEGKINRHIEILATTANARPEQIATALDSGIDSVMVKPFLV
KNLIQRMRERL

>tr|N1JLL0|BgramHHK6|1120-1251
RILIVEDNLVNQRVLQKQLSKKGFKTSLANHGGEALEFLKTTKFWKENEQDGLDLTVILM

DLEMPFMDGLTCSRHIRDAEVEGKIVGHVPIIAVTANARLEQIEAAVVAGVDDVQSKPFL
IRDLLPKIEKYS

>tr|Q6SLD9|CheteHHK8|805-999
NVLIVEDNLVNQKVLRKQLEKFKWNVSVAGNGQEALEWLKNSIYWQQPDSDSPTQAESSK
HDLDIILMDIEMPIMDGLTCARLIRDYEHQGLLASPSPSFSQLRRLSAVSVSPIQSSNHN
DPFSTSLAKHRYSTSSRESNDNNPAHRQFLRLPILAVSANARMEQVDQALAAGMDDAISK
PFRIPELWPKIRGLV

>tr|Q6SLD6|CheteHHK11|1120-1278
HVLIVEDNLVNQRVLAKQLRALNCTISVANHGKEALDFLPKTTLWNHTHPLSSSLSRKEK
YHVPSTEPMPLGYDDDVPAIPLNLILMDWEMPVMNGLKAVAEIRRLEREGLLRGRVPVIG
VTANVREQQIRTAVDAGMDDVVGKPFRVAELLERMRDVV

>tr|Q6SLD5|CheteHHK12|754-885
TLLLVEDNLINQKVLRRQLQSRGFQVFTANNGQEAIDAVAERGSRAQNGPDDRNYFDVIL
MDQEMPIKDGNAATQEIRQLQEEGKAGYSHILGVSANVREAQTQSMRDAGMDDVISKPFK
VDDLVRKVRSLV

>tr|Q6SLD4|CheteHHK13|1303-1440
KVLIVEDNLVNQRVLQRQLQNQGIQTIVANHGGEALEVLKLSTFWASPSSPTTTATPPTD
ISVVLMDKEMPVMDGLQCTSRIRELEQHGKFKCHVPIIAVTANARSEQIATLLAAGMDDV
VSKPFRIGELIPKIEDLA

>tr|Q6SLD3|CheteHHK14|1150-1282
SILVVEDNLINQRVLSAQLKKLGHTVHVANHGVEALSHLSQTPFSATPTASPSVPLSVVL
MDIEMPVMDGLTCTRKIREMEAQGELSGHVPIIAVSANARREQVELAKSAGVDGAICKPF
RVPELIGLIEGLG

>tr|Q6SLD2|CheteHHK15|818-938
RVLLVEDNLINQRVLGKQLKKAGCDVTVANHGLEALDSLDRQTFDIVLMDLEMPVLDGLE
AMRQFRKKELNECRPVRLPIIAVTANVRKEQMDTAMAAGADCVMQKPFKAADLVFMMKSL
I

>tr|Q6SLD1|CheteHHK16|1112-1240
HVLVVEDNHLNQKILCKQLTKAGCITATADNGVHALDYLTKTQFCTPNGIPLSMVLMDCE
MPEMDGLTCCRKIREMEQRKQVLGHVPIIAVTANIRCGQIDEARESGMDDVIGKPFRIPE
LLDKMRGLL

>tr|Q6SLC9|CheteHHK18|1023-1153
HVLIVEDNLVNQRVLAKQLRNTGMHVTVANHGGEAIEHLHTTNYCVSDGSGKPLSLILMD
WEMPVMDGLTCVRNIRDLQRKGVVTAHVPVIAVTANVRSEQVEVALKAGMDNVISKPFRI
PELCACIQKTL

>tr|Q6SLC8|CheteHHK19|1027-1161
SVLLVEDNMVNQRVLKKQLQQHGYIVHTADNGQKAFDFIKTSRHWKAYPDTTASDSYIDV
ILMDVEMPVMNGLQCAKMIRTAQNNGQINKHLQIVAVTANARPEQLKRATEAGMDDAISK
PFRVKDLVRVIDRLG

>tr|M1WC46|CpurpHK5|1091-1222
KILIVEDNLINQKVLNRQLVNKGNKTYTANHGQEALDLLAKSRFWKGRAADGFDISVILM
DLEMPVMDGLTCTKKIRELEREGTIVKHIPIIAVTAYARPEQVQKAQDAGMDEVMPKPFR
MTDLLPKIKDIV

>tr|M1VVM8|CpurpHK8|1168-1299
DVLIVEDNIVNQRVLQRQLRQCGNNTFVANHGFEALQMLQKSRFWAGQEQSGVDISVILM
DLEMPVMDGLTCARKIRQLEMEGTLLAHIPIIAVTAYARPEQIESAKAVGIDDVISKPFR
VAELLPKIEELV

>tr|Q6SLB7|FvertHK14|974-1102
TIHIVEDNLINQKVLVNQLRKAGCTVSATNDGLEALEFLKKTHFFKAGGSDLSVVLMDLE
MPNMDGYQCVREIRRLESEGLIKKHVPVIAVTANVRDEQIRMALDCGMDDLVSKPFRIPE
VMSKIEGLL

>tr|Q6SLB8|FvertHK15|1209-1359
GILVVEDNLINQQITRRGLTDRGYMVDVANHGIEALDKLKRRQGGVPGLGLKAGSSRSGR
SNMNPLPIAINLVLMDIEMPVQDGLTCTRIIRELEAEGEIFCASGGRIPIIAVTANARPE
QVMEAKQAGCDDVMVKPYRIPELIEKMQVVV

>tr|Q6SLB9|FvertHK16|1362-1493
DVLIVEDNIVNQKVLQRQLRNCGNNTFVANHGKEALQTLERSRFWAGKEAEGVDISVILM
DLEMPVMDGMTCARKIRELEREGIIVQHIPIIAVTAYARPEQIESAKAAGIDDVISKPFR
IPELLPKIEELV

>tr|G0RNP4|HjecoHHK10|1209-1340
DVLIVEDNIVNQKVLQRQLRNYGNNTFVANHGREALQTLEKSRFWAGQEDEGVDISVILM
DLEMPVMDGMTCARKIRQLEKEGTIIKHIPIIAVTAYARPEQIENAKAAGIDDVISKPFR
IPELLPKIEELV

>tr|A0A0A1UWS0|ManisHHK13|1356-1487
DVLIVEDNIVNQKVLQRQLRHCGNNTFVANHGFEALQTLQKSRFWAGQEQDGVDISVILM
DLEMPVMDGMTCARKIRELEREGTIVAHIPIIAVTAYARPEQIESAKAAGIDDVISKPFR
IPELLPKIEELV

>tr|F9XQG4|MgramHHK7|820-952
HILLCEDNIVNQRILAKQLRSKGCTVTVANHGQEALEILEKSDWRCDKPAADTSSIDVVL
LDWEMPIMNGLQCCQKIREFETSGEANRRLPVIAITANVRQAQIEEAMAAGMDTVVPKPF
TVTELLDSIENLA

>tr|F9WXL7|MgramHHK8|969-1100
RVLIVEDNLVNQRVLQKQLKNIGTEVHLANHGGEALEKLMKSTYWRGGKGERLELGVVLM
DQEMPVMDGLTCTRKIRELEKEGKLTGHVPIIAVTANARAEQVQTALDAGMDDVVSKPFR
IPELVPKIEELM

>tr|F9X6M1|MgramHHK14|588-719
CILVVEDNLVNQKVLRMQLQKLGHEVHVVNHGGEALEFLKRTTSCEGNESSPIRLSVVLM
DIEMPVMNGLECTQKIREAESDGWLSGRLPIIAVSANARDQQLRSAMDHGVDDAITKPFR
VADLLPKIERLV

>jgi|Mycgr3|107160|MgramHHK18
RVLVVEDNAINQKILANQLRKRSFEVKVASHGEEALSALQMAVERPVPPTINRKPVPGTL
FDVVLMDIEMPVMDGITCVKRIRAYETSRGIPTRLPVIAVTANARSEHGTSAIEAGMDSV
TTKPYKIEELVEEIEKTED

>jgi|Mycgr3|110667|MgramHHK19
HVLIVEDNTINARVMSKQLRRAGCIVHIANHGGECLDFLDRSSFCAPTPPSTPTSPNVPP
TPLSIILLDLEMPTMDGLTCIRHIRERQANGRLVGHVPVIAVTANARSEQIHFAIEAGMD
QVVTKPFRIPELVPRMEGLVG

>jgi|Mycgr3|76299|MgramHHK20
HVLLVEDNVINQRIVFRKLEAKGFNVTTANNGKEAVDAARNAPKGSSGDKGAFDIILMDQ
EMPIMDGNAATKAIRELEERGEVERVPILGVTANVRGAQQEEMLNNGMDDVISKPYMIEE
MVSCIMSIVF

>tr|F9XM35|MgramM3YP1|842-972
HVLLVEDNLVNQKVMAKQLMKAGHVVALANHGREAVEYVQRTKFASGDGAGEELDVVLMD
IEMPIMDGLEATRLIRSMESSGALHGTVPVIAVTANARGEQQMTAREAGVNAIVTKPFQM
AELMHEIHRVA

>tr|F9XBT9|MgramM3YP2|834-963
VVLLVEDNLVNQKVMAKQLRSAGHDVTVANHGQEALDHIRTTHFCVPNGGVELDVVLMDI
EMPIMGGLECTSRIREMEMKGEIQGRVSLIAVTANARAEQQKQAMDVGFDAVITKPFKME
ALRVEMDSVC

>tr|G4N1Z5|MoryzHik4|1207-1334
HVLVVEDNLINQKVLASQLRKAGCTVHTANDGLEALEFLETTAFRVGNVRLSVILMDLEM
PNMDGLTCVSEIRRMEVDGRIETHVPVIAVTANVRDEQVTEARRSGMDDVLSKPFRVPTL
FDKIGALL

>tr|G4MXJ1|MoryzHik8|1191-1361
DVLIVEDNLVNQRVLQRQLVRCGNNTLVANHGQEALATLRRSRFWRHGVIEEEQHQLGGT
GEQSTKPGASMTDDPSTPGTSMTSTGQQETDINISVILMDLEMPVMDGMTCARRIRELEQ
LGVIAHHIPIIAVTAYARPEQIETAKAAGIDDVISKPFRIPELIPKIEELV

>tr|G4NJF1|MoryzHik9|1292-1466
GIMIVEDNLINQQVTRRMLGDRGYAVDVANHGLEALEKLRTSSRFVGNDSDLAKKDNLPN
ISASSHNGIAAAAIAAAVSTTTVEPSSKFELDVILMDIEMPIQDGLACTRAIRELERQGK
IITRPGLGTSRLPIIAVSANARMEQILEAKAAGCDDVLVKPYRMPELMEKMRIVL

>tr|Q7SFA8|NcrasDCC1|1373-1535
DVLIVEDNLVNQRVLQRQLQLSGNNTFVANHGGEALVELRKSRFWNKEIAATDDAASALP
RSNKKAVNDGGTDGSPDGEENSNNNISVILMDLEMPVMDGMSCTREIRRLENVGVITRHI
PIIAVTAYARPEQVESARAAGVDDVISKPFRLPELIPKIEELV

>tr|Q7SEW7|NcrasHHK11|1291-1475
GIMVVEDNLINQQITRRGLSSMGFTVDVANHGLECLDKLQRTDRYVSDQGSGTALPMNGS
INNKGALPALNGTISPLSPPVSGSFLNQSQAPPATTTIATPATKFPLSVILMDIEMPIQD
GLTCTRNIRELERQGKITGGRLPIIAVSANARIEQILEAKEAGCDDVLIKPYRMPELLEK
MRIVM

>tr|U7PVB7|SscheHHK6|1273-1402
GVLLVEDNMVNQKVTRRYFEKSGFHVQVAANGLEAMDIIKASDRRVPGSYPISVVLMDME
MPVQDGLTCTRHIRALEAAGTFAGGRIPVLMITGNARPEQIADARAAGCDDVVVKPFQMH
LLFEHIKLVM

>tr|U7PVL9|SscheHHK7|1503-1679
DVLIVEDNIVNQRVLQRQLDKCGNRTMVANHGGEALEILLQAASFGKANSDSGSAAGDEP
ASSVKGQPEATRVPSALAPASAAMAPSASSSGLRPLGSYVSIVLLDLEMPVMDGMTCARA
IRALERSGTLPGHVPVIAVTAYARSEQIEAALAAGIDDVISKPFRLAQLTPKIQELV

>tr|R4XEF1|TdefoHK7|812-937
IILIAEDNLINQKIMVKQLTNAGYTTLVANNGQEAIDILLEDQSGPERICMCLCDVEMPV

MNGIETIKEVRKLELTKKLRGHCPFIAVTANARQEQVSEMLAAGMDDSMPKPFRFDALIA
KMVALL

>tr|D5GNY8|TmelaHK1|1083-1211
GVLIVEDNLINQEVLRRQLRNQGCVTYVAGNGQEALDFIIKSEFVKDGATKIDVILMDME
MPVMDGNTATVKIRELERSGKIRRHVPIMGISANARPEQVARMTEVGMDDAISKPFRIAD
LLSRFDTLL

>jgi|Usnflo1|901795|UflorHK5
KILIVEDNLVNQKVLGKQLRNMGCIVHVANHGGECLDRLRESTFWKGNARNGLEISVVLM
DQEMPVMDGLTCTKEIRKLEEKGDVVSHVPIIAVTANARSEQIDTAMDVGMDDVVSKPFR
IVDLIPKVEELTG

>jgi|Usnflo1|696185|UflorHK6
RVLLVEDNLVNQKVLSKQLQKAGCTVHVANHGQEALDFLERSNLWHEHPDGEAVDIVLMD
LEMPVMNGITAARRIRELQGQGTLVKHVPLIAVTANARKEQIETSLAAGMDDVMPKPFRV
SELLAKMEALNQ

>jgi|Xylhe1|238996|XheveHK8
KVLIVEDNLVNQRVLQKQLRNIGCVVHVANHGGEALERLKQSKYWRGRESDGTDLTVILM
DLEMPVMDGLTCAKRIRELQSNGSITKHVPIIAVTANTRLEQIDTALAAGMDAVVSKPFR
IPELIPKIEELSA

Group XII Rec1 (n = 15)

>tr|J9VXZ1|CneofTCO2 Rec1|1083-1198
VIMLVEDSADTRRYMKSIFSQYCQVVEARDGQEALELCQKSVPDLIISDVMMPHLNGFEL
LVALKRSKDLKMVPVIMLTARGADESKVDGIMAGAEDYLAKPFSAREIVARAHMQL

>tr|J9VXN3|CneofTCO4 Rec1|952-1067
IVLIVEDNVDMREYIRQLFAPYCTVLEASNGEQAYNMATQNPPNLILSDVLMPKLSGMEL
LQRIRSHPDTRIVPMVLISAIAGDESRVEALLNGADDYLAKPFKPKELIARVHLHM

>jgi|Morel2|153930|MelonHK3 Rec1
YIVVVDDNNDMRAYLREILGKDFRVRCAVDGLDALRVISERLKQGKRIDLVLSDVAMPNM
NGYELLQRLRNDTTTMMTPFILLSARAGEEANVEGLDLGADDCLVKPFSARELLARVRSS
I

>tr|F4RB40|MlariHHK3 Rec1|1256-1371
TILIADDNDDMRSFVRSVLSRYYTVAEASDGLEAYHWAKAHHPDLVVSDVMMPALNGFEL
LKRLKTDPDTAGISVILLSAHAGSEIRIEGLAEGADDYLVKPFEAKELVARINTHL

>tr|E3KZK9|PgramHK1 Rec1|1467-1582
TVLIADDNSDMRAFLKSVLSRYYNVVEAVDGQQAYQLAKLNRPDILVSDVMMPGMDGFEL
LRRLKADSDTAGMSVILLSARAGSESRIEGLAEGADDYLVKPFEAKELVARVNTHL

>jgi|Gloin1|21566|RirreHK10 Rec1
KHVVLLADDNTDMRNYLSGLLKKEFIVHCACDGREALKKLKKLKNPPDLILSDIMMPNMN
GFELLKSIRSDISTQLIPVILLSAKAGEEASIEGLDKGADDYLTKPFSARELIARVRVNI

>jgi|Gloin1|342107|RirreHK14 Rec1
TVLIVDDNTDMRDYIFGVLKKDFDVCCACDGLDALRTLKKLDKPPDLILSDVMMPNMNGY
DLLKSLKNNKSTRLIPVILLSAKVGEEASLEGLEHGADDYLIKPFSAKELIARIRVNIK

>jgi|Sporo1|24426|SroseHHK3 Rec1
DYLGGEDVLSLKNRTIVLVDDSKDLRTYMTSLLSTQFTVIPFANPLDALDYITANPPNLV
VTDAMMPGLSGMQLTSAIRQNPRISFLPVVMVSAQAGLEARAEALEGGLDDYLVKPFQPR
ELMARVKGEYR

>tr|K1VJU5|TasahHHK1 Rec1|555-672
ILLVVDDNEELRTYISHIFSPFCTVIQASGGDEAYKLVKSHRPHLVLSDLLMPASSGMDL
LLAIRSSEDRGIKSTPVIILSAINDDETRLEGLVGGAEEYISKPFKRNELLARVHLHM

>tr|K1VTK5|TasahHHK3 Rec1|1148-1264
VVLVVDEMKDIRDYIKDLFEPYCTVLEASNGYEALELIQAGQKPSIVICDLLMRKLTGLE
LLSELRATAATQFVPVVFLSPVNDDDLRVSAFMAGAEDFIIKPFKPKELLLRVHLHT

>tr|K1W7Q9|TasahHHK4 Rec1|1007-1119
VVLVVDSNAESRAFLRSVFAPYCKVVEATDGKDALERVDGIAPDLIVIDALLPQVSGHDF
VRAMRTGQVMRKTTPIMVLTVNDDRHSNQEGADDYLAKPFNPQELIARASLQM

>tr|K1VSA5|TasahHHK8 Rec1|953-1067
VVLIVDDLLDTRKYIRHIFEPFCTTLEARDGVEALEKVRERVPDLIIADTMLPRMDGVQL
LHALRTGQDHREKLVPVILLTSQGSALSDGAFGADDYISKPFNARELIARAHMQL

>tr|K1VVT9|TasahHHK9 Rec1|351-467
DDGSLSSPMSVRGVDRSTLQFEPTDCVLLVDVFEAKDKDEALRIIERTPPTLVIADTMLP
SDGEGLVATMRRGSRTINTIPVILLTGHERSDAGAADDYLLRPFNARELLLRGHMQM

>tr|K1VBK1|TasahHHK10 Rec1|930-1045
VLLIVDDNRDMREYIKLIFSPFCRVLEAEDGVDALRIMVHETPNLVLTDLLMPRMTGMDL
LAEIRKNPNTSHIPVILLSAAQDEESRVQALLYGADDFLSKPFKPRELLARVHLHM

>jgi|Umbra1|261491|UramaHK3 Rec1
TVLVCDDNTDMRSYIRHTLISNFNVIEASNGQEALDIAVRLAASEASEMSMQGDDAVPRR
RIDLVLADVMMPVMDGIELSKNLRANPFTRTLPIIMLTARAAAGDSMNGLFAGADDYLFK
PFDAQELIGRVTTHSN

Group XII Rec2 (n = 20)

>tr|J9VXZ1|CneofTCO2 Rec2|1670-1799
HVLIVEDNLINQTVLARQLKHCNLTCDVANDGLEALEKIRKVSSIENESDGQAFDCVLMD
LEMPVMDGLTAVGHIREEENAGKLKKNLVIALTGNARQGQIDLAKASGMDEVVIKPYRLD
DLLHKIEEVI

>tr|J9VXN3|CneofTCO4 Rec2|1545-1677
HVLIVEDNLINQTVLKRQLVKAGLSCNVASNGLEALNVIREVHRQHRRGGPNRKRLFDVV
LMDLEMPVMDGITAVREIRQSEAAGTLGRNMVIALTGNARQGQIDHALASGFDDVVIKPY
ILVDLLNKIKSMK

>jgi|Morel2|153930|MelonHK3 Rec2
KCVLCVEDNIINLRVVQYQLQKLGYDTLSACNGQIAVDIINSQVEMLGERTASLRLDGEE
SGVGNTDGDVSMDTADEGDQHRHHLIKGGLLVIPESNGNMSHIFDQTEPSSPSNLPGLST
FSAASTTTGSPLLSSSSAHFEGHAAPLASTLAAALGASTYSPTMQPTVSSGSRPPKIDLI
LMDCAMPVKSGFEAASEIRAIGQASSFAAQIPIIALTASAVPSTKEKCLAAGMNGYLSKP
TKLADLEATLDQWID

>jgi|Morel2|1836877|MelonHK13 Rec2
RILLAEDNAVNQKIAVGVLKKLGYENVDVAENGLEVIQKLDEGSIYDVILMDVSMPVMDG
IDATKTIVDRRLRGLLSNAAETSNCDTLAPEGSQSNGTHASQESSPERGYKDYQNLYVIA
LTASAMGSDKERCMEAGMDDFMTKPFALLEMKRVLNEYIH

>tr|F4RB40|MlariHHK3 Rec2|1953-2081
HVLVVEDNLINVKVIKRQLSLKGYSVSVAMDGRQGLDILYEDDQHPSELGRIGIVLMDIQ
MPVMNGLDAITELRASEKTGKVKQRYPVIAVTGNARKEQTEQFLASGFDDICVKPYKIED
VQNRMEALL

>tr|E3KZK9|PgramHK1 Rec2|2135-2263
HVLVVEDNTINVKVIKRQLTLKGYMVSVAMDGREALNRLYADASETSELPPIDIVLMDIQ
MPVMNGLEAITHLRAAEKTGEIKKRYPVIAVTGNARKEQTEQCLASGFDSICVKPYKIDD
VQSRMEALL

>jgi|Rambr1|29108|RbrevHK2 Rec2
RVLVVDDNSMTRSLTVRQLKHQHILNIDEATDGSFACFKYSSAQYDLVLMDLHMPRMDGF
LAARRMREIDSQRAKSMQISMASSIATASNTPITATFDSAEFNALKAAVSATSSMRRRRS
SSAQSVDGLNSAGCDAPNCIGTCLKVFEDMDASSDCSWTSQPDVATGSQAVTATATATAT
ATTAVTSSTGTATAFHRPVIVALTADRDVNQDAAADSGFDGTLVKPVPLNDFSKLLNKYF
G

>jgi|Gloin1|35016|RirreHK11 Rec2
KCILCVEDNPINLRVIQHQLAKLGYPTLSATNGQEAVNVIEAEIANSTPNDESPRISLIL
MDCAMPEMSGFDASKAIRLFQSPLSKIPIIALTASAVQGTRDRCLESGMNDYLTKPLKIG
QLKEMLEKWLG

>jgi|Synplu1|327098|SplumHK3 Rec2
QVILVVEDNPLNAKLMCHIIKNFGYRAEHASSGEEALSLTMEKNYALIFMDYQLPGMDGC
QSANKIRQQCVLNGRPRMPIIALTGMSGDRIRERCHEYGIDDYLTKPVVKHTLYEVLNQW
CC

>jgi|Sporo1|24426|SroseHHK3 Rec2
KVLVVEDNLVNQKILCRQLKNAGYETQVANNGVEALEAFEHDSNTDRSIKICLCDIQMPV

LDGLSAVRILREREKNGVINRRYPCIAVTGNARQAQQDECLQAGFDFVLSKPYTFTTVVE
RIEALKL

>jgi|Sporo1|28207|SroseHHK9 Rec2
KVLCCEDNQINRTVLKRQLVKEGVAEVLLACDGQEGIDMLYQREPGMIDCILMDIEMPVL
DGLAATRQIRLDEKEGRRKGHQRIVGLTGNARSAQKEAALEAGMDLVVTKPYKVPELIAK
IRNDVA

>tr|K1VJU5|TasahHHK1 Rec2|1124-1276
NVLVVEDNDINSTVLRRQILKAGLDCDVADNGLVALNHLLAVQDLPPLTPGSEASINLEP
PLSPVGSAGSAQRQPYDVVLMDLEMPVMDGLTAVRRIRQFEAEGKLPGHQLVIALTGNAR
EGQIEEALKSGMDDVVIKPYKLPQLLRTMEQAV

>tr|K1VTK5|TasahHHK3 Rec2|1759-1894
RVLIVEDNLINQTVLKRQLTKAGMTCDVAGNGEEALKLLRKRLRPASPSALSSQPDVEGA
YDVVLMDLEMPIMDGWTAIKILRASENPALQSQLVIALTGNAREGQIVEALKAGMDHVII
KPYKLDQLIEKMRGAV

>tr|K1W7Q9|TasahHHK4 Rec2|1577-1767
RVLIVEDNHINLRVLSRQLKHVGMDVDNAPNGLAALNMIRQAMAKPGIDFATFSPQFSAN
NIPLSSPLPATDLPPGGYVFGGPEPGPRRQHPLQSPLGGLPSSPPESPPMARSEPYDCIL
MDLEMPVMDGYTAASHVRADQASGKIGRVPIVALTGNARQAQIDGQMADFDDVVVKPYRL
DDLLRKIEKNA

>tr|K1VSA5|TasahHHK8 Rec2|1523-1647
RVLVVEDNIINRTVVLRQMKQAGVRAEAVTNGLEALERLRETQKPGGDRFDCVLMDLEMP
VMDGYTATKRLRAEEQAGRLERSRVVALTGNARQAMIQEQMRDFDDVAVKPYRLDALLGK
IAATT

>tr|K1VVT9|TasahHHK9 Rec2|826-915
ITELMGGHIEVASRYTEGSVFRFFVKTRAVNGLEAVERVRAAPFDCILMDLEMPVMDGYT
ATRTIREDEAQSVSASAIIALSRFSNPALI

>tr|K1VBK1|TasahHHK10 Rec2|1559-1680
SILVVEDNLVSQRVLQRQLTKLGNTVMVASNGQEALDTLVAQQGAFDMVLMDLEMPIMGG
LEAAQIVRLLEAEGKLPPQRIIALTGNAREEKVQQALDTGMNNVMIKPYRLPKLVGMIQA
TI

>jgi|Umbra1|261491|UramaHK3 Rec2
TILVAEDNLINQKLLKKQLLKLNYESDVVGNGFEAVNMLKTGKKYIAFLCDCNMPQCDGF
QATAIIRGSEESFRNIPILAISANAMAGDREKCLNGQMDDYISKPLTIPQLGSALERLLS

>jgi|Umbra1|119427|UramaHK12 Rec2
RTILLVEDNKINQNMMKRLLKMKGYEVLIADNGKLALSAFETKSFDLIIMDIQMPIMDGI
TATKEIRKLEKENAAAPIPIVGLSGNAREFHAVNALKSGLNRYMTKPVNKDELYAVIEQF
EQ

>jgi|Umbra1|255834|UramaHK13 Rec2
TILLVEDNKINQNMMKRLLKMKGYEVLIADNGKLALSAFETKSFDLIIMDIQMPIMDGIT
ATKEIRKLEKENAAAPIPIVGLSGNAREFHAVNALKSGLVRSFFHIFCWSSNDTDVDNIE
SL

Group XIII HHKs (n = 20)

>tr|A8N8P8|CcineHHK3|533-693
NVLVVDDDPLTRTLMTRILTRLGCQVTTAENGEMAIDIIMGVKGLSGLTPSTDSSKQLGP
ILEQPKKPPELLPSPGSGMSDGLAPDEHKFAVVFLDNQMPVMSGVRAVERLRQLGRTDFI
VGVTGNALLSDQQEYLEAGVDRVLTKPVLERSLREILNVAE

>tr|J9VDX9|CneofTCO5|1058-1180
SSLVVDDDKLTRMLMSRMLTRLGHYVTTAENGKIAAEMIKDMFENKEGAVKFDIVFLDNQ
MPLMSGVEVARAVREMNCPIYIVGCTGNALREDQDEYMAAGADTILTKPIHQKHLIEMIR
DAR

>tr|B0CTI1|LbicoHHK2|945-1095
SVLVVDDDHITRSLMKRLLTRLGCAVSVAENGEMALEMILGHQGLFGSTPSSDSSGSNCG
PILERRQNLRADVIFEEGKYAVVFLDNQMPVMSGLKAVEKLRQLGRKDFIVGVTGNALLS
DQQEYLEAGVDRVLTKPVYERSLRDILGEAE

>tr|F4RBK7|MlariHHK5|882-1003
KILLVDDNGMNVELGRRVLEKIGYEVQVAYNGLEAVQKACHSDCDLVLMDCQMPGVDGPE
ATRMIRDYETKAKEGVNRLPIIALTANVSESDQTTCMESGMDGFLPKPLQVTVLKATLHR
FL

>tr|K5VV46|PchryHHK3|925-1076
KVLVVDDDPLTRKLMSRMLTRFGCKVSTAENGEMALEMILGIGGARNIGTTPSSEEPSSA
GMSADGALGVRTSAGTDEPKYAVVFLDNQMPVLSGLETVAKLREMGRSDFVVGVTGNALL
SDQQEYLEAGVDHVLTKPVFEKSLRAMLVIAE

>tr|K5WPY2|PchryHHK5|963-1083
RILLVEDNMVNQHLGKRLLEKLGYAVETASNGQEAVEKASQSRYFCCFMDCQMPVLDGFM
ATQKIRELENNGTIHGRLPIIALTANVSTESEEKCRAAGMDHFLPKPFRMHDLQSALAHA
Q

>tr|K5X5D1|PchryHHK7|1558-1686
RVLVVEDNSILRGLLVKWLRSKGYEYREATDGLEGVQAFEGDGHFDVVLVDLSMPVLDGV
GATVKMRDFEASRKKEEQSDRRTQNARILALTGMSSLEDKRRAFDAGVDGYLVKPVAFKT
LDVMFHKLG

>tr|K5ULW9|PchryHHK8|900-1049
RVLVVDDDPLTRKLMTRMLTRLGCKVSTAENGEIALELILGAHSSNRPTPTSEETGSAGL
SVEALALAGAGANIDEYKYAVIFLDNQMPVLSGLNAVARLREIGRKDFVVGVTGNALLSD
QQEYLEAGVDRVLTKPVYEKSLKNVLAFAD

>tr|K5VVU4|PchryHHK9|975-1116
RVLVVDDDPLTRKLMSRMLTRLGCKVTTAENGEIALDLILNPAARPTPSSEDTGSSGLVS
NDGQLTTDERYAVVFLDNQMPVMSGLEAVAKLRRRGRKDFVVGVTGNALLTDQQEYLDAG
VDHVLTKPVLEKSLKAMLAIAD

>tr|A0A067NQY6|PostrHHK3|909-1051
NVLVVDDDPLTRTLMKRILTRLGCSVSTAENGEVALEMILAPAGQTPSSDGSHSNGPILE
QERPPQSQDTKYAVVFLDNSMPVMSGLELVTKLREMDRKDFVVGVTGNALLTDQQEYLEA
GVDRVLTKPVLERSLKDVLVIAD

>tr|A0A067NRD8|PostrHHK7|579-713
VYDFVEDGTNCFRDSASVLSGGEGGGCTVETAENGEAALAMILRSQYSGDAPENPEDETS

NGPYDVVFMDNQMPVMSGLTAVAKLRALSRHDFIVGVTGNALLTDQHEYLEAGVDKVLTK
PVLEKSLKEMLSLAL

>tr|B8P7I4|PplacHHK3|702-847
RVLVVDDDLLTRRLMSRMLTRIGCKVATAENGEIALEMILGSHATPSSEDTGSAGLSTEG
TTASAAADSSEEYRYAVVFLDNQMPILSGLDVVTKLREMGRSDFVVGVTGNALLTDQREY
LEAGADHVLTKPVLEKSLKSMLVIAD

>tr|B8PHK4|PplacHHK5|962-1082
TVLLVEDNPVNQRLGCRLLEKLGYAVVTANHGQEALDAISRSTFYCCLMDCQMPVLDGFA
TTRKVRELESEGTLQGHLPIVALTANVTTDCESLCRQAGMDHFLPKPLVLADLEETLKSY
G

>jgi|Rozal1_1|3104|RalloHK4
NLLVVEDNIINQKVLIKMINEILKNCIPFSIDVAENGLVALEKVDQKFYQVVFMDIQMPV
MDGVEASLKICEKYPDCSKRPIIAAVTANAMKTDANQNIQRYFDYYLNKPLTITKIKSIF
SKLGL

>jgi|Gloin1|327799|RirreHK5
SFLIVEDNKINEMILTTMLKQSGYYNYDIANNGLEGWIDYIIFGCFSFLFFTLNASKIFI
IAVENFTKKSYDIIFMDLQMPICDGIVATKEIRKIERGEESYLLQSHTSSPKSQSPSDTS
AEGTCKIGRRRRKKAIIVAMTGLASDASIDKIFKRTYGKLDERSIKGWGHTKVSVNE

>jgi|Synplu1|742021|SplumHK1
RVLVVDDNSINRSLLTRQLELLGVQTIESAANGEEACAKFQPGHYHLILMDVRMPRMDGY
TAARVMREKESSFIHDTASVIPVSSTNHPNYSDTTIGTNANHNNGDMLAINEKSTAISSP
NHHCRCVGSNYRHNRCNCHVLRSTPITTSSASSNVNSATASPSRAPQLRAAIVALTADRQ
LATGEGRLQITRCGMDDVLIKPINLPGLTALLHAWLPS

>jgi|Sporo1|29081|SroseHHK4
RVLVVDDDNLTRRLMGRMMSRLGCTVDMAENGKIALDMMLRPEPGKELEIAEAPPRGGDE
FDAEERGERLPAKMKKTAFGTFGIDPRSGVDAHKNFDIVFLDNQMPVMSGVQLVTKLRAL
GRDDLVVGVTANARMFSCLSSPRSRASNRKVADVASFRNCSLVLSDQEQYLEQGASFVLT
KPVSEDSLKKYLLIADR

>tr|K1VQS6|TasahHHK7|649-768
NTLVVDDDELTRDLMMRTVRRLGHQVILARNGMEALRVLKAAYEDGNPIDIVFLDNQMPA
MSGVETARAIRALQWDVYIVGCTGNALQEDQDEYKQAGADAIVTKPVSVAAIREKLDLVR

>tr|K1VIP4|TasahHHK12|556-674
SALVVDDDSLTRRLMSRMLSRLGHMVTTVDNGKKAVDLILENAADNPFDIVFLDNQMPIM
TGVEAVAALRQVGNDTYVVGCTGNALREDQDEYLSSGANAIIAKPVHQAAIIEHLAEAK

>tr|R4XCM5|TdefoHK5|801-927
SVLVVDDDLLTRRLMTRMISRLGHQVQSVENGLLALNLVRLHFRKPLADQPVRTPFDIVF
LDNQMPVLSGVEMVTRARMEGIDTMVVGVTGNAMREDQDEYLECGADFVLIKPVMEPSIS
KMLVEAK

Group XIV HHKs (n = 8)

>jgi|Catan1|54886|CanguHK7
HLLVVEDNMINQRILRNMLHKLGYTQITFAADGADALFHYNSLKDEGKYFDVILMDISLP
SLSGNDTCAWIRQKDRTQVIVMCTADTQMVGNMARWKACGYDDAIEKPVFVDVLSTILNR
WLE

>tr|Q6SLD7|CheteHHK10|865-995
KLLLVDDNPVNLKVLGMYARKCSKTAAKSVDGGQKAIDAFKAAFFDEDKTAQRFDLIFLD
LSMPEVSGFDVARQIREIEAKWESQSRVYICALTGLSSDKDRNAAYASGVDQYLVKPARL
DDLQWVVGRWQ

>tr|J9VVM9|CneofTCO7|1455-1624
KVLVVEDNVINRKILTTMLRRVSCSFAEAVDGVDAVDQFSVFQPDLVLLDITMPRKDGFA
AAAEMRRLETVRQIAEKDVPNPSSPIAAELIENALGSLDLNAHAPGSSSVSSSPGSSHSI
KLKKRARIIAVTAMSAEHQKRKGLYECGIDHWMTKPLSMSVLRSMVEKMK

>jgi|Rozal1_1|946|RalloHK5
RIAIVDDNAVCQRILEKQLSILGFSKISVFQSIEKILSQIQDKTVFDIIISDLLLLGSIN
GDVGSRLLRDLLPDCLIIIVTGLSRNEIGEHCADVVIEKPVSLEELRDALVRSHM

>jgi|Sporo1|33425|SroseHHK10
HILFADDNPVARNILIKLFTGKGVSFSAAEDGQQAVDLFEAAQGSVSMALLDVQMPNLDG
IGAAHAIREIEAERGWERSRIIALTGLSNESDMAEAMEGGVDKWLIKGGKSLRAILDEVV
ELQ

>tr|K1W7S7|TasahHHK11|1329-1464
SVLVVEDNVINRKILVKILRQALGKECELAEATNGKVALDMFRDLSRRRPIIVLLDINMP
VLDGWATASEMRAIEREREKLRAGLGRKRGPSKIFAVTALAGQNEKRRGLVECGFDGWLT
KPCDRDTLCRVVESAR

>tr|A0A0D1DRU0|UmaydHK4|597-722
SVLCCEDNPLNMRILTTMLRQAQIEFHEAVDGIEAVEQFKKYLPAVTLLDINMPRMMGFD
SCVLMRKHVNEQLSAEQQAARYFKIVAVTALSDGFHQQKGIQCGMDAWLTKPLQMRKLKL
DLAEWK

>tr|A0A0D1DQK1|UmaydHK5|1077-1192
RVLVVEDNPINMRLLTTLCKRLDIRYEEAHDGAEAVAKFISFRPSVVLLDISLPIQDGFE
ACAQMRAHGHSSFIVAVTALSSDEDKMRGIESCGMDAWMTKPVSPRQLKGDLEAWR

Group XV HHKs (n = 6)

>tr|A0A068S757|AcoryHHK14|793-924
KIMLAEDNVLNQKIAISILKRLGYHDVVVANNGREVLELMREIKFDVIFMDLYMPEMDGL
EATRAIISERELGTNESDNAPPLMNVSEVYIIALTASASRQDRQICIDAGMNDFISKPFT
MMEMKASLKNCV

>tr|S2K2Y8|McircHHK7|640-778
KILLAEDNVLNQKIAVSILKRLGFQDVIIAGNGREALELMRVHSFDVIFMDLYMPEMDGL
EATRYIISERKHNVPPPPILPENGQQQKPLLNANDVYIIALTASASKQDRQICIDAGMND
FISKPFTMMEMKSALKNCA

>tr|S2K3U9|McircHHK8|852-994
RILLAEDNILNQKIAISILKRLGYVDVAIANNGSEVLTLMKTSVFDVIFMDLYMPEMDGL
EVTREIIKERTRQKQADMAPNDTAQTNEFSASDLLNTVDVYIIALTASASREDRQICIDA
GMNDFISKPFTMTEMKSALKTCA

>jgi|Morel2|1241069|MelonHK10
RILLAEDNAVNQKIAVGVLKKLGYENVDVAENGLEVIQKLDEGSIYDVILMDVSMPVMDG
IDATKTIVDRRLRGLLSNAAETSNCDTLAPEGSQSNGTHASQESSPERGYKDYQNLYVIA
LTASAMGSDKERCMEAGMDDFMTKPFALLEMKRVLNEYI

>tr|I1BLT2|RdeleHHK12|406-455
KILLAEDNILNQKIALSILKRLGYQDVTTAGNGREALELMIKHDYDVIFH

>jgi|Umbra1|240602|UramaHK9
KILLAEDNVLNQKIAISILKRLGYTGVEIANNGREVLDAMRRSRFDLIFMDLYMPEMDGL
EVTKSIISARHSPNTELNNVHDVYIIALTASASMQDRQICIEAGMNDFISKPFTMLEMKQ
SLKKCM

Group XVI HHKs (n =6)

>tr|A0A068RE24|AcoryHHK16|1852-1975
RSLLVDDNPVNQKVVARMLSRLGIKPEVAQNGKEACDKIEASRKQGEPIDLVFMDIWMPE
MNGLEAAETIRKSLADSSLHPYIIAMTACVMPGDREKCIAAGMNGYVSKPVRKDELEASI
HTYT

>tr|A0A068S7H3|AcoryHHK17|1713-1837
KTLLVDDNPINRKVVSRMLSKLNVQPLMAQNGREAYDLVINSAAQGQPIDLIFMDVWMPE
LNGLEATRMIRETPDTLIGKQPYIIAMTACVMPGDREQCFDAGMNAYISKPIQKDELEAT
IHIFT

>tr|S2IXX2|McircHHK9|1095-1218
RSLLVDDNPINQKVLSRMLTRMGMHPQIAGNGREACDIVASAGDSGEPIELIFMDIWMPE
MNGLEAATKIRQELASSAVNPYIIAMTACVMPGDREKCIDAGMNGYVSKPVRKEELEAAL
HTYT

>jgi|Morel2|1153927|MelonHK11
KVLVVDDNPVNLKVVCKMLGRLGVEPETANNGQEAVELIEKKIALLSLQLEGASAPTQAD
GSEAAPLPLALPLPIEHALSDANMQRQRPHLVPKAGTQQRVQHSDSSSTTSSTHGIDSGL
GLLTDSDDQSIVPHL

>jgi|Morel2|1827482|MelonHK12
CLLVDDNPVNQKVISKMLSRIGIVPELAANGLEAVEKCRARAEAVAAAATAEGGGDGSSL
TSNTTAGGGGAKKTKQYDIVFMDIWMPVISGHEATKEIRATVPGVTATSPLIVAMTACVM
PGDQQKCIDSGMNRYLSKPIRKEELSKTLEEWLD

>tr|A0A1Y1V617|PfinnHHK1|1142-1259
SVLVADDNIINQNVLKRMLDSYLKITADTANNGLEVLEALKKKNYDLIFMDFHMPLMDGL
KATGKIRQLYPLSPIKIVAVTAAALPGDKEQCLESGMDDYITKPIKKDDLCKMIQRLW

Group XVII HHKs (n =3)

>tr|G1XE95|AoligHK11|759-881
VILLVEDNPINQQLGVKMLKKLGRAVDVAENGKVALEKIMENRRQYSLVLMDSQMPVLSG
PSTCRRIRAMEAAGELPGRLRIISLTANVDSLSQKECMEAGSDHFLPKPLTLKLLREELE
RPQ

>tr|E3KAG1|PgramHK4|846-968
KILLVDDNPINVQLGARLLGLLGYQVDSAANGYEAVTKACGSAYDLILMDCQMPELDGLS
ATRKIREYEAHFRGKRSRPVPIIALTANVSESDQADCRLSGMDGFLPKPLQINVLKSTLH
TFL

>jgi|Usnflo1|824868|UflorHK3
SVILLVEDNPINQKLGKKMLAVLKYQVLVAHDGEEAIAQIMKHDATIDAILMDQSMPNMD
GITATKEIRAMEAARTLSRRRPIIAVTAVVSTQAQALFRVAGADDFLTKPLSLSKLEQTL
AAHLP

Group XVIII HHKs (n = 5)

>jgi|Catan1|49608|CanguHK1
SVLVVDDNPINLKVAVKVISSAGHRGHISTATNGREALELLASKQYDVVFMDVSMPTMDG
LEATRLLRERERTGAGSAAAVHWVCAMTASALPEERSACLKAGMNDFISKPIKKDTVIRT
LQSAMC

>jgi|Catan1|40330|CanguHK2
KVLIVDDNNINQMVAVRTLRSIGVTNVNTANNGAEAVAYVEAHPDVDVVFMDVSMPVMDG
LEATRAILANAAAKVVMFGVKPVLLGRRTSNSTAVSRSPSHSNLGVPGSIPPVFDGSDGI
HGKGLPFICAMTASALPEERTTCLNTGMHDFVPKPIRRNDLLEVLGRYC

>jgi|Ganpr1|220489|GprolHK2
NILVVEDNQINQMVTGRILQKLGQKFEVADDGIKALALFQEGRSFDIVLMDISMPRMDGY
QCAREIRKTLGGNAPERRSAASPTKPWIIALTANALYDDRVRALEAGMNDFVPKPAKQSD
IVDALVRYLE

>jgi|Rozal1_1|3629|RalloHK2
HILVVDDNAINRKVIAKLLESILCPQIDFAEDGAVAVKLALSKSYDIVFMDVSMPVMDGL
TATRLIKKEKKVFIVAMTANALYQERLLCMESGMDDYLAKPVSRDDVISLLERFLK

>jgi|Gloin1|346986|RirreHK6
KVLVVEDNVINQMVTSRILEKLNQKCEIAGSGKTAISKCEEKDYDVIFMDIMMADMDGFQ
TTEQIRSTSQDLRRPWIIALTANALWYDRFRCIESGMNDFVSKPAKKEDIREALMRYLT

Group XIX HHKs (n = 7)

>jgi|Catan1|64655|CanguHK3
PLVVLLVEDNALIQALTKRFLERDGFHVVVADHGQAALDLLADPDLSSRVHLVVMDLMMP
VLDGIDATRIIRQTRSPAELPIVALTANAMAEERERCLQSGFNAFLTKPVTRQKLIETVR
SLWSA

>jgi|Catan1|74130|CanguHK4
RILVVEDNTLVLRVTCKLVKTLGYEADTAMDGLECVKRMNDESKPKVDLILMDLVMPNMS
GMDAMKTLRDAGYSKDALPIVAVTANALPEEREACMEAGFNEFVTKPLKKDGLVDICKRF
IF

>tr|A0A1Y2H816|CanguHK8|997-1117
VVLLVEDNALIQALTKRFLERDGFHVVVADHGQAALDLLADPDLSSRVHLVVMDLMMPVL
DGIDATRIIRQTRSPAELPIVALTANAMAEERERCLQSGFNAFLTKPVTRQKLIETVRSL

W>jgi|Ganpr1|285950|GprolHK3
TIILVVEDNGLVAKLTQRMLKRGGFKSEIATDGVVCVDMVRNIGIEGISLVLMDLQMPRL
DGFGAARHLRSDLHFAGPIVALTAFTTNNDVEQCLEGSLMQEVLGKPVTEAKLLETVRKF
IG

>jgi|Ganpr1|468424|GprolHK4
HVLVVEDNIVVAKVTSKMLARNGYQVDLADDGLKCVQRVKVRALNGYDVILMDLHMPNLD
GVGAAQQLRELGFDRAIVALTGVSKSYTAQEDQRKCLDSGLMQAVLGKPVKEQDLINCVT
RYSG

>jgi|Gloin1|22775|RirreHK7
RILLVEDNLVCQRVTYKMLNRNNYSVDIANHGKEAVDMVEAVQQQQQQYACILMDIITP
VMNGYEATQILRDRGVKIPILALTANSFESDVKKAKEVGMDDFLTKPIKEVELITAMKTQ
IE

>jgi|Umbra1|280328|UramaHK10
PVCLVAEDNELCQKIATKMLGKEYVVEIAANGQIAVDTVMAAPDRFNIIIMDIIMPEMDG
IQATIELRKNGIKIPIIAVTANAAEADKKEAMRHGFNGYITKPYKRNLLTAMQRALSA

Unclassified HHK (n = 1)

>jgi|Gloin1|77355|RirreHK12
KVLMVEDNETNQRIALHYLEKMGQKVTLAANGIEALEKMKNETFDILFVDLQMPYMVNNI
L
