# Supplementary material for: Predicted Functional and Structural Diversity of Receiver Domains in Fungal Two-Component Regulatory Systems
Source: mSphere. 2021 Oct 6;6(5):e00722-21. doi: 10.1128/mSphere.00722-21 (PMC8510515; doi:10.1128/mSphere.00722-21)
Supplement: TABLE S1 [file msphere.00722-21-st001.pdf]

Table S1. Fungal receiver domain inventory

Fungal Receiver Domain Type	Number of									
	Total Database Entries	Non-Fungal Entries	Reported Sequence Not Found	Expected Response Regulator Not Found	Entry Lacks Receiver Domain	Net[a] Fungal Entries Analyzed	Normal Receiver Domain	Atypical Receiver Domain	Receiver Domain with Deletions	Degenerate Receiver Domain
Rim15 Ascomycota	27	0	0	1	2	24	0	24	0	0
Rim15 non-Ascomycota	31	1	0	7	0	23	18	4	0.5[b]	0.5
Skn7	61	0	0	6	0	55	53	1	1	0
Srr1	20	0	1	0	0	19	19	0	0	0
Ssk1	56	1	0	7	0	48	46	1	1	0
Unclassified RR	48	4	0	0	0	44	32	4	7	1
HHK I	50	0	0	0	0	50	48	0	2	0
HHK II	11	0	0	0	0	11	8	1	1	1
HHK III	57	0	0	0	0	57	55	1	1	0
HHK IV	13	1	0	0	0	12	11	0	0	1
HHK V	42	1	0	0	0	41	37	2	1	1
HHK VI	23	0	0	0	1	22	22	0	0	0
HHK VII	13	0	0	0	0	13	13	0	0	0
HHK VIII	36	0	0	0	1	35	34	0	1	0
HHK IX	21	0	0	0	0	21	21	0	0	0

HHK X	64	7	0	0	0	57	53	2	2	0
HHK XI	47	0	0	0	0	47	46	1	0	0
HHK XII Rec1	18	1	2	0	0	15	14	1	0	0
HHK XII Rec2	23	1	2	0	0	20	18	1.5	0.5	0
HHK XIII	21	0	0	0	1	20	19	0	0	1
HHK XIV	8	0	0	0	0	8	8	0	0	0
HHK XV	8	1	0	0	1	6	5	0	1	0
HHK XVI	7	0	0	0	1	6	5	0	0	1
HHK XVII	3	0	0	0	0	3	3	0	0	0
HHK XVIII	6	1	0	0	0	5	5	0	0	0
HHK XIX	7	0	0	0	0	7	7	0	0	
Unclassified HHK	1	0	0	0	0	1	0	0	1	0
Totals	722	19	5	21	7	670	600	43.5	20	6.6

[a]Net Fungal Entries Analyzed is equal to Total Entries minus Non-fungal Entries, Reported Sequences Not Found, and Expected Response Regulators Not Found. Net Fungal Entries Analyzed is also equal to the sum of Normal, Atypical, Deleted, and Degenerate Receiver Domains.

[b]Receiver domains with two abnormalities (atypical, deletion, degenerate) are listed as 0.5 in each category.
