# Supplementary material for: Predicted Functional and Structural Diversity of Receiver Domains in Fungal Two-Component Regulatory Systems
Source: mSphere. 2021 Oct 6;6(5):e00722-21. doi: 10.1128/mSphere.00722-21 (PMC8510515; doi:10.1128/mSphere.00722-21)
Supplement: TABLE S2 [file msphere.00722-21-st002.pdf]

Table S2. Amino acid frequencies at key receiver domain positions in fungal response regulators

		Percent							
		Bacterial Receivers		Fungal Receivers					
Position	Amino Acid(s)	All (n = 33,252)	HHKs (n = 8,113)	Skn7 (n = 55)	Rim15 Asc (n = 24)	Rim15 non-Asc (n = 23)	Ssr1 (n = 19)	Unclassified (n = 44)	Ssk1 (n = 48)
DD	Asp Asp	60	53	36	0	43	100	39	8
	Glu Asp	39	46	64	92	48	0	48	88
	Not Asp/Glu	0*[a]	0*	0	8	9	0	14	0
D	Asp	100*	100*	98	0	91	100	93	98
	Glu	0*	0*	0	100	0	0	0	0
	Other	0*	0*	2	0	9	0	7	2
T	Thr	69	69	96	100	65	100	77	100
	Ser	31	31	0	0	22	0	18	0
	Other	0*	0*	4	0	13	0	7	0
K	Lys	100*	100*	98	96	96	100	95	100
T+1	Ala	53	58	4	29	70	0	48	98
	Gly	22	30	5	58	4	0	5	2
	Ser	10	8	87	4	22	11	9	0
	Thr	7	0.8	0	0	0	89	27	0
D+2	Met	20	16	13	0	9	0	7	0
	Arg	13	9	0	21	35	0	0	0
	Asn	12	5	0	0	0	0	0	2
	Gln	10	23	0	4	9	0	16	96
	Glu	6	6	0	0	4	95	7	0
	Lys	5	3	0	75	9	0	0	0
	Val	5	7	38	0	4	0	16	0
	Ser	4	4	0	0	0	0	16	0
	His	3	5	0	0	22	0	5	0
	Thr	3	4	0	0	4	0	2	0

	Leu	3	3	2	0	0	0	2	0
	Ile	2	3	38	0	0	0	0	0
	Asp	2	2	0	0	0	5	14	0
	Tyr	1	0.6	2	0	0	0	0	0
	Trp	0.8	0.1	2	0	0	0	5	0
	Phe	0.7	0.4	5	0	0	0	0	2
T+2	Tyr	15	18	0	96	78	0	2	0
	Arg	14	10	0	0	0	68	0	0
	His	11	9	0	0	4	0	7	0
	Lys	11	8	0	0	0	26	2	0
	Leu	9	8	0	0	0	5	2	0
	Phe	8	4	0	0	13	0	2	0
	Ser	7	7	2	0	0	0	18	100
	Asn	5	13	93	4	0	0	41	0
	Asp	3	5	2	0	0	0	14	0
	Gln	3	3	0	0	0	0	0	0
	Met	2	1	0	0	0	0	0	0
	Val	2	2	0	0	0	0	0	0
	Ile	1	1	0	0	0	0	2	0
	Cys	0.4	0.3	0	0	0	0	5	0
K+1	Pro	82	89	98	96	88	100	93	100
K+2	Phe	39	27	95	0	0	5	20	2
	Val	14	22	0	4	57	11	23	83
	Ile	13	19	0	33	13	5	30	14
	Leu	6	9	4	4	26	74	14	2
	Tyr	5	8	0	0	0	0	2	0
	Pro	0.8	0.6	0	50	0	0	0	0
DD+1	Glu	28	19	2	0	0	0	0	0
	Asp	27	19	96	0	0	0	20	0
	Asn	13	34	0	0	100	100	68	98
	His	8	4	0	92	0	0	0	0

D+4	Pro	88	92	96	92	96	100	89	98
D+8	Gly	91	92	98	92	91	100	98	100
K-3	Tyr	62	45	0	0	9	0	27	8
	Phe	23	23	0	0	4	0	16	90
	Val	4	7	62	4	17	0	11	0
	His	3	10	0	0	0	0	2	0
	Leu	2	4	4	92	13	0	0	2
	Ile	2	4	29	4	30	0	9	0
	Trp	0.9	0.7	0	0	0	0	7	0
	Ala	0.5	0.8	0	0	0	0	9	0
	Thr	0.4	0.7	0	0	9	100[b]	0	0

[a]*Bacterial datasets were curated to contain only receiver domains with the five conserved active site residues. Atypical receiver domains were not excluded from analysis of fungal receiver domains.

[b]Srr1 response regulators contain 74% Tyr/Phe/His (typical bacterial K-3 residues) at position K-4, perhaps suggesting a slightly different structure.
