# Supplementary material for: Predicted Functional and Structural Diversity of Receiver Domains in Fungal Two-Component Regulatory Systems
Source: mSphere. 2021 Oct 6;6(5):e00722-21. doi: 10.1128/mSphere.00722-21 (PMC8510515; doi:10.1128/mSphere.00722-21)
Supplement: TABLE S6 [file msphere.00722-21-st006.pdf]

Table S6A. Amino acid frequencies at key receiver domain positions in fungal hybrid histidine kinase groups I to VII

		Percent								
		Bacterial Receivers		Fungal Receivers						
Position	Amino Acid(s)	All (n = 33,252)	HHKs (n = 8,113)	I (n = 50)	II (n = 11)	III (n = 57)	IV (n = 12)	V (n = 41)	VI (n = 22)	VII (n = 13)
DD	Asp Asp	60	53	58	55	0	17	0	41	8
	Glu Asp	39	46	42	45	98	83	98	59	92
	Not Asp/Glu	0*[a]	0*	0	0	2	0	2	0	0
D	Asp	100*	100*	96	91	100	100	95	100	100
	Glu	0*	0*	0	0	0	0	0	0	0
	Other[b]	0*	0*	4	9	0	0	5	0	0
T	Thr	69	69	86	82	100	83	15	77	15
	Ser	31	31	12	9	0	17	80	23	85
	Other	0*	0*	0	9	0	0	5	0	0
K	Lys	100*	100*	98	91	100	92	90	100	100
T+1	Ala	53	58	10	36	100	83	93	100	100
	Gly	22	30	86	55	0	17	2	0	0
	Ser	10	8	2	0	0	0	0	0	0
	Thr	7	0.8	0	0	0	0	0	0	0
D+2	Met	20	16	0	0	0	8	10	0	0
	Arg	13	9	0	0	0	17	0	0	0
	Asn	12	5	6	9	0	25	0	0	0
	Gln	10	23	16	55	75	0	34	100	0
	Glu	6	6	0	0	0	0	0	0	0
	Lys	5	3	0	0	0	0	0	0	0
	Val	5	7	0	0	0	0	0	0	0

	Ser	4	4	68	9	25	33	5	0	0
	His	3	5	0	0	0	0	34	0	0
	Thr	3	4	6	9	0	0	0	0	0
	Leu	3	3	0	0	0	0	2	0	0
	Ile	2	3	0	0	0	17	0	0	0
	Asp	2	2	0	9	0	0	0	0	0
	Tyr	1	0.6	0	0	0	0	0	0	0
	Trp	0.8	0.1	0	0	0	0	0	0	100
	Phe	0.7	0.4	0	0	0	0	12	0	0
T+2	Tyr	16	18	0	0	0	33	0	9	0
	Arg	14	10	4	0	0	0	0	0	0
	His	11	9	0	0	100	0	0	0	0
	Lys	11	8	0	0	0	0	0	0	0
	Leu	9	8	60	0	0	0	0	5	0
	Phe	8	4	4	0	0	0	0	86	0
	Ser	7	7	2	9	0	0	7	0	0
	Asn	5	13	2	9	0	42	83	0	0
	Asp	3	5	0	18	0	25	5	0	100
	Gln	3	3	0	45	0	0	0	0	0
	Met	2	1	10	0	0	0	0	0	0
	Val	2	2	10	0	0	0	0	0	0
	Ile	1	1	2	0	0	0	0	0	0
	Cys	0.4	0.3	0	9	0	0	0	0	0
K+1	Pro	82	89	96	91	98	92	95	100	100
K+2	Phe	39	27	2	0	0	0	7	0	8
	Val	14	22	72	55	0	67	56	9	0
	Ile	13	19	12	9	2	8	22	91	0
	Leu	6	9	6	18	96	8	5	0	0
	Tyr	5	8	0	9	0	0	0	0	92
	Pro	0.8	0.6	0	0	0	0	0	0	0
DD+1	Glu	28	19	0	0	0	0	0	0	0
	Asp	27	19	0	0	0	0	24	0	0

	Asn	13	34	98	100	100	100	71	100	100
	His	8	4	0	0	0	0	0	0	0
D+4	Pro	88	92	96	91	100	100	98	100	100
D+8	Gly	91	92	96	91	100	100	95	100	100
K-3	Tyr	62	45	30	36	86	83	66	0	54
	Phe	23	23	62	27	0	8	12	100	46
	Val	4	7	2	0	4	0	0	0	0
	His	3	10	0	0	11	0	15	0	0
	Leu	2	4	2	9	0	0	0	0	0
	Ile	2	4	0	0	0	0	0	0	0
	Trp	0.9	0.7	2	0	0	0	0	0	0
	Ala	0.5	0.8	0	9	0	0	0	0	0
	Thr	0.4	0.7	0	0	0	0	0	0	0

[a]*Bacterial datasets were curated to contain only receiver domains with the five conserved active site residues. Atypical receiver domains were not excluded from analysis of fungal receiver domains.

[b]Other could mean a different amino acid than those listed, the absence of an amino acid at this position in receiver domains with deletions, or the inability to identify the amino acid in receiver domains with degenerate sequence.

Table S6B. Amino acid frequencies at key receiver domain positions in fungal hybrid histidine kinase groups VIII to XIII

		Percent								
		Bacterial Receivers		Fungal Receivers						
Position	Amino Acid(s)	All (n = 33,252)	HHKs (n = 8,113)	VIII (n = 35)	IX (n = 21)	X (n = 57)	XI (n = 47)	XII Rec1 (n = 15)	XII Rec2 (n = 20)	XIII (n = 20)
DD	Asp Asp	60	53	6	5	4	0	73	5	70
	Glu Asp	39	46	94	95	93	98	13	90	30
	Not Asp/Glu	0*[a]	0*	0	0	4	2	7	5	0
D	Asp	100*	100*	100	100	100	100	100	100	100
	Glu	0*	0*	0	0	0	0	0	0	0
	Other[b]	0*	0*	0	0	0	0	0	0	0
T	Thr	69	69	11	90	82	83	27	80	100
	Ser	31	31	86	10	14	17	67	20	0
	Other	0*	0*	3	0	4	0	7	0	0
K	Lys	100*	100*	97	100	96	100	100	90	95
T+1	Ala	53	58	94	100	95	96	73	25	30
	Gly	22	30	3	0	0	2	7	70	70
	Ser	10	8	0	0	0	0	7	0	0
	Thr	7	0.8	0	0	2	0	0	0	0
D+2	Met	20	16	0	0	2	0	53	0	0
	Arg	13	9	0	0	2	0	0	0	5
	Asn	12	5	3	0	4	0	7	5	0
	Gln	10	23	94	81	40	0	0	30	85
	Glu	6	6	0	0	0	100	0	45	0
	Lys	5	3	3	0	2	0	0	0	0
	Val	5	7	0	0	0	0	0	0	0

	Ser	4	4	0	0	0	0	0	5	10
	His	3	5	0	10	47	0	0	5	0
	Thr	3	4	0	0	0	0	0	0	0
	Leu	3	3	0	0	0	0	33	0	0
	Ile	2	3	0	10	0	0	0	0	0
	Asp	2	2	0	0	0	0	0	0	0
	Tyr	1	0.6	0	0	0	0	0	0	0
	Trp	0.8	0.1	0	0	0	0	0	0	0
	Phe	0.7	0.4	0	0	2	0	0	0	0
T+2	Tyr	16	18	0	0	0	17	0	0	0
	Arg	14	10	0	0	0	0	27	0	0
	His	11	9	0	0	0	0	13	0	0
	Lys	11	8	0	0	0	0	13	0	0
	Leu	9	8	0	0	0	0	0	0	5
	Phe	8	4	0	0	0	0	0	5	0
	Ser	7	7	80	100	35	0	0	15	0
	Asn	5	13	0	0	11	81	0	70	85
	Asp	3	5	0	0	47	0	0	5	5
	Gln	3	3	9	0	0	0	13	0	0
	Met	2	1	3	0	2	0	0	5	5
	Val	2	2	0	0	0	0	13	0	0
	Ile	1	1	0	0	0	0	13	0	0
	Cys	0.4	0.3	0	0	0	0	0	0	0
K+1	Pro	82	89	97	100	96	100	100	90	95
K+2	Phe	39	27	0	0	4	85	100	10	5
	Val	14	22	14	90	37	15	0	15	65
	Ile	13	19	71	10	12	0	0	0	10
	Leu	6	9	11	0	39	0	0	10	15
	Tyr	5	8	0	0	0	0	0	55	0
	Pro	0.8	0.6	0	0	0	0	0	0	0
DD+1	Glu	28	19	3	0	0	0	0	0	0
	Asp	27	19	66	0	5	0	0	0	55

	Asn	13	34	31	100	93	100	67	95	40
	His	8	4	0	0	0	0	0	0	0
D+4	Pro	88	92	100	95	100	100	93	100	100
D+8	Gly	91	92	100	100	95	100	100	100	100
K-3	Tyr	62	45	9	100	74	0	80	25	10
	Phe	23	23	3	0	16	0	13	5	15
	Val	4	7	0	0	0	83	0	45	55
	His	3	10	0	0	4	0	0	0	0
	Leu	2	4	0	0	0	2	0	0	0
	Ile	2	4	0	0	2	2	0	10	15
	Trp	0.9	0.7	86	0	0	0	0	0	0
	Ala	0.5	0.8	0	0	0	11	0	0	0
	Thr	0.4	0.7	0	0	0	0	0	5	0

[a]*Bacterial datasets were curated to contain only receiver domains with the five conserved active site residues. Atypical receiver domains were not excluded from analysis of fungal receiver domains.

[b]Other could mean a different amino acid than those listed, the absence of an amino acid at this position in receiver domains with deletions, or the inability to identify the amino acid in receiver domains with degenerate sequence.

Table S6C. Amino acid frequencies at key receiver domain positions in fungal hybrid histidine kinase groups XIV to XIX

		Percent							
		Bacterial Receivers		Fungal Receivers					
Position	Amino Acid(s)	All (n = 33,252)	HHKs (n = 8,113)	XIV (n =8)	XV (n = 6)	XVI (n = 6)	XVII (n = 3)	XVIII (n = 5)	XIX (n = 7)
DD	Asp Asp	60	53	38	0	100	33	60	0
	Glu Asp	39	46	63	100	0	67	40	100
	Not Asp/Glu	0*[a]	0*	0	0	0	0	0	0
D	Asp	100*	100*	100	83	83	100	100	100
	Glu	0*	0*	0	0	0	0	0	0
	Other[b]	0*	0*	0	17	17	0	0	0
T	Thr	69	69	100	83	83	100	100	100
	Ser	31	31	0	0	0	0	0	0
	Other	0*	0*	0	17	17	0	0	0
K	Lys	100*	100*	100	83	83	100	100	100
T+1	Ala	53	58	63	83	83	100	100	86
	Gly	22	30	38	0	0	0	0	14
	Ser	10	8	0	0	0	0	0	0
	Thr	7	0.8	0	0	0	0	0	0
D+2	Met	20	16	0	0	0	0	20	29
	Arg	13	9	0	0	0	0	0	0
	Asn	12	5	25	0	0	67	0	0
	Gln	10	23	13	0	0	0	0	14
	Glu	6	6	0	0	0	0	0	0
	Lys	5	3	0	0	0	0	0	0
	Val	5	7	0	0	0	0	0	14

	Ser	4	4	38	17	0	33	80	0
	His	3	5	0	0	17	0	0	14
	Thr	3	4	13	0	0	0	0	0
	Leu	3	3	13	0	0	0	0	0
	Ile	2	3	0	0	0	0	0	29
	Asp	2	2	0	0	0	0	0	0
	Tyr	1	0.6	0	67	0	0	0	0
	Trp	0.8	0.1	0	0	67	0	0	0
	Phe	0.7	0.4	0	0	0	0	0	0
T+2	Tyr	16	18	0	0	0	0	0	0
	Arg	14	10	0	0	0	0	0	0
	His	11	9	0	0	0	0	0	0
	Lys	11	8	0	0	0	0	0	0
	Leu	9	8	75	0	0	0	0	0
	Phe	8	4	0	0	0	0	0	14
	Ser	7	7	0	83	0	0	40	0
	Asn	5	13	0	0	0	67	60	71
	Asp	3	5	13	0	0	0	0	0
	Gln	3	3	0	0	0	0	0	0
	Met	2	1	13	0	0	0	0	0
	Val	2	2	0	0	0	33	0	14
	Ile	1	1	0	0	0	0	0	0
	Cys	0.4	0.3	0	0	67	0	0	0
K+1	Pro	82	89	88	83	83	100	100	100
K+2	Phe	39	27	0	83	0	0	0	0
	Val	14	22	38	0	33	0	40	57
	Ile	13	19	0	0	50	0	40	14
	Leu	6	9	25	0	0	100	0	14
	Tyr	5	8	0	0	0	0	0	14
	Pro	0.8	0.6	0	0	0	0	0	0
DD+1	Glu	28	19	0	0	0	0	0	0
	Asp	27	19	0	0	0	0	0	0

	Asn	13	34	100	100	83	100	100	100
	His	8	4	0	0	0	0	0	0
D+4	Pro	88	92	88	83	83	100	80	100
D+8	Gly	91	92	100	83	83	100	100	100
K-3	Tyr	62	45	13	0	83	0	20	14
	Phe	23	23	0	83	0	100	80	57
	Val	4	7	13	0	0	0	0	29
	His	3	10	0	0	0	0	0	0
	Leu	2	4	0	0	0	0	0	0
	Ile	2	4	0	0	0	0	0	0
	Trp	0.9	0.7	63	0	0	0	0	0
	Ala	0.5	0.8	13	0	0	0	0	0
	Thr	0.4	0.7	0	0	0	0	0	0

[a]*Bacterial datasets were curated to contain only receiver domains with the five conserved active site residues. Atypical receiver domains were not excluded from analysis of fungal receiver domains.

[b]Other could mean a different amino acid than those listed, the absence of an amino acid at this position in receiver domains with deletions, or the inability to identify the amino acid in receiver domains with degenerate sequence.
