# Supplementary material for: Predicted Functional and Structural Diversity of Receiver Domains in Fungal Two-Component Regulatory Systems
Source: mSphere. 2021 Oct 6;6(5):e00722-21. doi: 10.1128/mSphere.00722-21 (PMC8510515; doi:10.1128/mSphere.00722-21)
Supplement: TABLE S3 [file msphere.00722-21-st003.pdf]

Table S3. Most common D+2/T+2 pairs by fungal receiver domain type

Receiver Domain Type	Number in Database	D+2/T+2 Pair	Number	Percent
Skn7	55	IN[a]	21	38
		VN	19	35
		MN	7	13
		FN	3	5
		Sum	50	91
Rim15 Ascomycota	24	KY	17	71
		RY	5	21
		Sum	22	92
Rim15 non-Ascomycota	23	RY	8	35
		HY	4	17
		QY	2	9
		MF	2	9
		Sum	16	70
Srr1	19	ER	13	68
		EK	4	21
		Sum	17	89
Unclassified	44	VN	6	14
		DN	4	9
		QS	3	7
		SD	3	7
		DS	2	5
		QN	2	5
		SN	2	5
		SS	2	5
		Sum	24	55
Ssk1	48	QS	46	96
		Sum	46	96
Group I	50	SL	22	44
		QL	5	10
		SM	4	8
		SV	4	8
		Sum	35	70
Group II	11	QQ	4	36
		Sum	4	36

Group III	57	QH	43	75
		SH	14	25
		Sum	57	100
Group IV	12	SN	2	17
		SY	2	17
		NN	2	17
		RD	2	17
		Sum	8	67
Group V	41	HN	14	34
		QN	13	32
		FN	5	12
		Sum	32	78
Group VI	22	QF	19	86
		QY	2	9
		Sum	21	95
Group VII	13	WD	13	100
		Sum	13	100
Group VIII	35	QS	28	80
		QT	2	6
		QQ	2	6
		Sum	32	91
Group IX	21	QS	17	81
		HS	2	9
		IS	2	9
		Sum	21	100
Group X	57	HD	26	46
		QS	19	33
		QN	4	7
		Sum	49	86
Group XI	47	EN	38	81
		EY	8	17
		Sum	46	98
Group XII	15	MK	2	13
Rec1		MR	2	13
		MH	2	13
		LI	2	13
		LV	2	13
		Sum	10	67

Group XII Rec2	20	EN	8	40
		QN	4	20
		AS	<u>2</u>	<u>10</u>
		Sum	14	70
Group XIII	20	QN	<u>16</u>	<u>80</u>
		Sum	16	80
Group XIV	8	SL	2	25
		NL	<u>2</u>	<u>25</u>
		Sum	4	50
Group XV	6	YS	<u>4</u>	<u>67</u>
		Sum	4	67
Group XVI	6	WC	<u>4</u>	<u>67</u>
		Sum	4	67
Group XVII	3	QN	<u>2</u>	<u>67</u>
		Sum	2	67
Group XVIII	5	SN	2	40
		SS	<u>2</u>	<u>40</u>
		Sum	4	80
Group XIX	7	IN	2	29
		MN	<u>2</u>	<u>29</u>
		Sum	4	57

[a]Single letter amino acid code.
