# Supplementary material for: Predicted Functional and Structural Diversity of Receiver Domains in Fungal Two-Component Regulatory Systems
Source: mSphere. 2021 Oct 6;6(5):e00722-21. doi: 10.1128/mSphere.00722-21 (PMC8510515; doi:10.1128/mSphere.00722-21)
Supplement: TABLE S4 [file msphere.00722-21-st004.pdf]

**Table S4. Unclassified fungal response regulators with identified domains[a]**

| Response Regulator | UniProtKB ID or JGI # | SMART or Pfam Domain Name (description) | SMART or Pfam Domain Number |
|---|---|---|---|
| CanguRR4 | A0A1Y2HQ52 | PAS[b] | SM00091 |
| CanguRR10 | A0A1Y2HN41 | HDc (metal dependent phosphohydrolase) | SM000471 |
| MelonRR1 | Morel2\|156655 | Cation_ATPase_N (Cation transporter/ATPase, N-terminus) | PF00690 |
| | | E1-E2_ATPase (E1-E2 ATPase) | PF00122 |
| | | Hydrolase (haloacid dehalogenase-like hydrolase) | PF00702 |
| PostrRR1 | A0A067PB73 | PK_Tyr_Ser-Thr (protein kinase) | PF07714 |
| PplacRR1 | B8PJM0 | IIGP (Interferon-inducible GTPase) | PF05049 |
| ColloRR1 | A0A075AN70 | S_TK_X (Extension to Ser/Thr-type protein kinases) | SM000133 |
| UramaRR1 | Umbra1\|260787 | PDEase_I (3'5'-cyclic nucleotide phosphodiesterase) | PF00233 |

[a]In addition to the receiver domain necessary for designation as a response regulator.
[b]Likely an input domain. All others are likely output domains.
