# Supplementary material for: Predicted Functional and Structural Diversity of Receiver Domains in Fungal Two-Component Regulatory Systems
Source: mSphere. 2021 Oct 6;6(5):e00722-21. doi: 10.1128/mSphere.00722-21 (PMC8510515; doi:10.1128/mSphere.00722-21)
Supplement: TABLE S5 [file msphere.00722-21-st005.pdf]

Table S5. HHK group assignments made in this work

HHK	UniProtKB ID or JGI #	Assigned Group	Comment
CanguHK8	A0A1Y2H816	XIX	TM[a] and PAS domains
CheteHK10	Q6SLD7	XIV	Reassigned from group I
MlariHK7	Mellp2_3|1816084	IX	PAS domain
PfinnHHK1	A0A1Y1V617	XVI	TM and protein kinase domains
PostrHK7	A0A067NRD8	XIII	
ValloHK6	A0A4P9YS74	II	No TMs argues against XVII
RirreHK9	Gloin1|6899	II	
RirreHK10	Gloin1|21566	XII-B Rec1	C-terminal GAF domain
RirreHK11	Gloin1|35016	XII-B Rec2	
RirreHK12	Gloin1|77355	Unclassified	Truncated Rec domain
SplumHK4	Synplu1|252105	II	No TMs argues against XVII
SplumHK5	Synplu1|682744	II	No TMs argues against XVII
SroseHHK8	Sporo|23664	IX	
SroseHHK9	Sporo|28207	XII-A Rec2	
SroseHHK10	Sporo|33425	XIV	
TasahHHK7	K1VQS6	XIII	TMs, reassigned from group VII
TasahHHK11	K1W7S7	XIV	GAF domain
TasahHHK12	K1VIP4	XIII	
UramaHK13	Umbra1|255834	XII-A Rec2	PAS PAC domains

[a]TM = transmembrane regions
